# Genome-wide analysis of basic/helix-loop-helix gene family in peanut and assessment of its roles in pod development

**DOI:** 10.1371/journal.pone.0181843

**Published:** 2017-07-27

**Authors:** Chao Gao, Jianlei Sun, Chongqi Wang, Yumei Dong, Shouhua Xiao, Xingjun Wang, Zigao Jiao

**Affiliations:** 1 Shandong Key Laboratory of Greenhouse Vegetable Biology, Institute of Vegetables and Flowers, Shandong Academy of Agricultural Sciences, Jinan, China; 2 Biotechnology Research Center, Shandong Academy of Agricultural Sciences; Shandong Provincial Key laboratory of Crop Genetic Improvement, Ecology and Physiology, Jinan, China; University of Western Sydney, AUSTRALIA

## Abstract

The basic/helix-loop-helix (bHLH) proteins constitute a superfamily of transcription factors that are known to play a range of regulatory roles in eukaryotes. Over the past few decades, many bHLH family genes have been well-characterized in model plants, such as *Arabidopsis*, rice and tomato. However, the bHLH protein family in peanuts has not yet been systematically identified and characterized. Here, 132 and 129 bHLH proteins were identified from two wild ancestral diploid subgenomes of cultivated tetraploid peanuts, *Arachis duranensis* (AA) and *Arachis ipaensis* (BB), respectively. Phylogenetic analysis indicated that these *bHLH*s could be classified into 19 subfamilies. Distribution mapping results showed that peanut *bHLH* genes were randomly and unevenly distributed within the 10 AA chromosomes and 10 BB chromosomes. In addition, 120 *bHLH* gene pairs between the AA-subgenome and BB-subgenome were found to be orthologous and 101 of these pairs were highly syntenic in AA and BB chromosomes. Furthermore, we confirmed that 184 *bHLH* genes expressed in different tissues, 22 of which exhibited tissue-specific expression. Meanwhile, we identified 61 *bHLH* genes that may be potentially involved in peanut-specific subterranean. Our comprehensive genomic analysis provides a foundation for future functional dissection and understanding of the regulatory mechanisms of bHLH transcription factors in peanuts.

## Introduction

Basic/helix-loop-helix (bHLH) transcription factors are a superfamily of proteins that are widely distributed in all eukaryotic organisms and have been found to play an increasing number of functions in a wide range of essential metabolic, physiological and developmental processes, such as photosynthesis, light signaling, pigment biosynthesis, seed development and stress resistance [1–4]. The bHLH proteins among animals, yeasts, and plants are defined by two highly conserved domains, namely the basic region and the HLH region, which are approximately 60 amino acids in length [5,6]. The basic region contains approximately 15 amino acids and typically includes six basic residues, located at the N-terminus of the bHLH domain, which functions as a DNA binding motif [7]. The HLH region, located at the C-terminal end, is composed of two amphipathic α helices consisting of hydrophobic residues linked by a divergent loop. It functions as a dimerization domain, promoting protein-protein interactions and allowing for the formation of homodimeric or heterodimeric complexes to control gene transcription [8]. However, sequences outside of the highly conserved bHLH domain are usually quite divergent. bHLH proteins have been shown mainly to bind to a core DNA sequence motif called the E-box (5-CANNTG-3), with the palindromic G-box (5-CACGTG-3) being the most common form [9]. Several conserved amino acids within the basic region determine recognition of the core consensus site of different E-boxes [10].

With the release of an increasing number of genome sequences bHLH family genes have been identified in a range of plant species such as *Arabidopsis*, rice, tomato, Chinese cabbage and miltiorrhiza, suggesting that bHLH genes are present in almost all higher plants and have evolved specific functions with different biochemical properties [11–14]. To date, many plant bHLH proteins have also been functionally studied in detail. In maize, a bHLH protein (R protein) interacts with members of MYB family of proteins and together, they control anthocyanin biosynthesis and pigmentation in a tissue-specific manner [15]. In addition, a bHLH protein encoded by GLABRA3 (GL3, the closest homolog of the maize R gene) interacts with R2R3-MYB protein GLABROUS1, which has been shown to be involved in *Arabidopsis* trichrome differentiation [16]. Furthermore, bHLH family proteins have also been shown to participate in various biotic and abiotic stress responses. For example, Li detected several genes that respond to iron-deficiency and confirmed that two bHLH transcription factors in *Arabidopsis*, bHLH34 and bHLH104, play major roles in regulating iron homeostasis by activating the transcription of bHLH38/39/100/101 in iron deficient conditions [17]. In addition, a tomato *bHLH* gene, *SlybHLH131*, was found to be involved in resistance to yellow leaf curl virus infection through virus-induced gene silencing [13].

Peanut (*Arachis hypogaea* L.) is an important legume and widely grown throughout tropics and subtropics regions. Especially in Africa and Asia, the yield of peanut fruit accounts for more than 64% of the world’s total output [18]. The cultivated peanut is an allotetraploid (AABB-type genome; 2n = 4x = 40), probably derived from a single recent hybridization event between two diploid wild species (A*rachis duranensis* (AA-type genome; 2n = 2x = 20) and *Arachis ipaensis* (BB-type genome; 2n = 2x = 20)) through polyploidization and subsequent spontaneous genome duplication [19–21]. Peanut is a typical ‘aerial flower and subterranean fruit’ plant as peanut fruit development is suppressed under a normal day/night period and re-activated in dark conditions, indicating that light plays critical roles during early peanut pod development. Of interest to our research, several G-box binding bHLH proteins, the phytochrome interacting factors (PIF1, PIF3, PIF4, PIF5, PIF6, and PIF7 in *Arabidopsis*), are involved in controlling light-regulated gene expression through interaction preferentially with the active form of phytochrome B (PhyB) in *Arabidopsis* [22–24]. Given the potential roles of bHLH family proteins in regulating the expression of a broad range of genes at all phases of the plant life cycle, especially in the regulation of phytochrome-regulated light signaling pathways, it is of considerable interest to identify and characterize the bHLH protein family in peanuts.

Recently, whole genome sequencing of the wild type peanuts (AA- and BB-subgenomes) were completed and published (http://peanutbase.org/), providing an important reference for genome-wide identification and analysis of gene families [25,26]. However, the conservation and diversification of the bHLH gene family in peanuts has still not been reported. In this study, a hidden Markov model (HMM) that allows for the detection of the bHLH domain across highly divergent sequence was used to systematically identify and characterize the bHLH genes in peanut using A-subgenome and B-subgenome as references. Using this method, we have identified a total of 261 *bHLH* genes. Multiple sequence alignments, phylogenetic relationships, chromosome distribution patterns, DNA-binding activities and intron distribution patterns of these *bHLH* genes were also determined. Additionally, based on the expression patterns among different tissues and qPCR analyses, 61 *bHLH* genes that likely regulate pod development were identified.

## Materials and methods

### Plant materials and growth conditions

Plant materials were collected from cultivated peanut (Luhua-14) grown on the experimental farm of Shandong Academy of Agricultural Sciences with normal day/night period. Peanut materials including root, stem, leaf, flower and peg were collected at 60 days after seed germination. Six developmental stages of peanut gynophores were used in this study. Aerial grown gynophores, which were green or purple in color with a length of 3–5 cm were assigned as S1; peg grown in soil for about 3 d that were white in color and with no detectable ovary enlargement was assigned as S2; peg buried in soil for about 9 d with very small enlarged ovary was assigned as S3; peg buried in soil for about 15 d, 21 d, 27 d were assigned as S4, S5, S6, respectively. A 5 mm tip region of the gynophore was manually dissected, frozen in liquid nitrogen and stored at -80°C for the following experiments. Two biological replicates were prepared for each stage.

### Collection and identification of candidate bHLH genes in peanut

The whole genome sequence of the peanut AA-subgenome (Aradu.V14167.a1.M1) and BB-subgenome (Araip.K30076.a1.M1) were obtained from PeanutBase (http://peanutbase.org/) and the HMM sequence of the bHLH domain (PF00010) was downloaded from the pfam database (http://pfam.xfam.org/) and used as query to search for candidate peanut bHLH protein sequences in the AA and BB subgenomes using BLASTP (e-value < 0.001). To further confirm and filter out uncertain bHLH proteins, the predicted bHLH domains were examined using the SMART tool (http://smart.embl-heidelberg.de). All bHLH protein sequences of peanut used in this study are listed in S1 Table.

### Multiple sequence alignments, identification of conserved motifs and phylogenetic analysis

Multiple protein sequence alignments were performed using Clustal-omega (http://www.ebi.ac.uk/Tools/msa/clustalo/). To visualize the conserved motifs, the sequences were analyzed with WEBLOGO programs (http://weblogo.berkeley.edu). A phylogenetic tree was constructed using MEGA 7.0 (http://www.megasoftware.net) using the neighbor-joining method with the following parameters: pairwise deletion option, 1000 bootstrap replicates and Poisson correction distance [27]. The consensus tree showed only branches with a bootstrap consensus > 50. Maximum likelihood (ML) analyses were performed with PhyML version 3.0 (http://www.atgc-montpellier.fr/phyml) using the JTT model of amino acid substitution and the radial tree was drawn using FigTree v1.3.1 (http://tree.bio.ed.ac.uk/software/figtree).

### Location of *bHLH* genes on AA and BB chromosomes

To examine the chromosomal location of peanut *bHLH* genes, the start and end positions of each *bHLH* gene on each chromosome were obtained from the peanut database website (http://peanutbase.org/) via BLASTN, and a map was generated using MapInspect software (http://mapinspect.software.informer.com/).

### RNA-seq data collection and expression analysis of *bHLH* genes

To further characterize the function of peanut *bHLH* genes during peanut development, published RNA-seq data from 22 different tissues in cultivated peanut were downloaded from the National Center for Biotechnology Information (http://www.ncbi.nlm.nih.gov/) under BioProject PRJNA291488. A description of the peanut tissues is listed in S2 Table. The expression pattern of the *bHLH* genes in different tissues was determined using an R script based on the normalized RPKM (Reads Per Kilobase of exon model per Million mapped reads) values of all genes transformed to log2 (value + 1). A correlation analysis between orthologous regions of the AA- and BB-subgenomes was performed using SPSS software.

### RNA isolation and quantitative RT-PCR analysis

Total RNA was extracted from different peanut tissues using CTAB reagent. The reverse transcription reaction (20 μl) contained 2 μg DNase I-treated total RNA, 50 nM Oligo(dT) primer, 0.25 mM each of dNTPs, 50 units reverse transcriptase, 1×reverse transcriptase buffer and 4 units RNase inhibitor, according to the manufacturer`s protocol. The reactions were incubated at 42°C for 1 h and were terminated by incubation at 85°C for 5 min to inactivate the reverse transcriptase. *AhActin* was used as the internal control. SYBR Green PCR Master Mix (Bio-Rad) was used in all qRT-PCR reactions with an initial denaturing step at 95°C for 10 min, followed by 40 cycles of 95°C for 5 s, 65°C for 5 s and 72°C for 8 s. Three biological replicates were prepared for each sample and relative expression levels were calculated using the 2^-ΔΔCt^ method. Student’s t-test was used to determine whether the qRT-PCR results were statistically different between two samples (*P < 0.05). Primers used in all of the qRT-PCR experiments are listed in S3 Table.

## Results and discussion

### Identification of *bHLH* genes in two wild type peanuts

The *bHLH* gene family is one of the largest families in plants, and the members are only fewer than the MYB family [28]. In order to define the peanut *bHLH* gene family, in this study, a total of 132 and 129 bHLH proteins were identified in the AA- and BB-subgenomes, respectively, based on the Hidden Markov Model BLAST, according to the criteria developed by Atchley and Toledo-Ortiz [3,7]. To verify the reliability of our criteria, we performed simple modular architecture research tool (SMART) analysis of all 261 putative peanut bHLH protein sequences and found that the majority of these proteins (104, 78.7% in AA-subgenome and 94, 72.8% in BB-subgenome) had a typical bHLH domain. The proteins lacking the basic region may interact with other bHLH proteins to bind to the DNA motif. Cultivated peanut is an allotetraploid derived from two diploid wild species AA and BB that contain two closely related subgenomes. The number of *bHLH* genes between AA and BB are almost equal. This may be due to the fact that the genome size of AA and BB-subgenomes is highly similar, with a sequence similarity of 64% between AA and BB [25,26]. Compared with other transcription factor gene families, the bHLH gene family is the second largest family and has only a few less members than the MYB gene family. In previous studies, 147, 167, 159, 127, 230, 127, 289 and 319 bHLH genes were identified in *Arabidopsis*, rice, tomato, miltiorrhiza, Chinese cabbage, potato, maize and soybean, respectively [3,11,12,14,29,30]. The number of *bHLH* genes in each diploid wild peanut is similar to that found in *Arabidopsis*, rice, tomato, miltiorrhiza and potato, but was noticeably less than that found in Chinese cabbage, maize and soybean. This may be due to the large genome sizes of these plants or genome duplication. In precious reports, the number of bHLH proteins increased with plant evolution and genome duplication, suggesting that these proteins may play an important role in plant evolution.

### Multiple sequence alignments, conserved amino acid residues in the bHLH domains and DNA-binding activity prediction

**Fig 1:**
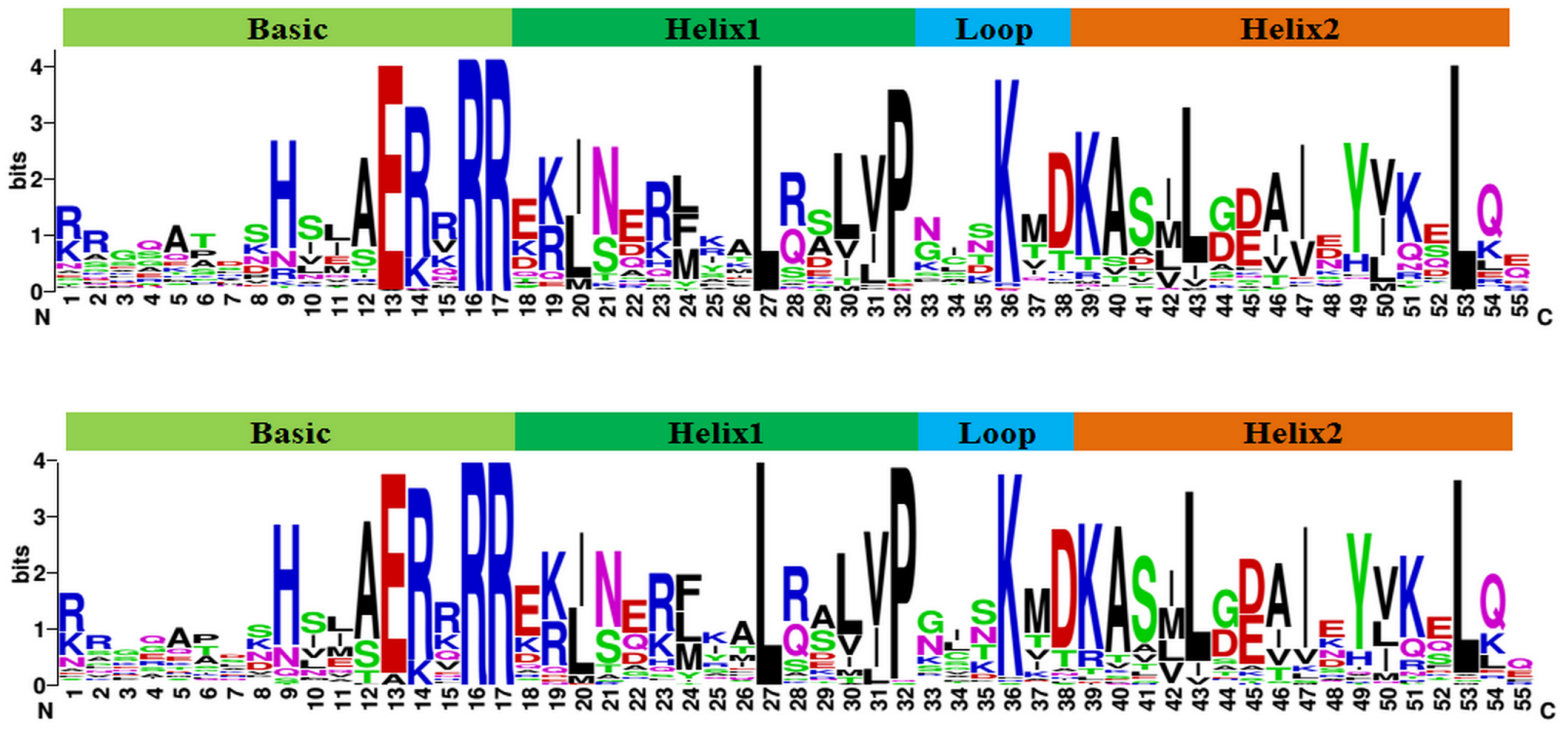
Sequence motif of the bHLH domain in peanut as determined by MEME.

To analyze the features of peanut bHLH protein domains, we conducted multiple protein sequence alignments of the bHLH domains from AA- and BB-subgenomes using Clustal-omega software (S1 Fig). The frequencies of the consensus amino acids within the bHLH domains were counted and are shown in Table 1. There are four conserved regions in the bHLH domain sequences for most of the bHLH proteins, including one basic region, two helix regions and one loop region (Fig 1). The basic regions have five conserved residues (His-9, Glu-13, Arg-14, Arg-16 and Arg-17) that were identical in at least 50% of the 132 AA-subgenome and 129 BB-subgenome bHLH domains (Fig 1A and 1B). The first helix region, the loop region and the second helix region have three conserved residues (Asn-21, Leu-27, and Pre-32), one conserved residue (Lys-36) and five conserved residues (Lys-39, Leu-43, Ile-47, Tyr-49, and Leu-53), respectively, that were identical in at least 50% of the 132 AA-subgenome and 129 BB-subgenome bHLH domains. Among these 14 conserved residues, six residues were present in more than 75% of sequences (Glu-13, Arg-16, Arg-17, Leu-27, Lys-36 and Leu-53) (Table 1). All of these 14 conserved residues have also been reported in other species, suggesting that these residues are extremely important for the function of bHLH proteins.

**Table 1:** Information on the consensus motif and conserved amino acid sequences in the bHLH domain.

Position in the Alignment	Region	AA-subgenome	BB-subgenome
**1**	Basic	R(30%), K(26%), N(7%)	R(30%), K(27%), N(5%)
**2**	Basic	R(30%)	R(25%)
**9**	Basic	H(60%)	H(55%)
**13**	Basic	E(81%)	E(79%)
**14**	Basic	R(62%), K(14%)	R(59%), K(16%)
**15**	Basic	R(30%), V(13%), K(10%)	R(31%), V(10%), K(8%)
**16**	Basic	R(80%)	R(78%)
**17**	Basic	R(80%)	R(78%)
**20**	Helix 1	I(43%), L(31%), M(13%)	I(41%), L(32%), M(12%)
**21**	Helix 1	N(54%), S(20%)	N(53%), S(20%)
**24**	Helix 1	L(30%), F(20%), M(19%)	F(32%), L(25%), M(21%)
**27**	Helix 1	L(86%)	L(85%)
**28**	Helix 1	R(36%), Q(33%)	R(34%), Q(32%)
**30**	Helix 1	L(48%)	L(46%)
**31**	Helix 1	V(45%)	V(44%)
**32**	Helix 1	P(68%)	P(65%)
**36**	Loop	K(79%)	K(75%)
**39**	Helix 2	K(50%), T(13%)	K(51%), T(13%)
**42**	Helix 2	I(34%), M(24%), V(19%)	I(32%), M(21%), V(19%)
**43**	Helix 2	L(72%), I(10%)	L(70%), I(11%)
**45**	Helix 2	D(42%), E(41%)	D(43%), E(44%)
**46**	Helix 2	A(49%), I(15%), V(15%)	A(45%), I(17%), V(15%)
**47**	Helix 2	I(57%), V(20%)	I(59%), V(18%)
**49**	Helix 2	Y(63%), H(10%)	Y(61%), H(11%)
**50**	Helix 2	V(43%), I(34%)	V(43%), L(30%)
**53**	Helix 2	L(83%)	L(82%)
**54**	Helix 2	Q(41%), K(15%), E(12%)	Q(39%), K(15%), E(10%)

**Fig 2:**
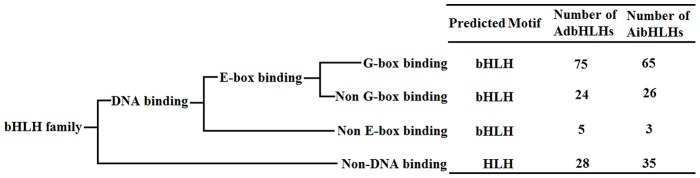
Statistical analysis of DNA-binding characteristics based on the bHLH domain in peanut.

The basic region of peanut bHLH protein that functions in DNA binding contains 17 residues. Using the criteria described by Massari and Murre, peanut bHLH proteins are divided into several categories based on sequence information in the basic bHLH region [1,9]. For the AA-subgenome, 104 DNA binding proteins and 28 non-DNA binding proteins were identified, while 94 DNA binding proteins and 35 non-DNA binding proteins were identified in the BB-subgenome (Fig 2). The DNA binding bHLH proteins were further divided into two groups, putative E-box-binding proteins and putative non-E-box-binding proteins, depending on the presence or absence of residues Glu-13 and Arg-16 in the basic region. Only five and three non-E-box-binding proteins were found in AA- and BB-subgenomes, respectively. The 99 and 91 E-box-binding proteins in the AA- and BB-subgenomes, respectively, can be further divided into two subgroups, G-box-binding proteins and non-G-box-binding proteins, according to the presence or absence of the His-9 residue. A total of 75 and 65 G-box-binding proteins were found in the AA- and BB-subgenomes, respectively.

### Intron distribution within the peanut bHLH domains

**Fig 3:**
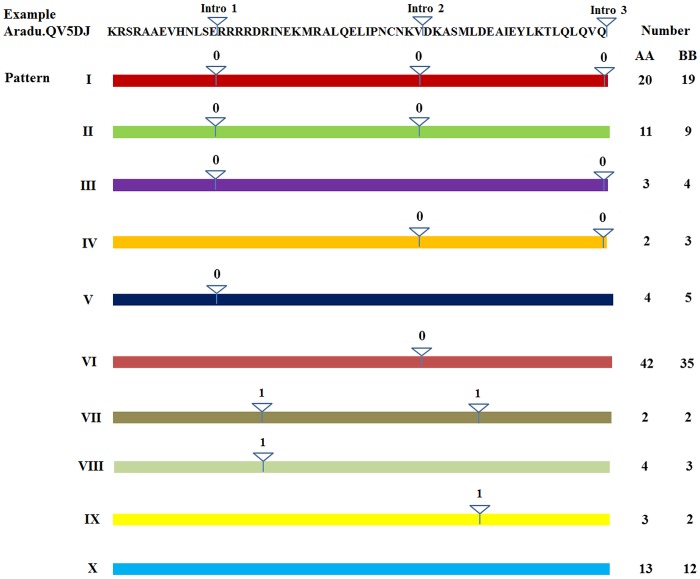
The distribution of introns within domains of peanut bHLH proteins. All patterns are color coded and defined as I to X. Introns are indicated by triangles and numbered (1 to 3) based on those present in the bHLH region of Aradu.QV5DJ (shown at the top). The numbers of proteins with each pattern is given at the right.

To analyze intron distribution within the coding sequence of the bHLH domain in all peanut bHLH genes reported here, we performed a multiple alignment between all the bHLH coding sequences and genome sequences using BLAST. Ten different distribution patterns (designated I to X), ranging from zero to three introns within the domain, were observed (Fig 3). Among the 104 AA-subgenome and 94 BB-subgenome *bHLH* genes, only 13 AA-subgenome and 12 BB-subgenome *bHLH* genes did not contain an intron in their bHLH domain region (pattern X). In contrast, 87.5% of AA-subgenome *bHLH* genes and 87.2% of BB-subgenome *bHLH* genes contained introns in the coding sequence of the bHLH domain. However, the sequence length and similarity of the introns differed among these bHLH domains, even at the same position. Among the nine patterns, pattern VI (including one intron) contained the most *bHLH* genes (42 of AA-subgenome and 35 of BB-subgenome) and pattern I (including three introns) was the second common in peanut, consistent with that found in tomato and rice, but different from that of *Arabidopsis*. In *Arabidopsis*, the most common pattern involves three introns in the bHLH region [3]. These results showed that intron sequences and their distribution varies among peanut, tomato, rice and *Arabidopsis* although their bHLH domains were conserved, and peanut bHLH proteins may have a more distant evolutionary relationship with *Arabidopsis* proteins than those of tomato and rice.

Furthermore, we also investigated the intron phases in the bHLH domains with respect to codons. The splicing of each intron is thought to occur in three different phases: phase 0, phase 1, or phase 2, depending on the splicing position in the codons. In phase 0, splicing occurs after the third nucleotide of the first codon; in phase 1, splicing occurs after the first nucleotide of the single codon; and in phase 2, splicing occurs after the second nucleotide [31]. As shown in Fig 3, all introns at the three conserved positions (indicated by white inverted triangles) were spliced at phase 0 (I-VI). The other introns with less conserved positions (VII, VIII and IX) were all spliced in phase 1. Interestingly, no splicing in phase 2 was detected in the bHLH domains of peanut, unlike that seen in both rice and *Arabidopsis* [11]. Such conserved splicing phases were also observed in the bHLH and MYB gene families of soybean, rice and *Arabidopsis* [11,32,33]. Therefore, our findings indicate that the splicing phase was highly conserved in peanut, as well as other higher plant species during the evolution of bHLH gene domains, and the introns in the bHLH domain may play an important role in the evolution of the bHLH gene family by means of unknown mechanisms.

### Phylogenetic analysis of peanut bHLH proteins

**Fig 4:**
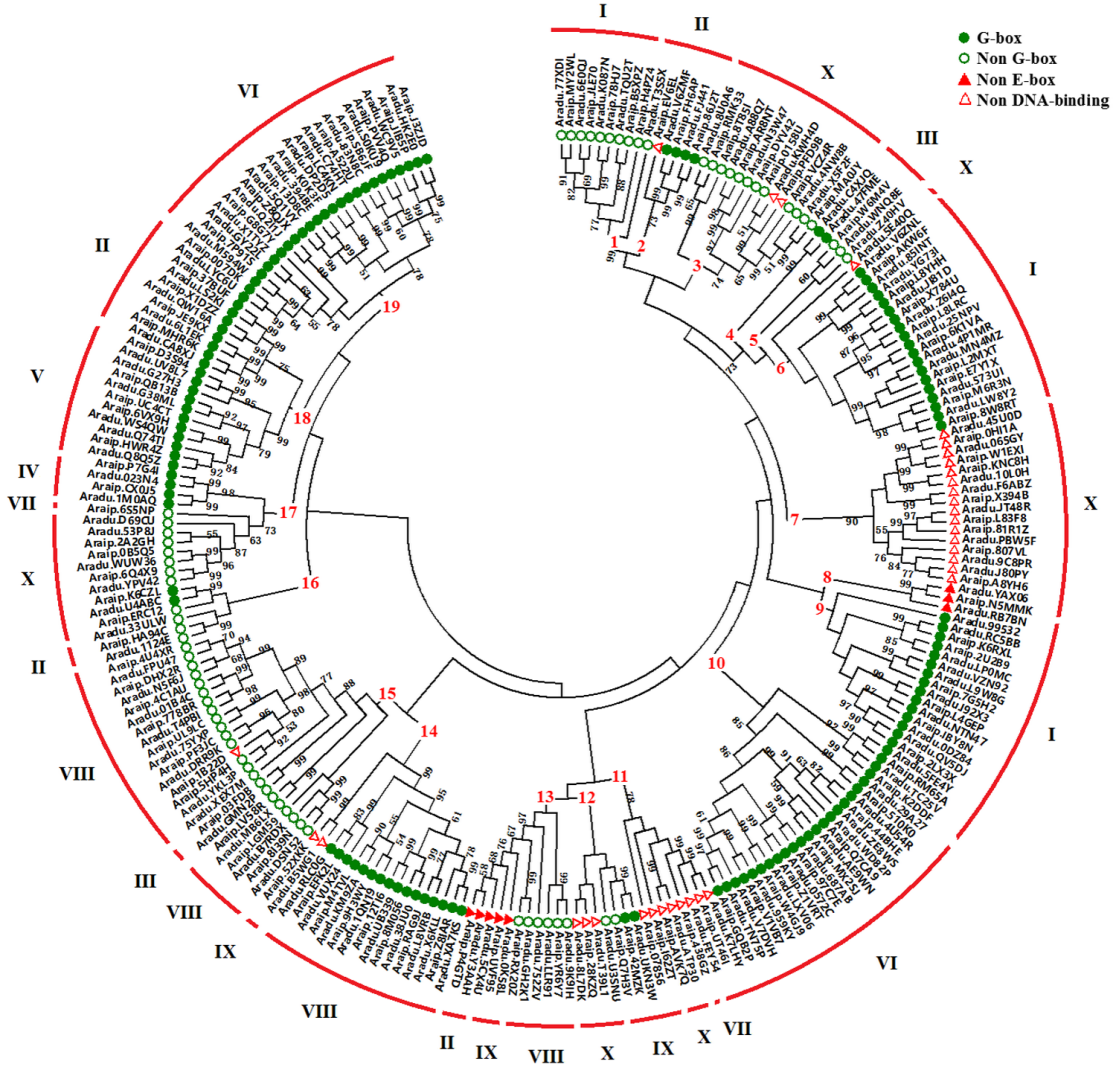
Phylogenetic tree constructed by the neighbor-joining method using bHLH domains in peanut, indicating the predicted DNA-binding activities and the intron distribution patterns. The phylogenetic tree was constructed using MEGA 7.0. The numbers are bootstrap values are based on 1,000 iterations. Only bootstrap values with greater than 50% support are indicated. Roman numerals correspond to the intron patterns as shown in Fig 3. The different shape on the left side of SlbHLH represents the predicted DNA-binding activity of each protein.

To identify the evolutionary relationships of the peanut bHLH proteins, a neighbor-joining (NJ) phylogenetic tree was generated using multiple sequence alignments of the conserved bHLH domains with a bootstrap analysis (1,000 replicates). The phylogenetic tree showed that all of the 261 peanut bHLH domains were subdivided into 19 subfamilies, designated as 1 to 19 (Fig 4), according to clades with at least 50% bootstrap support, consistent with the results showing that the bHLH superfamily in plants is usually composed of between 14 and 32 subfamilies, based on phylogenetic analysis of the bHLH region [34,35]. The genes with a G-box binding region were mostly clustered within subfamilies 6, 9–10, 14, and 17–19, whereas the genes with a non-DNA-binding region were grouped in subfamilies 7 and 11. In addition, different subfamilies can share the same intron distribution pattern. For example, genes in subfamilies 1, 6 and 9 have the same intron distribution pattern (pattern I), while subfamilies 10 and 19 belong to pattern VI. These results suggest that the pattern of intron distribution can also provide important evidence to support phylogenetic relationships within a gene family, and proteins within the same subfamily may share close evolutionary relationships.

Many bHLH proteins have been functionally characterized in model plants, such as *Arabidopsis*, rice and tomato. To further predict and annotate the function of peanut bHLH proteins and obtain information about the evolutionary history between peanut and other plants, a phylogenetic tree was generated using the alignment of full-length bHLH protein sequences of peanut, *Arabidopsis*, rice and tomato. This analysis generated 24 distinct subfamilies (designated as 1 to 24) according to the groups proposed by previous phylogenetic analyses of *Arabidopsis* and rice bHLH protein sequences (S2 Fig) [11]. The peanut bHLH proteins were unevenly distributed in all 24 subfamilies. However, our above results showed that only 19 subfamilies were found using peanut bHLH proteins alone. This difference may be attributed to more species and more protein sequences used in this phylogenetic tree. Generally, transcriptional regulators within the same clade may exhibit recent common evolutionary origins and conserved molecular functions [12]. Notably, seven peanut bHLH proteins including Aradu.5CX4U, Araip.P4GTD, Aradu.Y3AAH, Araip.UVF95, Aradu.GH2K1, Araip.RX20Z and Aradu.0K58L were highly orthologous to SlybHLH131, which has been proved to be involved in resisting to yellow leaf curl virus infection in tomato [13]. Furthermore, Aradu.QW16A, Araip.X1DZZ, Aradu.LS2KI and Araip.37BUF were orthologous to OsbHLH13 and OsbHLH16 that are involved in anthocyanin biosynthesis [36]. Aradu.1124E and Araip.HA94C were orthologous to OsbHLH164, which is critical for tapetum development [37], while Aradu.D69CU and Araip.6S5NP were orthologous to OsbHLH62, which is important for cold shock response [38]. These results suggest that the 15 peanut bHLH proteins within different subfamilies may have related molecular functions with their homologs in tomato or rice, which provides a foundation for future functional studies of bHLH proteins in peanut.

The phylogenetic analysis of full-length bHLH protein sequences between peanut and *Arabidopsis* indicated that the members of subfamily H are the most homologous to *Arabidopsis* PIF family proteins (S3 Fig). Specifically, Aradu.RC5BB and Aradu.LP0MC were highly orthologous to AtPIF7, while Aradu.I92X3 was orthologous to AtPIF4/AtPIF5. In addition, Aradu.QV5DJ, Aradu.YAX06 and Aradu.L9W8G were orthologous to AtPIF3. PIFs are a group of bHLH subfamily transcription factors that have recently been shown to act directly downstream of phytochromes and promote light-regulated growth and development in *Arabidopsis* [39–41]. Given that light plays fundamental roles in peanut development and pod formation, identifying components of the light signaling pathway will be of great significance to the study of peanut pod development mechanisms. In this study, six PIFs ranging from 446 to 740 AA in length were identified from the wild type AA-subgenome. Meanwhile, six PIFs ranging from 434 to 756 AA in length were also identified from BB-subgenome. Among them, five pairs were orthologous. General information about PIFs from wild and cultivated peanut is presented in S4 Table. All *Arabidopsis* and peanut PIF proteins were then analyzed for the presence of conserved motifs. A conserved APB motif, important for interaction with phyB, was found at the N-terminus of all 18 PIF proteins, and at least one bHLH domain involved in protein interaction and DNA binding was found in each PIF at the C-terminus (S4 Fig). Only four PIF proteins (Aradu.QV5DJ; Aradu.0DZ84; Araip.2LX3X; Araip.7G5H2) contained a conserved APA motif. In addition, a predicted nuclear localization signal peptide was found in most peanut PIFs. These functional motifs, as well as the phylogenetic analysis of PIFs between peanut and *Arabidopsis*, indicate that these 12 *bHLH*s could encode functional transcription factors involved in the light signal transduction pathway in peanut.

### Orthologues of *AdbHLH* and *AibHLH* genes are located in syntenic loci in the two wild type genomes

To determine the physical map positions of the *AdbHLH* and *AibHLH* genes on the peanut chromosome, the cDNA sequence of each OsbHLH gene was used to search the peanut genome database using BLASTN software. As shown in S5 Fig, 131 *AdbHLH* and 129 *AibHLH* genes were randomly and unevenly distributed across 10 AA chromosomes and 10 BB chromosomes. The distribution number of *bHLH* genes does not positively correlate with chromosome length. Chromosomes A03, A05 and A08 contained the same amount and largest number of *bHLH* genes (20), while chromosomes A04 and A10 contained the same amount and the least *bHLH* genes (6) in the AA-subgenome. In the BB-subgenome, both chromosome B03 and chromosome B09 contained the largest number of bHLH genes (19) and both chromosome B04 and chromosome B10 contained the lowest number of bHLH genes (7).

In addition, 120 *bHLH* orthologous gene pairs were detected between the AA-subgenome and BB-subgenome, according to the phylogenetic relationship and the sequence alignment of *AdbHLH* and *AibHLH* genes (Table 2). The identity and similarity of their full-length CDS sequences and protein sequences were both above 80%, which was consistent with the close relationship of the AA and BB-subgenomes. Among the orthologous gene pairs shared by the AA- and BB-subgenomes, 101 orthologous gene pairs were found on syntenic loci of the AA-subgenome and BB-subgenome. Notably, one AA *bHLH* gene (Aradu.8U0A6) had two corresponding orthologous genes in the BB-subgenome (Araip.RMK33 and Araip.8T85I), while three BB *bHLH* genes (Araip.44BHE, Araip.KI1I3 and Araip.RM65A) had more than one corresponding orthologous gene in the AA-subgenome, demonstrating that *bHLH* gene duplication events occurred universally in the two wild subgenomes, which are considered to be the raw materials for the evolution of new biological functions and played crucial roles in plant adaptation.

**Table 2:** The chromosomal location and identification of orthologous genes between AA-subgenome and BB-subgenome.

AA-subgenome	Chromosom	Starting position	End position	BB-subgenome	chromosom	Starting position	End position	CDS identity (%)	Protein identity (%)
Aradu.N5F6J	A03	123213397	123215577	Araip.DHX2R	B03	123867235	123869445	94.57	94.27
Aradu.QV5DJ	A09	21127504	21132997	Araip.2LX3X	B09	27025449	27031056	99.05	95.11
Aradu.I92X3	A07	4395707	4403324	Araip.L4GEP	B07	4261196	4268478	99.28	98.91
Aradu.RC5B**B**	A08	49042357	49045764	Araip.K6RXL	B10	1044901	1070158	97.48	93.75
Aradu.NTN47	A05	7762473	7765663	Araip.IBY8N	B05	8134164	8137390	97.81	97.99
Aradu.4U54R	A07	9293132	9294428	Araip.44BHE	B07	9285261	9287119	99.22	98.83
Aradu.ZE8WS	A07	75244941	75246494	Araip.44BHE	B07	9285261	9287119	98.7	95.51
Aradu.WD82P	A05	86030088	86031635	Araip.44BHE	B07	9285261	9287119	98.06	96.18
Aradu.AE9WN	A10	22120098	22122002	Araip.MX2SJ	B10	29630229	29631313	98.25	94.47
Aradu.CA8XJ	A06	14352906	14359399	Araip.MHR6K	B06	2329180	2336317	96.96	91.97
Aradu.9K9IH	A06	10166843	10167910	Araip.YR6Y7	B06	5637124	5638580	95.76	92.83
Aradu.KM9ZA	A06	1704104	1705517	Araip.M4TVL	B06	20280530	20282138	86.17	82.56
Aradu.TQU2T	A06	11953694	11956397	Araip.K3V8L	B03	130899070	130903237	97.52	95.07
Aradu.VZN92	A06	87333908	87340516	Araip.KI1I3	B01	10105809	10112122	91.02	88.89
Aradu.L9W8G	A06	71095585	71098273	Araip.KI1I3	B01	10105809	10112122	96.81	93.75
Aradu.RLC0G	A06	102769783	102772797	Araip.EFK2L	B06	126922402	126925335	89.46	86.06
Aradu.01B4C	A07	68460162	68461574	Araip.4C1AU	B07	33652691	33654351	99.37	98.94
Aradu.XA7KS	A07	63884567	63887247	Araip.Z8IAR	B07	42703138	42704374	98.95	98.65
Aradu.959KY	A10	104379282	104380857	Araip.V1VB7	B10	131077377	131078628	99.65	98.33
Aradu.**DP2D5**	A05	5669465	5671307	Araip.LC4KN	B05	5851374	5853400	98.23	96.99
Aradu.GMN2P	A09	115126735	115129951	Araip.03FDB	B09	142169130	142173416	90.87	89.58
Aradu.WS4QW	A02	11489982	11491999	Araip.6VX9H	B02	14875848	14877865	99.81	99.64
Aradu.G38ML	A05	938944	940960	Araip.QB13B	B05	927518	929537	99.76	99.26
Aradu.75YXP	A03	107601978	107605834	Araip.UL9LC	B03	108775829	108779121	87.36	84.41
Aradu.V6ZMF	A01	92963019	92966781	Araip.FH6AP	B01	134857345	134860895	99.35	99.02
Aradu.6L1EK	A02	88999373	89012161	Araip.JE9KX	B02	102551082	102557274	98.16	97.75
Aradu.S0KU9	A05	5676021	5677733	Araip.PVV4Q	B05	5858154	5859984	93.69	92.28
Aradu.UV8L7	A02	4340843	4342187	Araip.D3S94	B02	5524755	5525991	92.15	89.26
Aradu.WUW36	A10	4308451	4312402	Araip.0B5Q5	B10	6342232	6343412	99.23	99.1
Aradu.5Q1VY	A05	5650994	5652973	Araip.13D8C	B05	5834410	5836162	98.58	98.21
Aradu.QW16A	A03	101244033	101247558	Araip.X1DZZ	B03	103519080	103522574	95.64	94.29
Aradu.DRR9K	A08	48061749	48067471	Araip.PF3JC	B08	128554488	128559826	86.89	86.23
Aradu.K7LHY	A03	131184948	131187253	Araip.UT46I	B03	132161758	132164064	100	100
Aradu.T3S5X	A08	38437690	38440613	Araip.MY816	B08	107536756	107539018	92.46	89.31
Aradu.83N8C	A01	23818825	23821600	Araip.SB6JF	B01	29854077	29855671	89.65	86.25
Aradu.023N4	A03	38984267	38987093	Araip.P7G4I	B03	41339014	41343427	98.61	96.02
Aradu.DSN52	A04	29987509	29997721	Araip.8I39N	B04	28150382	28160574	88.25	86.42
Aradu.MB6LX	A08	27219784	27221158	Araip.LV58R	B08	4490284	4491952	95.06	94.75
Aradu.GH2**K1**	A08	31817103	31818948	Araip.RX20Z	B08	9920640	9922473	99.66	99.5
Aradu.Q8Q5Z	A08	47371984	47373861	Araip.HWR4Z	B02	4649892	4651764	87.29	84.32
Aradu.53P8J	A08	29959374	29961696	Araip.2A2GH	B08	7512296	7514586	89.15	86.57
Aradu.VUX24	A08	8677326	8680000	Araip.8JJ8B	B07	115260192	115263370	98.58	98.16
Aradu.X6KLL	A08	13571761	13573816	Araip.B6R33	B07	121797373	121799409	93.25	91.94
Aradu.L8VRB	A09	36737759	36739735	Araip.K2CBN	B09	44277246	44279223	98.29	98.03
Aradu.GY22L	A07	70863878	70865950	Araip.865PM	B09	276696	278046	99.61	99.47
Aradu.394BE	A01	24107574	24110292	Araip.K0K3F	B01	30217937	30223799	91.36	90.58
Aradu.YPV42	A03	23562037	23565650	Araip.6Q4X9	B03	26520167	26523626	99.48	99.15
Aradu.T4PBI	A08	15304565	15307528	Araip.778BR	B07	123477965	123480961	97.69	96.31
Aradu.B7RDX	A01	90749357	90752534	Araip.LGM59	B01	136638641	136642287	98.57	98.05
Aradu.5FE4Y	A01	104136727	104143924	Araip.RM65A	B01	120197405	120204803	98.86	98.31
Aradu.76WTQ	A05	14256031	14260383	Araip.RM65A	B01	120197405	120204803	98.26	97.45
Ar**adu.1QN19**	A02	4989975	4992481	Araip.9H3WY	B02	6253337	6255779	98.74	97.38
Aradu.K087N	A02	66678615	66681794	Araip.78HJ7	B02	77755307	77758153	96.35	95.64
Aradu.Y3AAH	A02	81071662	81076060	Araip.28KZQ	B02	92935898	92937449	83.95	80.47
Aradu.U4ABC	A10	57409972	57414243	Araip.K6CZL	B10	72699992	72703002	98.45	96.12
Aradu.5CX4U	A03	19548817	19550236	Araip.P4GTD	B03	22064567	22066022	92.06	89.64
Aradu.29A27	A05	209341	210323	Araip.510K0	B05	266513	267560	98.48	98.2
Aradu.TC25V	A04	119707748	119710708	Araip.K2DDF	B04	129766142	129769144	98.62	96.48
Aradu.WC9V5	A10	4762763	4765178	Araip.JIB5P	B10	6839043	6841764	97.69	97.45
Aradu.HK2E0	A03	117229115	117232108	Araip.J3ZJD	B02	105758542	105761458	99.24	99.11
Aradu.LYC6U	A07	70852466	70853604	Araip.007DK	B09	269214	270201	94.65	92.42
Aradu.FPU47	A05	757005	758654	Araip.4U4XR	B05	746210	747857	99.19	99.12
Aradu.33UL**W**	A04	100153216	100156399	Araip.B52UH	B04	109972789	109976507	99.13	98.72
Aradu.X1TYZ	A09	1154825	1159586	Araip.I1L37	B09	1342272	1346258	97.88	96.88
Aradu.4P1MR	A08	17382661	17385316	Araip.E7Y1X	B07	125315731	125318601	94.35	92.44
Aradu.38JU0	A05	1099766	1101296	Araip.8M056	B05	1081534	1082807	97.52	96.8
Aradu.MN4MZ	A06	109558099	109561275	Araip.L2MXT	B06	134206068	134209265	90.23	88.89
Aradu.LS2KI	A05	14588195	14593546	Araip.37BUF	B05	15322060	15325934	98.35	97.65
Aradu.Z40HV	A02	65874874	65881124	Araip.P76ZD	B02	77330077	77335721	99.03	98.33
Aradu.U3SNU	A07	5862390	5864259	Araip.B7KGV	B07	5488928	5490794	99.91	99.52
Aradu.573UI	A01	11180802	11184851	Araip.8W8RT	B01	636282	640342	99.83	99.04
Aradu.LW8Y2	A05	105232728	105237613	Araip.M6R3N	B05	98520552	98525231	98.57	97.59
Aradu.77XDI	A08	24276716	24279598	Araip.MY2WL	B08	2084004	2086453	99.85	99
A**radu.6E0QJ**	A03	3170734	3173105	Araip.JLE70	B03	5875559	5878134	97.38	96.22
Aradu.UKN3W	A05	55362909	55368355	Araip.42MZK	B05	93990530	93997158	95.84	91.48
Aradu.YG73I	A03	107170664	107172739	Araip.L8YHH	B03	108120839	108123374	95.62	94.92
Aradu.687AB	A08	32827122	32829163	Araip.97C7E	B02	7555503	7557891	99.87	99.59
Aradu.0572C	A07	66084894	66087281	Araip.Z1VRT	B07	38152435	38154293	98.35	96.24
Aradu.8U0A6	A08	23771933	23774264	Araip.RMK33	B08	1799226	1801832	85.39	83.18
Aradu.8U0A6	A08	23771933	23774264	Araip.8T85I	B08	89807834	89809919	84.57	82.23
Aradu.V6ZNL	A03	125315340	125317823	Araip.AKW6F	B03	126139564	126142290	91.05	89.08
Aradu.X5F2F	A06	110137734	110139757	Araip.MA0JY	B06	134873174	134875351	88.98	87.05
Aradu.1M0AQ	A01	90148274	90150596	Araip.CX0J5	B01	137028860	137032298	84.98	80.39
Aradu.FJ441	A09	49985480	49987376	Araip.86J2T	B09	65088189	65090077	98.79	97.98
Aradu.UB339	A05	104636237	104640020	Araip.12TI6	B05	105752877	105756562	99.37	98.62
Aradu.D69CU	A03	6936673	6945870	Araip.7B3CC	B03	10089696	10092649	100	100
Aradu.V7DV**H**	A09	120377026	120378791	Araip.IRQ3B	B09	131488063	131490111	85.39	83.39
Aradu.25NPV	A07	57713725	57715466	Araip.6K1VA	B07	62509003	62511083	99.69	99.21
Aradu.1124E	A03	117512276	117513177	Araip.HA94C	B02	105588372	105589261	99.78	99.06
Aradu.Z6I4Q	A03	4397458	4399566	Araip.L8LRC	B03	7165187	7167397	99.03	98.29
Aradu.0K58L	A08	31926537	31927699	Araip.WU1YN	B08	9996961	9997746	96.37	94.01
Aradu.TN75P	A05	104078493	104082669	Araip.GQB2P	B05	109099854	109104474	98.12	97.82
Aradu.LXV06	A03	120673178	120676797	Araip.W4GJ9	B03	121291051	121294003	98.63	97.29
Aradu.752ZV	A09	110546063	110547184	Araip.LP6IV	B09	146220284	146221793	92.46	91.79
Aradu.M594W	A09	1141725	1145405	Araip.7P91S	B09	1326764	1329521	89.95	88.53
Aradu.C4XJQ	A05	93484908	93486584	Araip.R44YW	B05	134684460	134686356	97.36	96.4
Aradu.47FME	A03	34615359	34616579	Araip.NA6B3	B03	37643849	37645334	98.51	97.36
Aradu.Q2I1J	A04	114428331	114431170	Araip.Z8QJX	B04	124221791	124224135	91.11	90.09
Aradu.WNQ8E	A06	11815777	11820835	Araip.W6M4V	B06	4109625	4114141	97.51	96.94
Aradu.YAX06	A06	6451194	6453590	Araip.N5MMK	B06	10356347	10358999	94.23	93.62
Aradu.8L7DK	A04	62751410	62757783	Araip.UYW0K	B04	76925354	76931849	98.89	97.73
Aradu.4NW8B	A09	112383645	112390463	Araip.VCZ4R	B09	144857745	144864744	99.2	98.79
Aradu.KWH4D	A03	121854600	121856521	Araip.0158U	B03	122440910	122442823	97.98	97.56
Aradu.ATP30	A04	20876961	20880191	Araip.AVK7Q	B04	20530497	20533734	100	100
Aradu.A88Q7	A05	7860229	7861087	Araip.AR8NT	B05	8285943	8286786	93.01	91.89
Aradu.V7BYP	A05	4471851	4474149	Araip.CAS5B	B05	4501468	4503100	84.82	83.28
Aradu.9C8PR	A10	4135298	4138047	Araip.A8YH6	B10	6050395	6052925	95.89	94.63
Aradu.JT48R	A03	24735645	24739346	Araip.L83F8	B03	27423471	27427329	84.23	82.1
Aradu.6VX3K	A02	138665	143152	Araip.AK8SS	B02	400803	405315	98.45	97.67
Aradu.RL2B3	A01	106355307	106358111	Araip.6S5NP	B01	113316638	113319457	99.02	98.22
Aradu.F6ABZ	A09	112670654	112672631	Araip.X394B	B09	144602711	144604943	96.57	95.12
Aradu.065GY	A09	120053875	120058233	Araip.W1EXI	B09	132284543	132288544	97.21	95.73
Aradu.N3W47	A08	6736203	6736977	Araip.AR8NT	B05	8285943	8286786	85.54	85.05
Aradu.T39L1	A08	43112520	43115382	Araip.74SLF	B08	120505450	120508682	87.12	86.15
Aradu.PBW5F	A08	35128967	35133230	Araip.81R1Z	B08	16648426	16654289	85.17	81.05
Aradu.45U0D	A08	952987	956174	Araip.0HI1A	B07	106383694	106387256	96.54	95.65
Aradu.B5WG1	A06	13540701	13544091	Araip.52XKK	B06	2452473	2455872	98.95	98.73
Aradu.99532	A06	110795071	110797180	Araip.LW9GQ	B06	135645448	135649363	99.24	98.48
Aradu.J80PY	A03	117542611	117545399	Araip.807VL	B02	105547540	105549808	91.93	89.68
Aradu.LP0MC	A06	101763848	101768612	Araip.2U2B9	B06	125913364	125917835	96.1	91.29

### Expression patterns of peanut *bHLH* genes in different tissues

**Fig 5:**
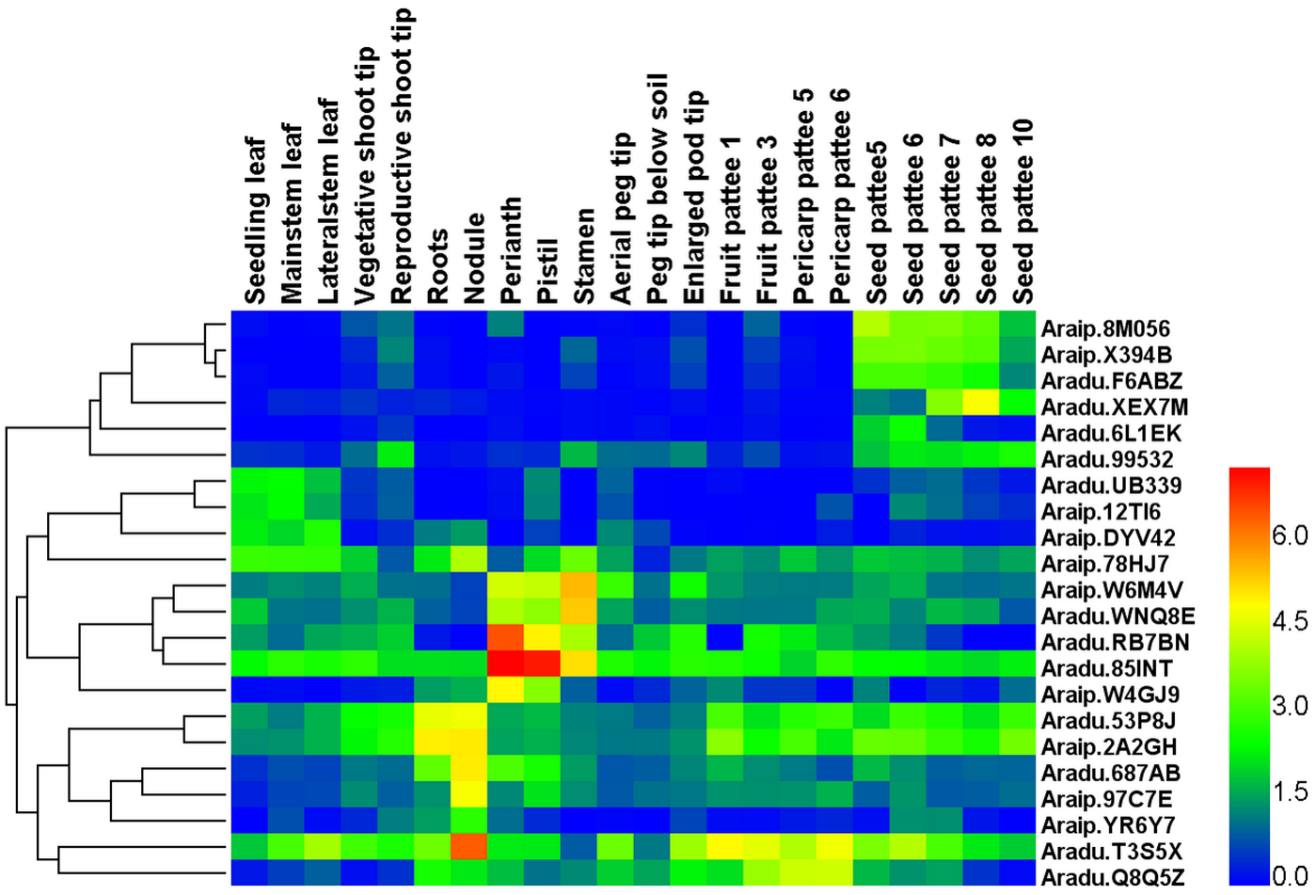
Clustering and differential expression analysis of peanut tissue-specific *bHLH* genes among 22 tissues representing the full development of peanut. In the heat map, the RPKM values were transformed to log2 (value + 1). The color scale is shown at the right and higher expression levels are shown in red.

To further understand the function of peanut bHLH proteins, the expression patterns of peanut *bHLH* genes among 22 samples, including 8 tissues, were analyzed, as well as 14 different developmental stages, according to the normalized RPKM data from RNA-seq. S5 Table shows the expression profiles of all bHLH genes in the 22 peanut tissues. Among the 132 *AdbHLH* and 129 *AibHLH* genes, 99 *AdbHLH* mRNAs and 84 *AibHLH* mRNAs had an RPKM value greater than 2 in at least one of the 22 samples, while the remaining 33 *AdbHLH* genes and 45 *AibHLH* genes were expressed at very low levels (RPKM ≤ 2) in all 22 samples. In particular, *Aradu*.*G38ML*, *Aradu*.*U3SNU*, *Araip*.*6Q4X9* and *Araip*.*QB13B* were constitutively produced at a relatively high level in all 22 samples, suggesting that these four *bHLH* genes perform a variety of functions in different tissues at multiple developmental stages of peanut. Furthermore, 22 *bHLH* genes showed preferential tissue-specific expression (the RPKM was greater than 2 fold higher in a particular tissue than in other tissues), including three genes in leaves (*Aradu*.*UB339*, *Araip*.*DYV42* and *Araip*.*12TI6*), five genes in flowers (*Aradu*.*RB7BN*, *Aradu*.*85INT*, *Aradu*.*WNQ8E*, *Araip*.*W6M4V* and *Araip*.*W4GJ9*), two genes in roots (*Aradu*.*53P8J* and *Araip*.*2A2GH*), five genes in nodules (*Aradu*.*687AB*, *Aradu*.*T3S5X*, *Araip*.*78HJ7*, *Araip*.*97C7E* and *Araip*.*YR6Y7*), one gene in the pericarp (*Aradu*.*Q8Q5Z*) and six genes in seeds (*Aradu*.*F6ABZ*, *Aradu*.*XEX7M*, *Aradu*.*6L1EK*, *Aradu*.*99532*, *Araip*.*8M056* and *Araip*.*X394B*) (Fig 5). The specific accumulation of these *bHLH* genes in a particular tissue suggests that they may play conserved regulatory roles in discrete cells, organs, or conditions. Given that the cultivated peanut is an allotetraploid that is derived from two diploid wild species, *Arachis duranensis* and *Arachis ipaensis*, it is very interesting to detect the orthologous expression of *AdbHLH*s and *AibHLH*s. As shown in S6 Table, 66 pairs of orthologous genes between *AdbHLH*s and *AibHLH*s exhibited similar expression patterns and similar expression levels, suggesting that these orthologous genes exhibited functional redundancy.

### Identification of pod development-related *bHLH* genes

Peanut flowers bloom above the ground, whereas its fruit develops underground. Following fertilization, the peanut zygote divides a few times to form a pre-embryo and embryonic development stops upon exposure to light or normal day/night periods. However, the ovary continues to develop and form a peg. Along with the elongation of the peg, the tip region (containing the embryo) of the peg is buried in the soil at which time peanut pod development resumes in darkness. Thus, the early development of the peanut pod is a complex, genetically programmed process involving the coordinated regulation of gene expression, seriously impacting on peanut production. Recent studies in model plant species have shown that the bHLH transcription factors participate in various plant developmental processes, such as root hair formation, anther development and axillary meristem generation [42]. However, there are no reports of the bHLH proteins in peanut pod development until now.

**Fig 6:**
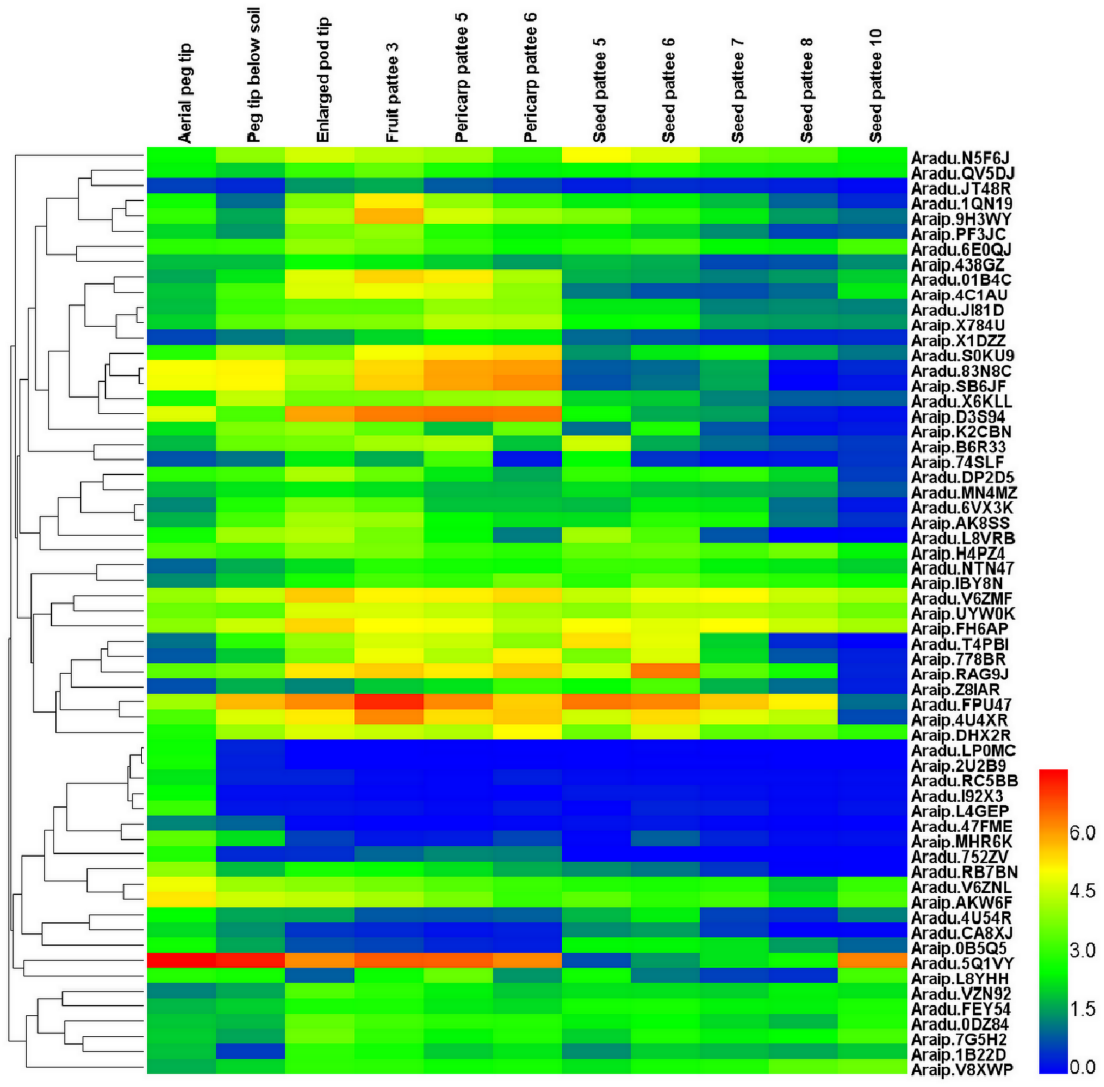
Clustering and differential expression analysis of peanut pod development-related *bHLH* genes among 22 tissues, representing the full development of peanut. In the heat map, the RPKM values were transformed to log2 (value + 1). The color scale is shown at the right and higher expression levels are shown in red.

**Fig 7:**
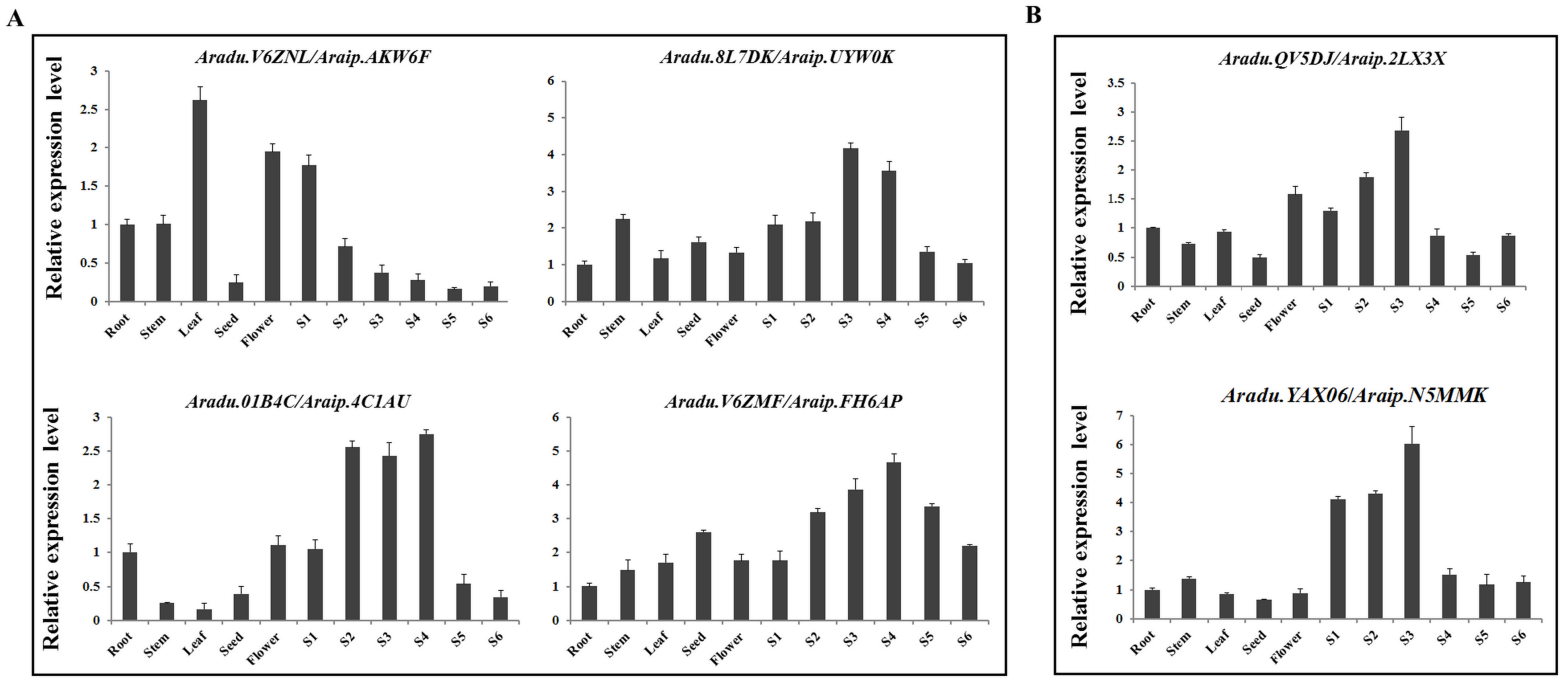
Relative expression analyses of four pod development-related *bHLH* genes and two *PIF*s by qRT-PCR among different tissues and different developmental stages of pod. (A) Expression analysis of pod development-related *bHLH* genes. (B) Expression analysis of peanut PIF genes. The levels in the roots were arbitrarily set to 1. Error bars represent the standard deviations of three biological replicates.

In order to assess the potential regulatory role of *bHLH*s in this peanut-specific process and predict candidate *bHLHs* that may function in the regulation of gene expression during early pod development, we further investigated peanut *bHLH* expression among the early developmental stages. We identified 31 *AdbHLH* and 30 *AibHLH* genes that showed a gradual increase or decrease in expression, along with the early developmental process based on the gene expression data (Fig 6). To validate the bioinformatic data, qRT-PCR was performed to examine the expression of several *bHLH*s that may be related to early pod development and results were in agreement with the sequencing data (Fig 7A). The high and differential expression of these genes in early developmental stage of pod, or peanut fruit, may directly contribute to pod formation and development in peanut. Furthermore, the expression of two PIFs in root, stem, leaf, flower, seed and different developmental stages of peanut gynophore were examined using qRT-PCR (Fig 7B). The results showed that *Aradu*.*QV5DJ*/*Araip*.*2LX3X* was expressed in all of these tissues with higher levels seen in flowers and early developmental stages of gynophore than was seen in other tissues. The accumulation of *Aradu*.*YAX06*/*Araip*.*N5MMK* in S1, S2 and S3 of gynophores was significantly higher than that in other tissues (Fig 7B), implying that these two genes may serve broader functions than other *AhPIFs* in light signaling, cell division, differentiation and morphogenesis during early embryo development and pod formation.

## Conclusions

Although many bHLH family genes have been identified in various plants, only a small number have been functionally characterized. In the past few decades, the regulation of growth and development, stress resistance, metabolism, light and hormone signaling by bHLH transcription factors has been reported in various plants. However, until now no reports about the *bHLH* genes in peanut have been made. This study is the first comprehensive and systematic analysis of bHLH transcription factors based on the entire genome sequence of the wild peanuts. In total, 261 bHLH transcription factors were identified in the wild peanut genome. The structure, classification, expression patterns among different tissues and comparative analyses of this gene family between peanut and *Arabidopsis* will help to identify candidate bHLH transcription factors potentially involved in regulating peanut pod development and provide basic resources for further study of *bHLH* genes in peanut. Further detailed experimental investigation is required to reveal the roles and molecular mechanisms underlying the regulation of *bHLH*s (particularly the PIF subfamily genes) in the developmental and physiological processes during early pod formation and embryo development.

## Supporting information

S1 Table Amino acid sequences of all bHLH proteins in peanut. (DOCX)

S2 Table Description of peanut tissues collected for RNA-seq analysis. (XLSX)

S3 Table Oligonucleotide primer sequences used for qRT-PCR. (XLS)

S4 Table Information of PIFs identified in wild AA- and BB-subgenomes. (DOCX)

S5 Table RPKM values of all peanut *bHLH* genes among 22 tissues that represent the full development of peanut. (XLSX)

S6 Table RPKM values of 66 pairs of gene orthologues between the AA-subgenome and BB-subgenome. (XLSX)

S1 Fig Multiple sequence alignment of the peanut bHLH domains. (TIFF)

S2 Fig Phylogenetic analysis of bHLH proteins of peanut, *Arabidopsis*, tomato and rice. (TIFF)

S3 Fig Phylogenetic analysis of bHLH proteins of peanut and *Arabidopsis*. (TIFF)

S4 Fig The distribution of conserved motifs in each PIF gene. The relative positions of each conserved motif within the PIF protein are shown in color.
(TIFF)

S5 Fig Location of peanut bHLH genes on the chromosomes using MapInspect software. The chromosome numbers are shown at the top of each chromosome (black bars). The location of each bHLH gene is indicated by a line.
(TIFF)

## Abbreviations

bHLH: basic/helix-loop-helix
HMM: Hidden Markov Model
PhyB: Phytochrome B
PIFs: Phytochrome interacting factors
qPCR: quantitative Reverse transcription Polymerase Chain Reaction
RPKM: Reads Per Kilobase of transcript per Million mapped reads
SMART: Simple Modular Architecture Research Tool
