# Supplementary material for: Genome-wide analysis of basic/helix-loop-helix gene family in peanut and assessment of its roles in pod development
Source: PLoS One. 2017 Jul 27;12(7):e0181843. doi: 10.1371/journal.pone.0181843 (PMC5531549; doi:10.1371/journal.pone.0181843)
Supplement: S1 Table — (DOCX) [file pone.0181843.s001.docx]

>Aradu.J80PY.1 genotype-assembly-annot=V14167.a1.M1

MADDEDFQGAAGICGENWWRSIDSTRSVFPPLTSSSPSSTSPCSVAPPTDTRTWPSSDDHHHCFMDLKAPACDIISNNNNNNYHMSFHDAQEKPHNNKTTTNESQSGSMLIDSTFQMMGFGLSSPNWDHQSLLENNNSQIIQKDYWSRKNFGTQQQQVSSTMDVFKRMNNQEFSSSTYGYPSSTLIQTLYDPQQQPQRSSSSSSSLFTNRSMSYSSSSTVNYHHASDEVSLSPITWPKLFSSSSSSSSSSLSLLPPMAKHPDNNNIGLHFSNNNTPFWNASSDALHDIRAGAFPFEEEDNKPNSPIILLNKLKSEELCVDSVKKNNNVCSSPPPFKRARIETPPPLPTFKVRKEKLGDRVTALQQLVSPFGKTDTASVLHEAIEYIKFLHDQVSVLSTPYMKSGASIQPNQECNDWKESPEVAKRDLRSRGLCLVPISSTFPVATEVSADFWTPTLSFGGALLR.

>Aradu.N5F6J.1 genotype-assembly-annot=V14167.a1.M1

MELSQLGFLEELMLAPRKDTWSSCTSNDVFSPSSWNYFDPFLDTPSSSSFPPSAFSPPPPDCPFTTSYPFPDDAFTMPDLDSSYTTKYEYEYDDAPPPPLPHNHQDLDDDNNNPSVLDDEDAGFLGCKVEEQAVVDVQVHVPVVFNMGMCGIGEKKNKSKKLEGQPSKNLMAERRRRKRLNDRLSMLRSIVPKISKMDRTSILGDTIDYMKELLERINKLQEEEVEEGTSQQNLLGVSKELKPNEAMVRNSPKFDVERRDKDTRISICCATKPGLLLSTVNTLEALGLEIQQCVISSFNDFSMQASCSEVSEQRNCVDPEEIKQALFRNAGYGGRCL.

>Aradu.QV5DJ.1 genotype-assembly-annot=V14167.a1.M1

MPLYELYRLSREKLDEEINGTRATDQSRSSSPEKDFFELIWENGNILTTQGQSSRAKKSPPRRSLPSHCLPSHSPKGRDRDAGYVNNPRVGKSGDLDTGLNEISMSVPSTEVDLGHDDDVIPWLDYTMDGSLQNEYGSNFLHELSGVTDQDLPSNHFSLVDKSSGNQVFRDSHKNSAEQSNFSSVSSTGVDETTRPKASTVESYLPSSFISVRPRVSDVTANNTSNAMLHPPVTEIPSSSSDFSSLKMQKQDQVIPSNGSSVMNFSHFARPAAIVRANLQNIGLKSVSSSTRSENVESMNKGAVVPSSNLPESTLADSCSECPKVLMGNNEKAVEQSRDDLKLLESKSLEQNIAGSKQLDPTCKEKAIKIDQTSNRVLGETATNTQIAAERSTELVVASSSVGSGNCADRGSDDPIQNLKRKNRDTEDSEWHSDDVEEESVGVKKAAAGRGASGSKRSRAAEVHNLSERRRRDRINEKMRALQELIPNCNKVDKASMLDEAIEYLKTLQLQVQMMSMGAGLYMHPMMLPHGMQHMHAPHLAPFSPMAYGMQMGLGMGYGMAMPDMNGVSSRFPMVQVPQMQGTHVPVAQMSGPTAMVRSNTQGFGVPGQGFPLPLPRAPLFPFSGGPVMNSSSALGLRPCGTTGLSQTADLASTSGLKDPSPNTDSLVKQSTGGGGCDSTSQMPIQCEAAATTVGFEQSSMVHNSSHTSEANDSGTLNPDKEDNNLVTGYDD.

>Aradu.LP0MC.1 genotype-assembly-annot=V14167.a1.M1

MSQCVPSWEVEDTNPPPPRPSLRSNSNSTIPDVPMLDYEVAELTWENGQLSMHGLGFPRVPTKSSAVTTTANKYTWEKPRASGTLESIVNQATTTPQNAKSTTFHGFGGGGNGGGVYENLLVPWVDPHRVPATVISPGTSNTMTMDALVPCSNRVEEQRTPQVMDSISGGLGPCMAGRTTRMGSGDAKDGGALEKRAAMVRRVPAPTVSAHEMSSRDQSVSGSATFGRESRHVTLDTCEREFGVGFTTSTSMGSPENTSSAKQCTKTNTIDDHDSVCHSRSMKRRDKINQRMKTLQKLVPNSSKTDKASMLDEVIEYLKQLQAQVQMVNRINMSSMMLPMTMQQQLQMSMMAPMGMGMGMGMGMGMAGMGMGMDMNTLSRTNIPPGIPPVLHPSPFMPMPSWDAAATATAAAATGAADRLQGPTPSAMPDHLSSFFGCPSQVSKCSIINLYLLS.

>Aradu.I92X3.1 genotype-assembly-annot=V14167.a1.M1

MMNNNSTIPGWNFESDACVINQKSPIGLDQDLVELLWKNGQVVLSSQTQRKPVQKSDASSTLRNGVHCGNSSSNLSQQEDETASWIQYQVEDHPMGQELCSSNLLSQLTPCEVEAYKPIKQLEEPKFAKFATPNANLSPQQQNNNTKPINPMPAPRFQVAPDSSNQKTNDFVGGSSSSQKVPKFSHHFSAPAQQKFREKVNGNNNVLQSEGRECSVMTVGSSHCGSNNIIPQEQDTTFSEAVKDCVQRSVPNWSNNKGKSSEMIEATVTSSSGGSGSSLGKTTCSLSTGNHLSLKRKITDADESEEQSEATQPKSGVGNKTSQRSGSSRRNRAAEVHNLSERRRRDRINEKMRTLQQLIPNSNK1TDKASMLEEAIEYLKSLQLQLQVMWMGAGMTPVMFPGIQHYMSQMSAMGMAAASQPSLPSMQNPMQIPRMPLDQSNIPSTVSVPHHQTPSNQALMCQNPVLGAFNYQNHQMQNPCLADQYARYMAYHHLMQTASQPMNVFGYGSQAVQQSQTMMPPSNNNGTMNVAPNFEDAITAKMGTSTFQQLNNN.

>Aradu.RC5BB.1 genotype-assembly-annot=V14167.a1.M1

MANNYDEIAELTWENGQISMHGLSGLDPTSQKKPTWVNRAHDTLESIVQQATCKNKKSKLTIKDNHAYVDVPTTTSSIVASSGEHHQMVPTLSRKRSHSSYSDQQQHRRDVNYVSINNTNRKCGVATASVTFCRDNNDVTTMMTWPSLDSGPRSLKNDKILEEDSACQSGSEVRNNENDRDGGKGETGQSKSSSVVRRNRTAAVHNQSERRRRDRINQKMKALQRLVPNANK2TDKASMLDEVIKYLKQLQAQIEMMMSVSMPQMIMQQQLQMSMLARSMANNAPNPIRPLFPQLIQPTTTIGAANTASAPMFLAPSLMIPSSTNASIPLPSASYGAATFAQPLNMDMLNNMAAFYSQQMNHHQNNQIKP.

>Aradu.NTN47.1 genotype-assembly-annot=V14167.a1.M1

MGDHMYNYDKNLSSSSSSQDEISLFLRQILLRSSQPHQDSPPPPQQQQQQQLPSSSSSSCFPSTSQLQDGKISALGSTPAGFLSASSMKGHGGNSAPNVSSSSVGLSANDTDDYDCESEEGVEALAEDVPAKPVPSRSSSKRSRAAEVHNLSEKRRRSRINEKMKALQNLIPNSNKTDKASMLDEAIEYLKQLQLQVQVCVTFTSCIIVICGWPNLPQAVDS.

>Aradu.99532.1 genotype-assembly-annot=V14167.a1.M1

MGGCNGGSFPCDSSEDFSALFNQLLSPPPQPNLTITNRRRRSRINEKMKALQNLIPNSNKTDKASMLDEAIEYLKQLQLQVQMLMMRNGLSLHPMSLPGGLRPMILPHTGVDFDEGNRFQNSINGGASSSYTADESLVARSPFGFHRPCSISNQSAAMPSLTNLATSDNSSSFQSSIKVHV.

>Aradu.4U54R.1 genotype-assembly-annot=V14167.a1.M1

MENYYYSGWPQSQCNNSAPNSNNNSSFSVPTQIVPHAYDSAFASLQFQYPWPLAFEGFPEDRAASASKSHSQAEKRRRDRINAQLATLRKLIPKSDKMDKAALLGSVVDHVKDLKRKAMDVSKSITVPSETDEVTIIECDPDQDESYAKVKILKHNIVISVCCDDRPELFNELIQVLKGLRLTAVKADIASVGGRIKSILVLCSKENEEGGDGVCLNTLKQSLKSAVNKIASSSMTSNCPTRSKRQRFFLPSHFIQ.

>Aradu.ZE8WS.1 genotype-assembly-annot=V14167.a1.M1

MVVQPSMPARPSSSPLPLLCGPFPFSHSPFYFLSFLLQITVCVCVFRIGVAAAAATIVNIPTQIVPHAYDSAFASLQFQYPWPLAFEGFPEDRAASASKSHSQAEKRRRDRINAQLATLRKLIPKSDKMDKAALLGNVVYHVKDLKRKAMDVSKSITVPSEIDEVTIIECDPDQDESYAKVKILKHNIVISVCCDDRPELFSELIQVLKGLRLINQDHNIFRSSKQLTEVAKQKQQNILKIKNIRKSESKIKKAA.

>Aradu.AE9WN.1 genotype-assembly-annot=V14167.a1.M1

MENYDHCSLFLDPSQTLPHVSDCNSLHHQFGGEFHSWPAPVEMVAGDRAASASKSHSQAEKRRRDRINAQLTSLRKLIPKSDKMDKAALLGSVIEQVKDLKRKAIEVSKAITVPTESDEITIEYDPSARDESLKMKNKVVIKASVCCDDRPELFSELIQVLKGLRLTAVRADIASVGGRMKGTFLLCSKDNEKVVCLGTLKHSLKSVVTKIASSSIATNWPTRSKRQKSILKTEW.

>Aradu.CA8XJ.1 genotype-assembly-annot=V14167.a1.M1

MPSCSRVQSMLQAAVQSLHWTYTIFWQLSPQQRVLRWSDGFYNGEIKVRRTVEAIEANKSNSNNEEASLQRSNQLRDLYDLLLSDRQGDLIFAADPPATPRPCTALSPEDLSESEWYYLMCVSFSFAPGVGLPGKAYARRQHVWLQGANEADTKTFSRAILAKSAHVKTVVCIPMLDGVVELGTTDKVKEDINLIKHIKSFFIGDHHRPQPKHALSENSASNPITEEAQLLLDSETATLPQSKLKELVMTEDSSRAGSPDDGSNRLNSAGLELLPPESAASLELLHGDEYDNNSEAETSHLCTEQEGHTHYSKTVSTILEGSSGHRPDAVCGLLSFQSAFATWSSRAYNCHQVTVVENPQRLLKNILFRVPYLHRKQNRHNSNNNSAKTLEQLERTMMPSPQDDLISSHVAAERRRREKLNEKFITLRSLVPYVTKMDKASILGDTIEYVKQLHEKVKKLEEEDKRRRSGKKRKERAVEVSVIEGEILLEVECMNREGLLLDLMRALKEVGIEVRGVKSSFKGDDERVFVVELRGKVKENGKRNNNNNKKKKISIMEVKKVLRNIINPHHPL.

>Aradu.9K9IH.1 genotype-assembly-annot=V14167.a1.M1

MSMMLALSSPTSMFPNNMEWPLELEEEELSHSHHEYFNSEEYPLLSSSMAKKFNHNANERHRRKKINALYSSLRSILPVADQTKKMSIPATISRVLKYIPELQQQVEELIKKKEELLLRISRQGDINNDDAAAAAAMNKKGHHHQNSSGFLVSSSTISDSEVSIQIISSYGIQKCQVSEILVCLENHYALQLLNASSFDTFGGRLFYNLHFQMEMAHTLDTEVLSEKILSICEKHQRI.YLCNVNN.T.YKYFLFLVIA.

>Aradu.KM9ZA.1 genotype-assembly-annot=V14167.a1.M1

MERLQLQGPLDSSLFGEHLEVNCLEQGFVDRESNFKVKEDEHEEQTLISSLEGNMPFLQMLQSVESPQHLKKPWEGTPYIPTLESEINEFEQYTRSGSDAATRLSKHHHQERVRKRKRGRESKDKDKEEVENQRMTHIAVERNRRRQMNEHLSVLKSLMHPSYIQRGDHASIIGGAIDFVKELEQLVESLEAQRRMRNKEEEDGGKEEASGGNEAKAERRSKVGGIEVSVIQSHVNLRIQCEKRAGLLINAIVALENLRLPILHLNITSSDSSVLYSFSLKIEEDSKLGSANEIAEAVDKILTSLRH.

>Aradu.B5WG1.1 genotype-assembly-annot=V14167.a1.M1

MESKMSFTSGGTSCHVTGSAAEDDSNMQDAVATEEGSGSQWSFLPKPSHHNYVQYLTESSNYLPKTEDMIGSNYAALGFGNDANGTNMNFFCNDPGKAYAFKTEVHSSNEVDIGKQVGYWRPAESEVQQEAMQFESTQHHHPLYPESPASWTPEATGDNPNVSRLDPSVMAGTPSFLPKPARQKASDRQRRQRIADNLKALHDLLPNQAEGSQAYILDDIIDHVKYLQLQLKELSGSRLQAESTAIPLVFHEGYGHYIDQQKLNEPLEEMMGKLLEENPASASQLLENNGLVLLPMELVQDLHQSMQIFGNSNALV.

>Aradu.TQU2T.1 genotype-assembly-annot=V14167.a1.M1

MDSSSRYHQQQLNSSSSSASGLLRFRSAPPSVLEQLQVVEGSCSSEQERSSSSSFLRFFSSNKNKPSSTTKPPSLSLMNSNHRFTRLHNSSTAPSASTSSGTDLPRQSTFPASHFSFHDNNGYDTNANTMTKGVGNYSGSDELSLSTMNRFNNQISFSSSRSPSSSATATNSNRNGGLFFPNYGSWNETSYKRDDRNRIHKLIFDANQNEEFGNNKVVDTLSHQLSFPKAEPEMFAMENMIHFPLSDSVPCKIRAKRGCATHPRSIAERVRRTRISERMKKLQELVPNMDKQASTADMLDMAVGYIKDLQKQFKVRN.

>Aradu.VZN92.1 genotype-assembly-annot=V14167.a1.M1

MHDLLMTQKEKNRRCSLWPQNPIDFSEILEAFSIKVKSMIDYLLRCMARSALVLGLKPHTYRSVITVLLQDNEVEGRSLILACQIHVTRMLNTHLLAAASNAVVAAFNELLRFGFDLKIRQRSFVAAHLIAIIYLCTITSNTVVAAFYDLIFNLAHLLTSASNAVVAAFNKLLQFGFTISVTSNSHGIVEELIGFGARVHACARNEAELTKEKLMDSVCSEFQGKLNILDIEEESVGVKKACPTRDVKRSRSAEVHNLSERKRRDRINEKMRALQDLIPNCNKVHLFILSNLEPYLLLLEPKMQPEKQRNETKRQEAWQVYGALLCAVGQCMHEKAKIFSNLLSPTRAFSRPKAMIAMSSMTSLCCNEIYTGVSK

>Aradu.RLC0G.1 genotype-assembly-annot=V14167.a1.M1

METEKLPAMDNNTLLSETFLNTTQFGGEDLFSILESLEDLNSPPPPPRKRQKVTDADGEERVLSSHITVERNRRKQMNHHLSVLRSLMPSFYVKRGDQASIIGGVVDYINELQQVLQCLEAKKNRKVFYTDNVLSPRLIIPSPKLGPRILTLPPISPRTPQPGSPWLHSIEPSPSSSGSDNMNELVANSKSMIADVEVKFCGPHVLVKTVSPRIPGQAMRIVSALQDLALEILHLTITTSDETMLYSFTIKIGIECQLSAEELAQHIQQTFC.

>Aradu.01B4C.1 genotype-assembly-annot=V14167.a1.M1

MLVEEELQSLEMQRECKMEASQSSSPPVFNSERKNNNRSKKVEGQPSKNLMAERRRRKRLNDRLSMLRSIVPKISKMDRTSILGDTIDYMKELLDKINTLKQEIQVVDDSNGILSNDKHPNDILVRNSPKFDVERRNNNGDTRVEICCGGKPGMLLSTVNTLEALGLDIQHCVISCFNDFTMQASCSEIQL

>Aradu.XA7KS.1 genotype-assembly-annot=V14167.a1.M1

MALEAVVFPQDPLSYTCNKDNYLYSIAASGGPWSNHEELYGNNVTDSMDQWDSYNSNSSPEPCITTIDHDQASVPGANNSPLAPVEAATSSNTIAAGAASTGRRKRRRTKSVKNKEEIENQRMTHIAVERNRRKQMNEYLAVLRSLMPPSYVQRGDQASIIGGAINFVKELEQLVQCMNGQKKTKQEHHNNTVPFAEFFMFPQYSTHATRYCNNDNTVYPPCVEAATKKPSWSSTAAVDIEVSLVDSHANMKILSKKQHGLLVKMVIGLQNLGFTILHLNVTTANDMVLTSVSVKVEEGSQLNTVDEIAASVNELLRTIHEEVPLYADHHQL.

>Aradu.45U0D.1 genotype-assembly-annot=V14167.a1.M1

MLNSHTPPPPSSTVEFSTHDDDKLLLKTFFSAQDFYSNNNTTFCEVPSSSRRNFSHIYPTINISNNINNHSSPSPSSSSNNMTSHSLDLPAFMTTTTHGGGAAAHQQVDLGLATFSDNHLSFHLDHHHSHHHRPTHASSPPCSNSTTSTTANNNSHPSQYFVSNGTVDTKRPCTSIMDTKASQSLTASKKSRSESRPSCPPFKVRKEKLGDRIAALQQLVAPFGKTDTASVLMEAIGYIKFLQSQVETLSVPYMKPTQNQTNTRMMQGVSAIGGGNGEPKQDLRSRGLCLVPLSCMSYIAGDASNDTWQQSNFGGAT.

>Aradu.959KY.1 genotype-assembly-annot=V14167.a1.M1

MDHNNSDLFQLLGAGGGGGYYPAAAMLMMDHHQHQVSSDDNMSAIETEASHHHQQQDRALAALRNHKEAEKRRRERINSHLARLRTLLPCNSKTDKASLLAKVVQRVKELKQQTSEIITEFETLPSETDEISVLSTADGGHGGLIFKASLCCEDRSDLIPELIEILKSLHLKTLRAEMATLGGRTRNVLVVAADKEHNSIESIHFLQNSLRSLLERSGSADRSKRRRAMDRRFTPASS.

>Aradu.Q74TI.1 genotype-assembly-annot=V14167.a1.M1

MEVGMGRVVWDDDDKATVAAVLGARALDFLVTNAVSNENLLVAVGSDETLQNKLSDLVDRPNLSNFSWNYAIFWQLSQSKSGDWVLGWGDGSCREPNDAEEGGGGGVRGGILSLRIDDEMQQRMRKRVLQKLHTTFGGSDEDNYAFGLDRVTDTEMFFLASMYFSFPRGHGGPGKCFASGKHLWLSDAMKSNSDYCVRSFLAKNAGFQTIILVPTDLGVLELGSVRTLPENFELLQAIKSVFSSQATTTYPMANSKPLMPLTVMSEKRDENNNALFPGLAIGDHTHQNNNKVVEGVPKVFGSAVISGRSHFREKLAVRKMEERPWVPHHQPNGNNNANANANANAINFPNGPPRNGLHHGSAWVGGAAVGGNSNNSNCNNHTQGGVRQAAAGGAAEIFAPRSSACNVSAELANGGVRPQDFRISSYQPQRQVQMQIDFSGATSRPSVRPVVADSELSDVEASCKEEQPSMADERRPRKRGRKPANGREEPLNHVEAERQRREKLNQRFYALRSVVPNISKMDKASLLGDAIAYINELQAKLKMMESERERHGGSTSFREPEAMQRAAENHHGGGNADVDIEAAQDEVIVRVSCPLDSHPVSRVIQTFKDAQINVIDSKLAAANDTIFHTFVIKSQQGCEQLTKDMLIAALSPESNSLQQLSSVGG.

>Aradu.RB7BN.1 genotype-assembly-annot=V14167.a1.M1

MAEKKLELFRAGMNPTSIYANDCSLEQIVVKEGSSSSISSSIITTKKTPSKEKDNNKHEAAAAASQFSYTSLYNSSSSAATTTFKKPRIADSSLLNQQDFAAGKLMNFSAFLRPSMLSQNNSVSREADNNKEEPFPEKQLKSVSNNNKRKVVDNGFCNEPLIEYSSVCSLGASTNNTHIYSRKQDLHDSTYLSEQRRDKINKKLRVLKDLIPNCNKVDKASLLDDAIEYLKALKLHLQSTI.

>Aradu.DP2D5.1 genotype-assembly-annot=V14167.a1.M1

MISTEESWTTWLCDLEEEDYSFINGIIAAPNNYSNSQMSNNDNERPSKLLKSTTPTPRRRRTTGSAGTGRSPQHAHDHIIAERMRREKISQQFIALSALIPGLKKMDKATVLGDAIKYVKQLQEQVKVLETESKRKSAESVVYVEKSEVCGEEDVSVSDTWSNSGGDGNSSYEVSKAVSRSVLPEVEARVSEKNVLIRIHCEKHKGVLMHILKLIDKLHLSVLNTTSLPFGASIVDITITAEMDDKFSLSAKELARNIRVGVLQSM.

>Aradu.GMN2P.1 genotype-assembly-annot=V14167.a1.M1

MDLSFCFSCFLFVCYSISLLLVLVKGIISSILKHFAFGSSKKDFYYHLLPSGDKNMNMMMMQNLIERLRPLVGLNGWDYCVYWKLSEDQRFVEWLGCCCGGTDQNNNVGEDIHIFPCRDTMFSHPRTNHCYLLSQLPTSISIIDSGIHAQTLLTNQPNWVNYSPNILQETIGTQVLIPVPGGLVELFVTKQVPEDHQVIDFVTAQCIVLVEQEAANNSTSFNMQSNVVGDDNNNNHNNNNNENQVMMNNNNNSNNQFVLTTAPETSPHDEIPITLCSSPLNFMQQFRTMNKNNDNNNNNNAFSEEYQGSIFLHENQNNNNQPMKAAAMEEEEEEEQQVGTKENNNNNKNEGGVVGVGRSDSMSDCSDQNEEEEDGKYRRRNGKGNQSKNLVAERKRRKKLNDRLYNLRSLVPRISKLDRASILGDAIEFVKDLQKQVKDLQDELEDNNNNSDTTAVDHHDSSNQFLDHFGASYVVPNHKHMQQDAVDATTLTMDKQHSQQMEPQVEVAVIDGKEYFVKVFCEHRAGGFVKLMEALNTLGMDVVHATVTSHKGLVSNVFKVEVRYHLTHHSKQN.

>Aradu.WS4QW.1 genotype-assembly-annot=V14167.a1.M1

RSEVFSNLINLFSSMFKLLLPHQIPKTLQELLLLEKIMSEKFCVSDEDKGVLESVLGAEAVAYFVSAVSNNFFSSVVASASALAGTDAGLRRRLCQVLEGSKWNYAVFWQVAGLKSGGSALIWGDGQCCDPKGGGAGEGGSEGDWSGVSKGDEEELRKKVLQKLDAYFACSVSKEANYARLDRVSDLHMFYLASMYYIFGFDSPCGPGCSFKSGKSIWVSDSASCSNQLESRSFLGRSAGLQTVVFVPLKAGVVELGSVETVPEDQGVLDLVRTAFGESSSGQAKAFPKIFGRELSVGGGDAKSQSITISFSPKLEDDSGFTSDSYEVQALAANHGYGNPSNGSMGENSEAKLFPQMIGGNYNAQTRVSSLDLGNEDSSSTHLDERKPRKRGRKPANGREEPLNHVEAERQRREKLNQRFYALRAVVPNISKMDKASLLGDAITYITDLQMKIKVLETEKNMNNNNEPMFPFSDIDFQAREDDTAVVRVSCPLDLHPVSRVIKTFQEHQIMAPESGVSTSEGKMIHTFSIRAQGSEAAAIQLKEKLEASLSKN.

>Aradu.G38ML.1 genotype-assembly-annot=V14167.a1.M1

MNLWTDDNSSVMEAFMTSSDLSSLWPPPPPPPQSASSAAVFNQDTLQQRLQALIEGARESWTYAIFWQSSYDYPSSASTAVLGWGDGYYKGDEDKGKAKSTKTTTPAEQDHRKKVLRELNSLISGSAAAPSDDVEEEVTDTEWFFLVSMTQSFVSGTGLPGQAFYHSSPVWLTGPDRLAGSSCERARQGQVFGLQTLVCIPSSNGVVELGSTEMIFQNPDLMNKVRILFNFNSNSIDVGSSWPLTGSTTTTAADQGENDPSSLWLNDSEIRDSVTTVTTVTTTTPASVISVNPGSSALAETPSSVHLPNNNNNTHAGGASQSQNRSFFSRELNFSEFGFDGSNAVKTGNGQHHSLKPESGEILSFGESKRSSYGGGGGGGGGGGNANFFSGQSQFVAAAEDNNGKKRKSPNSRGSNNDDGMLSFTSGVILPPSNMKSSGGGGDSDHSDLEASVVKENESSRVVEPEKRPRKRGRKPANGREEPLNHVEAERQRREKLNQRFYALRAVVPNVSKMDKASLLGDAISYITELKSKLQTLESDKDGLEKQLDSLKKDLDNVKKEASSAPPPLPDKELRMSSNNLVGGGKLIDLDIDVKIIGWDAMIRIQCSKKNHPAARLMAALMELDLDVHHASVSVVNDLMIQQATVKMGSRFYTQEQLRSALTSKVGGDVR.

>Aradu.75YXP.1 genotype-assembly-annot=V14167.a1.M1

MNFFHQDNNNSNSFFHPSSFPSSMSFITNLHYPHQATPKPMFSSLLSGMMIPSNTTNNPLFDQNGGNNNSCQQVMGFLEPQAFATSPSNADATLLGGLGFPTSLNNSSSDPNNNSLSATMSCVIPQEHEEEDKPPNKFALFEGFHNNIENNTGSSAVAASSSSWMMNNNYDDYSDHDMVEDMKMESESSSKNKNIIGENNNKNSNNNLNLQKGNNNKKKTGMPAKNLMAERRRRKKLNDRLYMLRSVVPNISKMDRASILGDAIEYMKELLQKINDLHNELQSMPPTAPASSLHHPLTPTNNNTATLPPAAASLPSRMMMKETASCPTSSLPTPNDQPARVEVGLREGRGVNIHMFCDQKPGLLLSTLTTLDNLGLDIQQAVISFFNGFAMDIFRAEQCNEGQDLHPDQIKAALIEASEASGFHNII.

>Aradu.G27H3.1 genotype-assembly-annot=V14167.a1.M1

MEDMIISPSSSSSLVSIPQENTPTLQQKLHFLLQNQLPEWWVYGIFWHTTNDDNGNLFLSWGDGHFQGAKDASSPRLIPHYPISVTDRCRKFNNNSNNNNDGEWFYMMSLTRTFPMGEALYSSLPGKAFGSGSPIWLNGDLQFCDCDRAKEAYMHGIRTLVHIPTTDGVLEMGSYDLITENWALLQQTKSLFGSQHQQLNPIQFFDDQNISFADIGIIAGVQEEDNSHEEDAVRKRKETEALNNKKKNKDHSSMGKFRSSRQSSFVDSEHSDSDCGAPMAAAEKRLPKKRGRKPGIGRETPLNHVEAERQRREKLNHRFYALRAVVPNVSRMDKASLLSDAVSYINELKAKIEELETQVQKESSMNNNNKKVKVETGDSMDNQSTTTTSSVDQNNNENEAAEVQVEVEVEVKMIGEEAAMVRVQSESGTHPGAKLLNALRELEFQVQHGSMWCVNESSIMLQHVLVKLPEGITMRTEQALKSSILSRLLDHHNQ.

>Aradu.V6ZMF.1 genotype-assembly-annot=V14167.a1.M1

MQPCSREMQSLNSLFNNQQQIQMDPATSQEDDFIKQMLSTIPPPPPPSWNLDHTPPNPKPYWLDNDNVAFPNYDDPSTLASKFRNHQITATSSSKAAAAALLLQQQLLMTRGLSGADSAILHHMFDSSQNDVVDASSSSFKSPNPSSADGSVQALYDGFTGSLQGAALASNQTHHFQHPQVQTSSNPNQMQGQGQGTSQGGNPNPNPGQPKQRVRARRGQATDPHSIAERLRRERIAERMKALQELVPNANKTDKASMLDEIIDYVKFLQLQVKVLSMSRLGGAAAVAPLVADINSEGGGDCVQTNGNQTAGASSSNDSLTMTEQQVAKLMEEDMGSAMQYLQGKGLCLMPISLATAISTATATCHPRNPIMNPTDGPSSPGMSVLTVQSANGDAVKDATSVSKP.

>Aradu.L9W8G.1 genotype-assembly-annot=V14167.a1.M1

MVLCSQLSVVLDHTFYKCGRAHMALHDIDAAVESFKNALVLEPNDERILGNPNQSLKLKRQETENSECHSEVNDIEEESVGVKKACPTRGVKRSRSAEVHNLSERRRRDRINEKMRALQDLIPNCNKVRLFILSNLEPYLLLLEPKMQPEKQRNETKRQEAWQVYGALLCAVGQCMHEKAKIFSNLLSPPTRASSRPKAMIAMSSMTSLCYNEIYTGVSK.

>Aradu.6L1EK.1 genotype-assembly-annot=V14167.a1.M1

MQLEMSEDIRVGSPNDGSNNLDSNFHLLAVTEADNQSGQVDSYRVDPTQRWDPIQSPLDQLQVQLPVFSPIEDQILTQEDDHYSQTVSSILQNQSTRWVDSPSIACYVTCSNQSAFAKWTSLVADEHLHAATVDGSSQWLLKYILFTVPYLHSNKNNEENNSSPNNTNPTSSAGPSDRLRGGGKGSGTPQDELSANHVLAERRRREKLNERFIILRSLVPFVTKMDKASILGDTIEYVKQLRRKIQDLEAHIRQMEAEQQRSRTPSTTIEVHHCGSSNKEQQQHTVVVGHEKRKVRIVEGTKTATKVVAAAATEASVQVSIIESDALLELECSHREGLLLDVMLVLREMKIEVIGVQSSLNNGVFVAELRAKVKDNNSGKKVSIVEVKRALNQVIPHTGD.

>Aradu.S0KU9.1 genotype-assembly-annot=V14167.a1.M1

MMEIASSNYLAEFGIEEYSSSFQEYPMMMNSFEEMLDKFEMDMQSMSSAYSETKPPPHQLQSPFNTAMPSRSASPSPPKLISFEAPSLPNSSNIKNPNLMMDDHIHFSAFFNHDNPPHKVLPATARNPIQAQEHVIAERKRREKLSQRFVALSAMVPGLKKMDKASILGDAIKYVKQLQERVQFLEEEKARKKTMVESGVAVKRCFVFVEDEDNNENEISAAAALLDGNCNTLPEIKARVSGKDVLIRIHCHKQECKNSRGAREAAILSVLEKHNLTVHTTTSLPFGNDTLDITILAQMKKECSIRTKDLVGSLRVALTQFS.

>Aradu.UV8L7.1 genotype-assembly-annot=V14167.a1.M1

MLQQNLQFLLQSQPSWWVYAIFWSTTKDDNGNLYLAWGEGHFQGNTTEHTKTQQQHNQNDAEWFYVMSLTRTFSIANSSSSSLPGKAFALGSVLWLNSKEELQFYNCERAKEAHAHGIETLICIPTSNGVVEMGSYNTIPQNWNLINHVKSVFEDSMVNNNNNNNNNNHCNNNNLHNTVQQIFEDNDDFSFAEISFMAGLEEGREQVISRKQADVTVAFNKESKDSYAESEHSDSDCPFLKTENTENKNKSEAPKSKRGRKPVLNRETPVNHVEAERQRREKLNHRFYALRAVVPNVSRMDKASLLSDAVAYINELKAKIKDLESEKDNYQRKKKVKLETGDTMDNQSTVTNSSTVVDQEKCSNGVVAEVDVKIIGDDAMVRVQSENVNHPGARLMGVLRDMEFQVHHASMTCVNELMLQDVVGKVPNGIFTSEEGIRSAILMRLDH.

>Aradu.WUW36.1 genotype-assembly-annot=V14167.a1.M1

MGSSENPNWVLDYAYLDDIHSLSDPSNFSWPPPPTSLSADLHQSLPNSHGPDQSASRKRLRSSDSKACREKMRRDRLNDRFLELGSILDPGRPPKTDKAAILSDAVQLVSQLRGEAQKLKESTENLQEKINELKAEKNELRDEKQRLKAEKDSLEKQLKALNTPSGFLHHPPTLPAVFPHAPGQVLGSKLVPFMGYPGVSMWQFLPPAAVDTSQDHVLRPPVA.

>Aradu.5Q1VY.1 genotype-assembly-annot=V14167.a1.M1

MEELNGLATNWLSDLDIDDYELFSECNLKKFLDEDEENQFDEMVMSAVVGEEEEEEEERPTKQLRTCSSSSITNYVSSNSSSSSSPTSQILSFENSYNPSSQFYGFEFDATPNDKVSPQPQVGNNNDKNKLEQTRKPQSQGSKRQAAHSHDHIMAERKRREKLSQSLIALAALIPGLKKMDKASVLGDAIKYVKELQGRLKVLEEENKREVESVVIVKNPRFITSSSDDDSSSCDDDTLEADGEAALAHVEARVAAAEKEVLLRIHCKKQKGIYVKLLSEIQSLHLYVVHSSVLPFGDSVLDITIVAQMGTEYKLSIRDLVRNLRVATLKTMSSS.

>Aradu.QW16A.1 genotype-assembly-annot=V14167.a1.M1

MLTENFKEQLALSVRSIQWSYAIFWSTSSTQPGVLSWGEGYYNGDIKTRKTSQGVELNSDQIGLQRSEQLRELYKSLKTVEASPQTKRPSAALSPEDLTDTEWYYLVCMSFVFNIGQGLPGKALAKGEPIWLCDAPSSDCRDFSRCLLAKTVVCFPFMDGVIELGTTDYAKDLSLIQQIRTSFLDILDVGVHNVHGAFNAKQSQEVGGALISITSPNNSSNAFQANQPPDETFMVERINNGTSQVQSWQIMDDELSNAVHNSSDCVSQTLHSPENVASLPKGENLTDSAKDLQKCNNSKMTLVDPRSDDWHYQMVLSTLLKSSDQLIMGMHFQNFHQESSFTSWKKAGSVSYQRPRTGGSSQALLKKVLFEVPRMHLDGILEYQEENDFKEAVRTEADENGMNHVLSERRRRAKLNERFLTLRSMVPSITKDDKVSILDDAIEYLRKLEKRIKELEAHKDLTDREARTKRSPQDMVERTSDNYFNKTDNGNKSMAKKRKSRDIDDTRPEINSESLLKGSSTNDVAVNINENNEVLTGPTIVSAKRIKQTLQNVAFKC.

>Aradu.DRR9K.1 genotype-assembly-annot=V14167.a1.M1

MMSSSWASMEAETEHQQQTHNDRDFTSLCSFNNNLPFMEVDDVNDIMMMMTMMRDMSNFFPEPDTTNNNNLVLLHHHSSSSSSSSSSSSHLQCFLPPPPPPNPFDMPTMASFLDPHQASITATTDFGGFHHHNLEELGSCSSSGKPAPPPPPPLFFNRSKILRPLDDSLPPSGAQPTLFQKRAALRKNNSSSQQQQGGVLGEDDNNNNKNSMKRKVMMEMEDNGSFDGSSAFNYDSGDDLIETNNKKNNQKGGGGKNKKGMPAKNLMAERRRRKKLNDRLYMLRSVVPKISKMDRASILGDAIEYLRELLQRINELHNELESSSSTSTNSGSGSVMTAVPTVVASSSFYPLTPTPPSLPTRIKEEICPTSLPSPNAQPPRVDVRLREGRAVNIHMFCGRKAGLLLSTMRALDTLGLDIQQAVISCFNGFALDIFRAEQCKEGQDVHPEQIKAVLLDTAGYPAII.

>Aradu.YKL3P.1 genotype-assembly-annot=V14167.a1.M1

MAFSQPLIGSADMTSATEFPPATRLLPAGGTDDDAVALGAGFEMEAKWGEGEVEEGSIEASVWNYDSDENVESGSKAVVVEESGNHSGGGNGNLSLSGGVNGGDQKGKKKGMPAKNLMAERRRRKKLNDRLYMLRSVVPKISKMDRASILGDAIDYLRELLQRINDLHNELESTPPGSSLQSSTTSLQPLTPTPQTLPCRVKEELYPGVLPSPKNQPAKVEVRVREGRAVNIHMFCTRRPGLLLSTMRALDNLGFNGFALDVFRAEQCREGQDVLPEEIKAVLLDSAGIHDMM.

>Aradu.K7LHY.1 genotype-assembly-annot=V14167.a1.M1

MHEQPGCFDPNTMAEGVSTPKLKDTFPQTLSDPSSSPSPLIVVGNTTNSNNNLEENIRLSMEELSYHHQQEDVSNYVNGVTATTIDIPHPQHLGLNMGNSYNNNINMDSHLVQHEIDILPYQQPTWDPNVQEMQDMGYTNHSEHQPHDQQFQQTEAQNCSQSYNPSSILDPPYPSQDLLNLLHLPRCSTSSLLANPAICIANKTQNFQNPMGFLGDLPIGSDNTSASSVLYDPLLHLNLPPQPPALRELFQSLPRGYSLPTNSRSGSLFGGGDEIEGDGSQLDMGVLDFNRVTASVGKGREGKGTKHFATEKQRREQLNGKYKILRSLIPSPTKMDRASVVGDAIEYIRELLRTVNELKLLVEKKRYGRERCKRQKAEDDAAESCNIKPFSDPDGCIRTSWLQRKSKDSEVDVRIVDDDVTIKLFQRKKINCLLSVAKVLDELQLELHHVAGGHVGEYCSFLFNSKIIEGSSVYASAIANRVIDVMDTQYAAAVPHTSSY.

>Aradu.T3S5X.1 genotype-assembly-annot=V14167.a1.M1

MSVMYSPVLKYSHGVLRKGHEMISNINHHQHQDQNHHHQQQQQQNSRLLRYRSAPSSFLENLVDINNGGASVNEDPPLGHGCYHHIPSTSSSSEAMFSKPIQSNNGWSRRDSEPVQVQEEYGGSNNKPTVLKEENGNSSFLKEQNGYPYWTQQIQSLPTNNNGYDGSFGVVNSVNSDHSTQSEMGVRSCSNLIRQKSSPAGFFSSENALREVERFRSNDVSSNRSSSCLKRMPQIAEYENESHEGNLVNDNGSSKYYVPSFTGEVWDASSFNSQKAASADEIMFSASTALESQDSDFCSQNLGLIHHLSLLSSSTKMGSMEKFLQLQGTVPCKIRAKRGFATHPRSIAERVRRTRISERIKKLQGLFPKSDKQTSTAEMLDLAVEHIKELQEQVKMLTNIRSKCRCTNTQKQYS.

>Aradu.83N8C.1 genotype-assembly-annot=V14167.a1.M1

MMQISSTNKYLPEFGMDESVFFHNQYPMMDSSTITSSPSPWPQILDDIDFNQIQESFSSASSPKSYTSNKRFNSSSAAGTTMPQSSFSPIERPTKQQKNAYSHGISTNTTTTTSNEFMHSNASSSVASYNHRHQQLYNNNPSSDQGNHVLRPKTESVCSENLDFATVVSHADYLSGYDKANKGSAAAATTRNPTQAQDHVIAERKRREKLSQRFIALSAIVPGLKKMDKASVLGDAIKYLKQLQEKVKILEEQVAEKTVESAIFVKRSILFAEDNGSSSEENPDQKIPEIEARISGKDVLIRIHCDKHSGIVPKIINEIEKHDLSVQSSSFLPFGNNSLDITIVAQRI.

>Aradu.023N4.1 genotype-assembly-annot=V14167.a1.M1

MMEGVFFLPEVARTGYLRSIVQSVGCAYICLWSFDPTSSPNNRLFFLDGFYNNVRNNQQASSSLGSVAQQLFNQFRTLRFDANDDRIPGLAFRNNRPYVEVQQPELLRLAWTQIQKQFFQEARIKTAVFMGCNKGEIELGFLNLSQAEIQTALSSLFQEDFSSGRQIMDHQNNPPSSSSSSLRSLSTTAGSPEYSSLLFNIPPAGATAGAIVPNTMSPLSSNTQSALLTNYVFPSHHQEIENETLMRVFLNAISPQQHQNLPYNITVVHPESSAFKRYRTEPEPGPERAPESLRSRQSLMKRSLAFFRSINAMRIRERIQATRPSSTQLHHMIAERRRREKLNDNFQALRALLPPGTKKDKASILTTAKETLSSLMAEIEKLRIRNHELESRLPESSKESSAADQEISKTMLLVPPNERFHVQISDVPQSSSSSSSEERRVDLHVALRGQISQIDAVIRLLEFLKLAQNVSLITMRTNTNNVGQGNNNYINQLTFRLRILEVCYLSFIKMHVLIVTC.

>Aradu.DSN52.1 genotype-assembly-annot=V14167.a1.M1

MENIGDEYHRYWETSMFLQTQELDNWGLDEALSGYYDSSSPDGAASSAASKNIVSERNRRKKLNERLFALRSVVPNISKMDKASIIKDAIEYIQKLQEQERIIQSEIVELESGMMVEKKNPNSYEFEHQELPVLLRSKKKRTDELYDSLNSRNSPIHILELRVTYMGEKTIVVSLTCSKRTDTMADEEEKDLLQIKIQTAIAALNDPLSPMSI.

>Aradu.MB6LX.1 genotype-assembly-annot=V14167.a1.M1

MDAHQETLIFNNDYEVHDFTEDPNFDQLINLIRGDNNNEDDAAVFFNYGSDIITNDCFLDDNQNQNHLLLPYDQIHQSHNNNSSNEVMMNNVCGDDPSPTNLISSFSCFDEGVVNRENEGEYSSATTTATDEANLSGKPRLKADRSKTLISERRRRSRMKEKLYALRSLVPNITKMDKASIIGDALSYVHELQAQAKKLKAEVAGLEASLFVSENYKASFDDNYIKTVQVTHNSHPINKKIVQIEMVQVEERGYYVKIVCNKGGGVAASLYRALESLVGFSVRNSNFETVCDTILLTFTLNVKGFEPEVNLPNLKLWVTGALLNQGFEFMANFHY.

>Aradu.PBW5F.1 genotype-assembly-annot=V14167.a1.M1

MAEDQFQASGNWWENPAIRNNWQPHHQQQDESDHMMKPTRVSMDSSGGAGGGSSSVVFHDPQKLQPPDSATAATSSTDPNLHFMALGLSSPPIDWNQQPSFLRGEKGGENSFRSMLQEEGGGGGGMGLSPQQVHQWRSPETEFKNNRGFCLEQNQFSPQYSSGDSTVTSQQGFHHQNMDHSAVLYGSPSSILQGLLGPDHHHHHHQQQANHNFSYPTSNYGLSSSSSNDFVPSSSSSNWSNNNNNNNNNNKVPQFLRGSPPKQLSNSSSSPITTTNTSLHFTNNAPFWNAAAAAASDPKDVRSTTFFPNSLQPPFFTNPSFDVQSKNMSEMRDSGAVVKKSGSEPTPKRPRNETTPSPLPAFKTDTASVLSEAIEYIKFLHEQVTVLSTPYMKSGAPIQIQQGSGKSKEGEGPKQDLRSRGLCLVPVSSTFPMTHETTVDFWTPTFGGGSR.

>Aradu.GH2K1.1 genotype-assembly-annot=V14167.a1.M1

MAHQQRGQASSSTKMERKIIERNRREQMKNLCFKLNSLLPNFNPKQTLPPRVDQIDEAIIYIKTLESRVEIAKEKKESLLNKGMKKRPHDLAGSSAFDDETQGTSLKPPTIEIHERGSMLEIVLMISGFDNQFIFSEIIRILHEENIEVLSAISSRAGDSMIHVVHAENHLYQLGATKEISERLKRFVDAHRLQDIQGAQGP.

>Aradu.Q8Q5Z.1 genotype-assembly-annot=V14167.a1.M1

MVMKILGKCAFDYLASNSFITNESMLMATGSFENLQNKLSDLVERPNLNNFSWNYAIFWQFSQSKFKKDCVVLGWGDGCCREPIEGEEEREALRLGFDDDEVVQRMRKRVLQKLHTVFNGSEEENENYAFGLDRVTDTEMFFLASMYFSFPKGYGGPGKCFELAGIKTVVLVPTELGVVELGSVRIVDENLDLLKAVYSVFSSSFAHSSSFNDVVNVKGDEKNRFFCGLDVGNWDRDRDIRVRDHRVEGVPVPKIFGKELNVGNLNSGGKNFREKVVVRKIEEKKSWNGYPNGNNGIRFLNNARDGVNGSRSWEVNQGLSQGCLGGVFPPIPRSCPRSQSFGRSIVGECEISNRDSSHKEEKPEEKPSTTQEKRPKKRGRKPANGREEPLNHVEAERQRREKLNQRFYALRAVVPNISKMDKASLLGDAIVHINELQAKLKALEYEKSTLESTSKASLPGIEANLRDRTCSFKVDIEADQDGVIVKVSCPIDLHPTSKLIQALKDSKMSILESKFNATNDHVFHTFVIKSHASEQLTKEKVIAAISGESNSRKLIFA.

>Aradu.T39L1.1 genotype-assembly-annot=V14167.a1.M1

MDSNNINSGLLRYRSAPSSILTSLMDNIHHGGGGGGNEEALRTELEKLISSNNKSKHVNNSSEHLKREEGNNNYNYSFGSQNHVIYQNNQHYHQIHQGLAMASSCNNGFDGTFFGATNSVDSENNNDNGTQHKMGSNLIRQKSSPAGFFSNYSIDNNGTTTSSRSNSGLQCTLNFSSRSSSCSKSRMPQIVESGNEGMESNCVENRNLIIRSDDSNNNNNNNGSTKCYMPRFTGDVWDASEFNSSKRGTNNGEIMFSTSNAMEAQDVDFGYQKVGLTHHLSLPSSSTKIGGMEKLFQIQGSAPCKIRAKRGFATHPRSIAERQTSTADMLDLAVDYIKGLQKQVKILTDTRAKCNCSSNYQKEQ.

>Aradu.53P8J.1 genotype-assembly-annot=V14167.a1.M1

MILSLRVLLISSHTNTCCFFSLPLTMDIDSSGGTSNWLYDYGFDNISVADFMAPDSAAFTWLPQPQPHTNFKPPSSNISLEMEYSVDSIVLEGGPSKRLRTESCASGTKACREKLRRDKLNERHAIILNLFLELSSILEPGSMQPKTDKVAILSDAVRVVNQLRDEAEKLKEMNDEMLEKIKELKAEKNELRDEKNRLKVEKEKLEQQFKMTNVQPSFLHHAPAATKAHGASQKLIPFIGYPGIAMWQFMPPAAVDTSKDHLLRPPVA.

>Aradu.N3W47.1 genotype-assembly-annot=V14167.a1.M1

MDNTIDQLKSSSSSEEDQMDLMNMMMMQLPNEFSSPNYADETSVQGFHPSPQLLGARSSSTMQLSDSIDNRNSIMPWSSPSTTISFSNNNTTSMLQTELPSLLGNSGATENKRNSMAAMREMIFRIAAMQPIHIDPESVKPPKRRNVKISKDPQSVAARHRRERISERIRILQRLVPGGTKMDTASMLDEAIHYVKFLKAQIHSLERASSSASNNNNNNATASGAGAATATANAPGASYLSLPKPYQGRNAHHYGDA.

>Aradu.VUX24.1 genotype-assembly-annot=V14167.a1.M1

MDDNNLSDIFQDKDFGGDDLFAILESLDDFPPTNTGQFSTLLESSSLPSPPTKRRKLLVPPPPPSPNAAEDGQQRMSHITVERNRRKQMNEHLSVLRSLMPCFYVKRGDQASIIGGVVDYINELQQVLQALEAKKQRKVYTDHVLSPRLVSSPRTSLSLPLSPRKPPLSPRLSLPISPRTPQPGSPYRPPAPCRLLQPAATAAAAASSYISPAASISNSLEPSPTTSSASSINDDINELVANSKSAMAEVEVKFSGPHLLLKTVSQRIPGQALKIISALEDLALEILQVNISTADETMLNSFTIKIGIECQLSADELAQQIQQTFC.

>Aradu.X6KLL.1 genotype-assembly-annot=V14167.a1.M1

MALEAVVFPQDPLFSSYASSTTTTKDYFYSSFLGSHHHDLIGIINNNIIQQNQATNNSDSSSPPSNNDHYATSSSSPDCCAATVDQPSLPTTTGRRKRRRTKTTKNKEDIENQRMTHIAVERNRRKQMNDYLAVLRSLMPPSYVQRGDQASIIGGAINFVKELEQLLQSMEGQKKPNHHDTTAAGGAAGMNSSGQSSPFGEFFAFPQYTTRGHHQGKTMAAEQQQKQWAAAVADIEVTMVDSHANLKILSKKRHGQLMKMVVGLQSLSLTILHLNVTTLHDFVLYSVSVKAEDGCQLNTVDEIAAAVNQLLCAVQQEASSFNQIN.

>Aradu.10L0H.1 genotype-assembly-annot=V14167.a1.M1

MMAGNPNNWWSMNPSSLIPYTQYVLGSSSSSIPFNPLVAEIPEPSPQSWSQLLFTGLPGEEERLGFNHFHQSKKLENWDDHNILINNNNNQHNTRVGPNNNNMIVDGIIKQEVSQSGNLYGQEEFHASNGSTWSTHMVPINSSSPRSSVTTNNNNNNNLLDFSYNKVDHSKNQLPYATPQCNSTSSAGICKKAKGQSTSSLPPLKVRKEKLGDRITALHQLVSPFGKTDTASVLLEAIGYIRFLQSQIEALSSPYLGNGSKNMRSQQYVHGERNSVFPEDPGQLLNDTGLKRKAASSQDSKDNKAKDLRSRGLCLVPVSFTQHVGNENGADFWAPAYGSGF.

>Aradu.L8VRB.1 genotype-assembly-annot=V14167.a1.M1

MALKAVVYPQSQDPSFGYGVNYNNCYRNWSNSYNNEDQRVETTYPAEIWNNNNTTCSSPPSVLPSSCRPKRRRAKSRKNKEQIENQRMTHIAVERNRRKQMNHYLSLLRSLMPDSYVQRGDQASIIGGAINFVKELEQKLQFLGATYQEGEDGNKKKKKNMAFCEFFTFPQYRRTATATATTSSSSSNSEKSSSSRTVAGEEELGEVAADIEVTMVESHANVKIRSKKKPKQLLKMVSALHNMRLTILHLNVEDDCKLGSVDDIAAAVYQLMNSIQQEAPIILASTNFKIQASSPVSFITKFGS.

>Aradu.XEX7M.1 genotype-assembly-annot=V14167.a1.M1

MEAVGGSGNALFSGKGNGLEALVVSNLKRVQEVEEASNEGSMWNSEEDDERTDEVRRRRKKLNDRLYMLRSVVPNISKMDRVSILGDAIDYMKELQQRVNDLQNELESTPTSSSPSSSTSIQPLTPTLQTLPCRVKEELCPVPIPGNQTAKVEVKVRDGKAVNIHMFCSRRPGLLVSTMQALDNLGLDVQQAVISCFNGFALDIFKAEQCREGQDVLPEEIKAGLLDSAGIHDMM.

>Aradu.065GY.1 genotype-assembly-annot=V14167.a1.M1

MWWKLSCNHFSYFIQLVDISVSTSFTNASNHSTLTVDSSRRLLDPSSNNNHFLPEHADDNHLWNHVLSAVGSNGELQNNEEMGEMKFLDALSSKNMTSSMFEEPACDYLKKLDTTTSWEYSGGSTSSSFNNSFEKHLNNNNNNNNGFSDDALIENNERLTKLSNLVSTWSIAPPDPEVSSSHFDHPPTNINNHHHHDPNCNFKQQVFIGDSTSYPPISSYDHPTKVKEEFHHHHQTNSGFQNGLINGLSSVMGGGDSSGNKFYHDLPNLSSCTRNISDVISFNSRLGRPVLGIHAQKPNIKYINNLSESKKHQGLQTTSPINRSNNGRIEGTTAREVKKKRSEESSEAILKKPKQDTSTASSTKVQAPKVKLGDKITALQQIVSPFGKTDTASVLFEAIGYIKFLQEQVQLLSNPYLKSNSHKDPWGSLDRKDHHKEDAKLDLRTRGLCLVPTSCTPLIYRENSGPDYWTPAYRGCLYR.

>Aradu.GY22L.1 genotype-assembly-annot=V14167.a1.M1

MEQCWENWPLHTEIEHHDHGDFFLSLEQCQKPPDDDDDFLREILQMPPDFIDNHSTVDLVTTGTAESAEGKRPRTFILSFDNSTIIPAMAEQQQPLGVGAGAATTTPLPSLSKKRNLLQKPQQARIPVPVPVPQPQPGAAKRSRNDSQIVDHIMAERKRRQQLTQMFIALSATIPGLKKTDKASILGEAINYVKQLQERVRELEKRNNDNKRGPTEPVIFLNKTQLLCRNNEDSTSEEEEEEEEEEEVEDWRSKEEKQVLPDVEARMLEKEKEVLIEIHCEKENGIEVKILEQLENLHLSVTGSSVLPFGNSTLGITIIAKMGDAYTMTLHDLLTNLRQLLLINNTTDPY.

>Aradu.394BE.1 genotype-assembly-annot=V14167.a1.M1

MLGSINDETFSPKLSSSSSILSLRSQILSFDNSDSLSSSPSNNTTEFYELKNTLNQRQDSVETPNNNNKKSDSLNTQNVEAKSTSTLGKRSPSHAHDHIIAERKRREKISQSFIALAALVPGLKKMDKASVLGDSIRYVKELKERLAVLEEESKKTKAKKAEQLHGHGAAVFSSLCEETIDGSLPQVEAREWGQQVLLRIHCWKEEGILVRILSEIQSLQLMLLNSSVLSFGDSILDITIIVQAGEGYNLTLNELVKNLRTATLKLMS.

>Aradu.YPV42.1 genotype-assembly-annot=V14167.a1.M1

MASRESTNWLFDYALIDDIPVHDATFAAPSSGFNWPSNAINGSSNVGVEIDGSLGDSDGIKESGSKKRGRSESCAPSSSKACREKLRRDRLNDKFVELGSILEPGRPPKTDKAAILIDAVRMVTQLRGEAQKLKDSNMGLQEKIKELKSEKNELRDEKQRLKAEKEKLEQQLKSMSAQPSFMAPPTAIPAAFAPQGQVPGNKLVPIISYPGVAMWQFMPPAAVDTSQDHVLRPPVA.

>Aradu.F6ABZ.1 genotype-assembly-annot=V14167.a1.M1

MEAKVDFEVDNLTTATGSSSFSQLLFGDDDDHHQHALGFAATVDHLPLFSIDKPPKMLSFGDFHHLQEQQQQPHLLLPETNATPQKSVITSSDSSSASSCNHTTTAINSMSKTNKKRNWPGLQPIVKVPNSNNKKNKTENPPSSTSSGHAKKKEKLGERIAALQQLVSPFGKTDTASVLHEAMGYIRFLHDQVQGEEEGVKEDKRDLKSMGLCLIPLQSTLHVSSTNGADFWSPAGAANNVTPYAK.

>Aradu.T4PBI.1 genotype-assembly-annot=V14167.a1.M1

MEIFYDDHHDFLEELMALRRETFMETNYNNTTTFHSSFPPNSSSSSYQQEDLILPHQIYDDNNFFNQIYDSLLFDEQSPPPLMMDHYSSSSYYSNNTSSNLDSYYPPLSFMEEEELQNLEIETATRCKVEPVVTVTCCDSESPAAIFNADSVMGNKEIRKNNRSTKKVVGQPSKNLMAERRRRKRLNDRLSMLRAIVPKISKMDRTSILGDTIDYMKELLEKINNLQQEAVKVEDSNMAGGIFKDVIKPNEILIRNSPKFEVERDVDNTRVEICCAGKPGMLLSTVNTLEALGLDIQQCVISCFNDFTMHVSCSEEPEQRAMLSSEDIKQALFRSAGYGGRCL.

>Aradu.B7RDX.1 genotype-assembly-annot=V14167.a1.M1

MEDCEDYKNYWETNMFLQTQELDSWQLDEAFSGGYYDSSSPDGGASSAASNKNIVSERNRRKKLNERLFALRAVVPNITKMDKASIIKDAIDYIEHLHEQEKRIQDEIMELESEKLKNPIIAAAGDYDFEQDLPVLLRSKKKRPDNLLFDTSASSSSRNNINYFPIELIDLRVTYKGEKIFVVSLTCSKRTDTMVKLCEVFESLKLKIITANITSFSGTLSETLFVEADEEEKDHLQIRIQTAIAALNDPQSPMSI.

>Aradu.RL2B3.1 genotype-assembly-annot=V14167.a1.M1

MGSENQTPMTETSKNRSSGKTNQGKVPKRIHKAEREKMKREHLNELFLDLANVLDLNEQNNGKASILNETARLLKDLLCQIESLKKENSSLLSESHYVTMEKNELKEETSSLETQIEKLQVEIQARVTQSKPDLNAPPHVEIEQSEQANFTGQSLQFPPMEPNLQQGPAVFVVPYRPGFQAAFSAPNVAEVTPKPSSAISKPHARYPTPADSWPSLLLGQQPTSS

>Aradu.5FE4Y.1 genotype-assembly-annot=V14167.a1.M1

MMELPQARSFGTEGRKPTHDFLSLYSNSTSQQDPRPPSQGSYLKTHDFLRVETKANTKEEASDETSSTVQKLPPAAPQPPPSVENHQHLLPGGIGTYSISHISYFNNNNNNNNNNNNNNNNNNNNNNNQRVPKPEAPLFTVRQATSTDRNDENSNCSSYTSSGFTLWDESAVKKGKTGKENNIVGDKLIPGETQSAKQGHWTSTEGTPQSFSNNRHNSFNSRSSSQTTGQRNQSFIEMMKSAKDCAQDEELDNEETYFLKKESSNPERAELRVKVDGKSTDQKPNTPRSKHSATEQRRRSKINDRRVLPILHKIFQMLRELIPHSDQKRDKASFLLEVIEYIHFLQEKVHKYEGSFQGWNHEPEKLMPWRNNDKPAESFEACGTNSGSGPSPTLLFASKIDEKNITLSPPITGSSQKLEPGLNTATPFKNMDHPPGITNTTFPIPISSPPNFFPPRKSIGLGEMPHVTHRLPSDAGNGIYQPSVECQTVTATNEKLKEKELAIEGGAISISSVYSKGLLDTLTHALQSSGVDLSQASISVQIELRKQENIIRPTLPMSMCGIKGDEVPSNNQRMMRSRVATSGKSDQPVKKLKSCKT.

>Aradu.6VX3K.1 genotype-assembly-annot=V14167.a1.M1

MASSWSPKHEKMQKSLSTQLAVAVRSVQWSYGIFWAPSTTQEGVLEWREGYYNGDIKTMKTVQTTMEIKNDKIGVQRSEQLKELYKFLLVGESDPQTKRPSAALSPEDLSDSEWYYLVCMSFVFYPNQSLPGRALEIGETIWLCNAQYADSKVFSRSLLAKSASIQQKEILSDHYIMAVTKISMFILCFKPVFQTVVCFPYLGGVIEIGTTELVPEDPNLIQHVKACLLEFSKPICSDKSSSALNKPDDDKLQPCSNGEHVLVDTKVLEYSCSPAEEIKLDQDPIKELQEDSNMDSPDGCSNGCEHNCRVGESMVEAINNGGPSSQVHFMDDDDALSNGAPDSLSSCDCLSEASENRGKASKDVQLIRGIQSFNHLNRSSLDAAASDEHLSYTRILSALLGRSSAFKQNPYASNSNCKSSFVKWKKGGICEQNRLRLEQSLLKKTLFTVPLMHRSFSSIKSMKENETKEWTNRLENADDKFRENVLSDKIRETGNFKILKSSIPHPISEVEKISILGDTIKYLKELETRVEELESYMDMADSEARTKRKCPDMLEQISDNYEARKVYNKGMRKPWMMNKRKACDIDEKNIEEIDRFVVSKEDNNKPLDVKVNMKEQEVLIEMQCPYREYILHDIMDAINNLHLDAYSVESSTTEGVLTLTLKSKFRGTATAPLGMIKEALWKVSGNL.

>Aradu.1QN19.1 genotype-assembly-annot=V14167.a1.M1

MPFLQMLQSVEYSPNYSSQQQCFFPFKDPTNFQTLLRLQHLKNHNNNNNNNNNNVQELDQKSCVTHDVMVEMQQQQQQSHSPVKSESYELHQQHHQTSASASCCVENNNNNTTPTKAVTAAIEKCGGRGNSNTQEVCQKSQQVGSVATTTTRERKKRKRTRPAKNKEDVENQRMTHIAVERNRRRQMNDHLSGDQASIIGGAIDFVKELEQLLQSLEAQKRMRRKNNNNSSNKNEDAIGFGSSSNSSSTTNGGYDGIMRSSTTSLSLLSTEEGNFGGGGGGNGDELKAENKSESAEIEVILIQTHVNLKIQCKRRHGQLVKAIVALEDLRLSILHLNITSSSDDSVLYSLNLKIEEGCRLRSANEIAEAVHHIFNFING.

>Aradu.K087N.1 genotype-assembly-annot=V14167.a1.M1

MDSNSLQGYQLEQQQSSSSGLLRYRSAPSSLLANLTPTFTESQTLLSRFANSNSSSSKNNISKTDTVSHSFQEFPDTNTNTKPTNKGCDSKGLRRMNSGGHGYGGGGLPPHYPRNTSSSSCSSSMDGFLGLVMGSMGMDQKGFGVSSSLNLLRQNSSPADLFSNVSFQNGFATMKGVGNYGHVNSTSEELSPSMNRLKNQISFSSRTASSLGMLSQISEIGSEEIGANSPDDGRQGGSNGDALHYSPGFSYGSWNDAPQQSENLTGLKRGRSSNGKLFPDVQNGELGNQVHMLSHHLSLPKTSSEMIGMENLLQFPDSVPCKIRAKRGCATHPRSIAERVRRTRISERMRKLQELVPNMDKQTNTADMLDLAVDYIKDLQKQFKNLSEKRANCKCISMRRADTNQIV.

>Aradu.Y3AAH.1 genotype-assembly-annot=V14167.a1.M1

MNQLGSRSQASPSSTNKKKIERRVIEKNRRNHMKMLYSKLNSLLPRPKEPLPLPDQVNKAINYIKSLEEKVKMAKEKRERSLGIGIRRKRTRGDCSGVDRQKPPQLEVHEIGSCVEIEMTCGLDTQFIFYEIIRILNEEKIDVKSVNSSLIENFMYHVVRAEGSITLNIPLAENVLMIGVKNQKDPRDGLHHGGWMMKIICWFLLILPSLLQFGVTKVSERLKRFVNEPTSEIELQSDYQLLDFEINTDELAELLGGH.

>Aradu.JT48R.1 genotype-assembly-annot=V14167.a1.M1

MADDQFRASANWWDSSRNVVRFESREPQSGSSGLTNNTGNFSWHGVGDDKIMKLPRSSTMENSMSSSSAGASSGRSCVVFHERFQQQQNLDMMMGLGLSSQASTDWNQASFMRAEKASEMLQQNLSAHQEIQFSPQYSNITSQQVLSSNFQMDSSSSAPLYCNTSMLQGLLGPESNNQPHQGPMSNFPYSTCLNSNNNNNQLHFTNNAPFWNASEPAAVSIKDGRSIFFPSLHQPFSAPKNICEVRESGGSMLKKSENEQSSKRPRNETPPSPLPAFKVRKEKMGDRITALQQLVSPFGKTDTASVLSEATEYIKFLHEQVTVLSTPYMKSGAQTQHHQNSGKSKEADGPKQDLRSRGLCLVPISSTFPVTHEPTVDFWTPTFGGT

>Aradu.9C8PR.1 genotype-assembly-annot=V14167.a1.M1

MAEEFQATICGDNYWWNSNKSLFSLMGGSMTCSVAAANDSANNYDSFWQGSDFMDFKGATTKSSFKGEDTNKCSNNESSTSASAVAGGTVGSIFINSTSQMMSDGLSSSPSFTWNNPSLFGCNERSENNFQSIVIQEETGLDPSNNNSQQIQQQQEFSNSMGSGFTIESSLSASYDYPLMQAGLFGSTDSSSSLFTTNQPTLPELSPIWSTVSSFMKPSSFMEPKQLNGLQFSNNTPFCNASTEETLYSIRSDIFSSSSSQPQLYQTPPTTFDNSKPNNALTSTPLNKLKREESPEKASSSVCSKNVSGRVFKKTRIETPSPLPTFKVRKEKLGDRITALQQLVSPFGKTDTASVLHEAIEYIKFLHDQVNVLTAPYMKNGSSIQDQQGCDYEKDSKCLTQDLNSRGLCLVPVASTFPMATETTADFWTPTFGNSLIGNVAVAGSHQ.

>Aradu.U4ABC.1 genotype-assembly-annot=V14167.a1.M1

KFLTITLSLSPPTHIDTLSSLSLTMAMSVENQDPNDIGFFWDNQPWDVSNFDNLGQRETKENLHIEMPPLPPAPINHLTQGEIEKKNEEFTTPVTNNKKRRRADEDGKRPMKNDGNDHDLHIWTERERRKKMRDMFANLHAMLPQLPPK1ADKSSIVDEAVRHIKTLQQTVENLEKKKRQRIQSLSVSVSPIVCESAVTDPQWNPYDSSSLSRDNNALTITGTQHASNPSFVSAAVANNSPFFKTWASQNVVLNMCGEEAQFSICAEKKPSLFTTIAFVLQKHKVDVISASILCNHNVNRYMVLTHASRALLQFADANTVEETFKRAAEEIMMWLG.

>Aradu.5CX4U.1 genotype-assembly-annot=V14167.a1.M1

MDQLGSRSEASPSSTNDKKIERRLIEKKRRNHMRMLYSKLNSLLSMDHNSISLKRCHWLIDQVDKAINYIKNLEEKVKMAKEKRESLLGMGRNRTRGAGGLRDLSKPPQLMVHEMGSYLQIVMTCGLDSQFIFSEIIRILNEENIDVKSVKSSLVENSMHHVVHAEISQFLLQLGATKVSERLKRFVNEPTSEIELQSDHQLLDFEIDTDELAELLDFLEEK.

>Aradu.29A27.1 genotype-assembly-annot=V14167.a1.M1

MGMQRVRNHYYSGAQILRSSSITPYDYIFTKPRSKAADKKQAAVKHSQAEKRRRMRINTQYEALKNLFQNKTKTDKASLLATTIEIVKNLRKNSILQEASSSSKKEMNKVTNQKKMQEACSPRNDDVFPSWEDKWSLKHEEDLMKATLNCEDKPGLMSTIAKAMGSVEAKVVKAEMVNVGGRTRVVLWVKDSNGDRKGEEILKRTLSTLMQRPVPKKRRFTQ.

>Aradu.TC25V.1 genotype-assembly-annot=V14167.a1.M1

MARSAKGHQEELDDDDDEHFTADTSSPHKVKLDESNSGKRVNPHRSKHSETEQRRRSKINERQALFLFPLLFQVLRDLIPQNDQKRDKASFLLEVIEYIQFLQEKLQIYEQTCEGWNQEPTKLIPWRGHHGPAENKADTSQAMQNGSAHEKNDVSPLLPKNVQNPIESDLSTTTIQKGHTIGSAVEAVPLGMQMRLDAFDPVVSSGMPNQHLHQPISNADMSFQIQPQVLFSKPSRGNYMVSDNVLMEHEELTNESESQSISNAYSQGVLDTLTQALQSSGVDLSQTSVSVQIDVGRRSNPGFTPSASSSKVYGNQFVTNRVNYCSEDSDQSLKRLRRDAT.

>Aradu.V7BYP.1 genotype-assembly-annot=V14167.a1.M1

MNALISKVARGWHKEKGQAEPPQDPAVAARKVQKADREKLRRDRLNDHFHDLANTLDPDRPRNDKATILTDTIQMLKDLTAEVNRLKTEHKALSDESRELMQEKNELREEKASLKSDIENLNSQYQQRARMMFPWTAIDHSVVMAPPPYSYPVPIPIPPAPVPIHPTLQPFPYFGNQNPPHIPTPCSTYVPFSAPINAALDLPSAQYASASHVPAQKDCRSKSPPHRRITDADRCSETHDVATELELKMPGSSTQEESSSGGTKKVKHSGRKDRAVAEGSASSRYSTSHGLQDSSNSVGDIPKADS.

>Aradu.A88Q7.1 genotype-assembly-annot=V14167.a1.M1

MDVDIFKTSTASDSMDMMAMMMQQMEKLPPPELSEPPFYTTYPSQTDFHNNNNVEPNLINPTFIFQQQHSMAPPPPPPVITTNNDDIITSSFPYPPPPPPSCSDGSNKKNSMAAMREMIFRVAVMQPVHIDPESIKPPKRRNVKISKDPQSVAARHRRERISERIRILQRLVPGGTKMDTASMLDEAIHYVKFLKKQVQTLEQAGGGRTCNNNNGFTGFSSSINGSNYPAMVKGCHQPYPPMLMSSSASKQLLSASSILLCSVLLCQCCNLSSSLHLHC.

>Aradu.85INT.1 genotype-assembly-annot=V14167.a1.M1

NDSYGNKHRKLEGSFQEENRAGDSKAEVGASLVAGNKLARQGGGGGGGGGAAKPSCEQPPKQDYIHVRARRGQATDSHSLAERARREKISERMKILQDLVPGCNKVIGKALVLDEIINYIQSLQRQVEFLSMKLEAVNSRLNMNSVVECFPSKDVQVGNQPFDIAGMIFGSQATRGYAQGSQSGWLHMQLGGDFDRVA

>Aradu.WC9V5.1 genotype-assembly-annot=V14167.a1.M1

MLVGEDQHKKSTKQQRGGGAMKVLICHERILGRVDYLMEISSELGIMEDPNSFLWHLSSIDTCATTLAVFGDSLQKNNPLFCNSNLMNSKISMMETTTSPTTTIIERPAKQLRSNNTSNWSSHINKTPESHFVGSCSNNLLSFVDNTNHHHHQLGLVMKPKVEIMSSSSPNNIDTQGTTLLGNNNNHHHNHENYLFKESSCHEAKNFGQRPKLSSHQPHDHIIAERKRREKLSQRFIALSALVPGLKKMDKASVLGDAIKYLKQMQEKVSALEEEQKKKKTVESVVMVKKSQLCNDDEDSCSSETEPLPEIEARFCERNVLIRVHCEKKKGVIENTIIQIEKLHLKVINSSVLTFGNFALDITIIAQMDMEFSMTVKELVKNLRSAFSTFM.

>Aradu.HK2E0.1 genotype-assembly-annot=V14167.a1.M1

MDISSLRGLTDLEIMEDPTYLHQWHLSSIYEPTLLPIAAAFGETLQQHSFSHPSFNSKTSMETSLSNIDRPTKHLKNNSWCPTKTPSEAQFASCSTLLSFVDPNYINQLEVVKSKDEMVCPKMNDTTPKDMNFQGTLGNQYVFEASQATKHVGPRSRLSQPQDHIIAERKRREKLSQRFIALSALVPGLQKTDKASVLGDAIKYLKQLQEKVKALEEEQNKRKAVESVVFVKKSQLSNDAEDASLEYEGIFHEALPEIEARFCDKNVLVRIHCEKNRGVVEKSINQIEKLHLKVTNSSAMTFGSWALDITIIAQMEKGFCMTVKELVRNLRSAFASII.

>Aradu.LYC6U.1 genotype-assembly-annot=V14167.a1.M1

MSQNSEVRSHQKRLMEEVEIHVSNGGVVQEVGERLSLPKLMMMMMMSKSNSSKSIVNSGNTAKKARRSSSECLDHIVAERKRRQEITQRFIALSATIPHLKKIDKVSILSEAIAYIKQLKEQGKKLEEESRKKNRTVVESVSLVNKRHDAACELVEARALEKQVLIRIHCHGHKAVPQVFSHLTNLDLSIVSTSVLPFGTCAIDITIVAQMGEKYRASMKDLVQSLRLAIPLC.

>Aradu.FPU47.1 genotype-assembly-annot=V14167.a1.M1

MELSQLGFLEELLLLAPRRVEETCWNNGFNDLLPTTPPPSASVSASWSFDSLDDNPSLNPSFSSFSTPLDHRFDSSYAASFPFLDAFTIPPELHHSPPPLLPQQEHNHNNHHPLVEEEEEELGFLGNDDNNQSLEERNSGRCKVEEQQALEVPAVVFNMGMCGEKKVPKSKKLEGQPSKNLMAERRRRKRLNDRLSMLRSIVPKISKMDRTSILGDTIDYMKELLERIAKLQEETMEEEGKNQIHLLGISKELKPNEFDVERRDHDTRISICCATKPGLLLSTVNTLEALGLEIHQCVISGFNDFSMQASCSEVAEERNCISTEDVKQALFKNAGYGGRCV.

>Aradu.33ULW.1 genotype-assembly-annot=V14167.a1.M1

MASSRERRREALQQKLNQLRDVTNSSGVNKASIIVDASRYIEELKQKVEGLNSELVITETSSSTSQIDDELPMVTVETLEKGFHINVYSEKNCPGMLVSILKAFEELGLDVLDARVSCEDTFQLQAVGGESQKNNDTIDAQVVKQAVLQAINKSNY

>Aradu.X1TYZ.1 genotype-assembly-annot=V14167.a1.M1

MLQPSFSSENSESDNLQNTVGSSSSPVIEEVPQDLTAENSNNKNKKNNTMKQPRRSSSSSSSLRCSSPRRYILSFDNSTMTPATPNQLQEDPYYANNNNNNNNNNNNHKNKKNKDPSSCDYSSPKRVVPICTNNSENKKEEGAITTKRARSSSQTMDHIMAERKRRQELTERFIALSATIPGLTKTDKASILRAAIDYVKQLQEQVQELEKKNKKRSRESVILVKKSKSNNEKILLEESTIISSETRRSEDGGTGLPDIEARFMGKDVLIEIHCEKENGIEMKILNQLENLHLFVTGSSVLPFGNSALGITIIAKMGDSCEMTVNDVVRNLRQVFLKAHQNSHGI.

>Aradu.4P1MR.1 genotype-assembly-annot=V14167.a1.M1

MSSDVENAMRLQQEEKKGILMSCPSSLGLGTSGSEMASSNSEPSNVANPFLASSAWDPLTPLTQLQTTNTTTLVGDPSTSSIPIPIPMVSHHNNEFSNSLLYTLVLENQQAPHIVQYMSRDMVPIPKVQVPVSYGSGSFSEMVGSFLQSPNSEHSQGEGEGEDSGSAPSGNRRRKRGHEHNNNSTFSPNKNAEDCSGKRKNQDDEKKAKVEENKDKSGQSGETPKENFIHVRARRGQATNSHSLAERVRREKISERMRLLQELVPGCNKITGKAVMLDEIINYVQSLQQQVEFLSMKLATVNPELNLDLERILSKDILQSRIGGYGGGISCSQAFPSSSFQGTLVPMPTTSNQLPPLPQSLLDHEFQNLYGMSYDSSTALGNMGHNGGSKPEL.

>Aradu.38JU0.1 genotype-assembly-annot=V14167.a1.M1

MALLEDHHHLSSSNEFSNFVVYDTISATPFSSHGSSSTSFLVEENKVEEYGERTTTRKRQSGEAGQGKKKRRRKPRVCKNKEEAENQRMIHITVERNRRKLMNEHLAVLRSLMPESYVQRGDQASIVGGAIEFVKELEQLLQCLEAQKLKLVHQGMAAQQSNKNNNEDELMMMMMMVMGSAPFSHFFVHPQFTWSQAPNKYASHHNKTNNNNNNNQAAMADIEVTLIETHANLRILSRKSPKQLSKLLSGFQNLSLTILHLNVTSMDPLVLYSISAKVEEECQLGSVDDIASAVHNLLKMIEEEDSLCSLYGEVTKSTPSPLQDSLENSNTEYSLS.

>Aradu.MN4MZ.1 genotype-assembly-annot=V14167.a1.M1

MGGHENAMGFHHGNESILTNNVNVSEMDMSSSMSLARSSSDVVVPNSNPFLASSSSWDPIVSLSQSQTFGGSSMVTTHNSDFGNSSSYPFVQYMSDSTNLEGIMVHKIPSFGSGNFSEIVGSFCQEGSSDIPNMGFRPSYNHGNDAGTERATMNEQSQVEDSITEDGAPGTAPSGNRRKRMLDHDSSFSPNKNAGGDELKDSPRTISDGAKEHEKKAKVEQNVSADLRGKQPAKQSKENSPSGEAPKENFIHVRARRGQATNSHSLAERVRREKISERMRLLQELVPGCNKITGKAVMLDEIINYVQSLQQQVEFLSMKLATVNPELNFDVERILSKEILQSRLGHGIVGYGVGMNSPHPFSNGSFQGNMAGMPSTSTQFPPLPQVI.QNKIKYHKKSNKFRSLWL.

>Aradu.ATP30.1 genotype-assembly-annot=V14167.a1.M1

MSSSGAMQKKHEENSFLDTKTMAEGSDPSPSPTNMVAAADEGNTENSNEENLNLSLDEFYHQNHNPPPQTTEGVSTFENGTSSISMDIENTQNLSLNIGNSYSNKGTTTPLVQEIVIDHDDAFHYEKSNWDSTIHELLDLGFSNHNENQQAHDHQQFQLCEAQNCSQSYHSSDLLQLHLNPNQIPCITNPIQNNTPNFQHSMGFLGDLPVGSDYDTSLHLNPAFGVGELFPSLPHGYTRLTDSRSGFLFGGGDEMEGNGACYQDGDGSQVDIGVMEFNRARACSVGGRGRRGQGTMPIDKQRREQLNDKYQILRSLIPSPTKVDRASVVGDAIDYIRELIRTVNELKSLVEKKRIGRERFKRHKDENDASESCNIKPYGDGSIRTSWLQRKSKDSEVDVRIIDDEVIIKMVQRKKINCMLFVPKVLDELQLDLHHVAGGHVGEYCSFLFNSKIIEGSSVYASAIANRVIEVMDTQYAAAAVPHNSSY.

>Aradu.LS2KI.1 genotype-assembly-annot=V14167.a1.M1

MVPEKVKKQLALAVRSIQWSYAIFWSSSPTQPGVLSWGEGYYNGDIKTRKTSQVAEHNSDQIISLQRSEQLRELYSSLADSKSCSQTKRPSAALSPEDLTDTEWFYLVCMSFVFNIGQGLPGRSLAYGQPIWLCNAHSADNGVFCRSLLAKTVVCFPFLDGVIELGTTDPVSEDYSLIQVIRNSFLDILETNLPNNPGANLNTRNKEECGVACEGFDHNAYGLKLTPEVIGYELINITSPTTSSNALQANQQTDGRTMFPTEFSNYFHNSRNSSDCLSENHCAQDLQQCNDNPRTTLVNLGSDDKHYQRLLSALPIRPDDDRLIMRVHLRNFRGESSFAIWKQLGSMDCQRSRRGGTPQNLLKKVLFEVPLMHMDGLLESQEKIGSKDRMRLSEVDDVGMNHALSERKRRAKLNERFLTLRSMVPTISKDDKVSILDDAMEYLRKLEEKVRKLEAEKDVTDLDGIVSTRTSQDMVEKASDNNSNKNSKSVSNKRRACEAAGDEEMNNSEIDVGIYVSESEVVIEMKCPWREGLLLETVEALSSLHLDCHSLQSSKSHGTLYLTIKSKFRGANNVAPVKRIRRTLQKAAMKS.

>Aradu.76WTQ.1 genotype-assembly-annot=V14167.a1.M1

MEGPMQESSATSLKISLLKPVLRGVSMHNINSRLNMMNKNREISLCGLKMRAVQLSTDHSTSIEEVIEYIHFLQEKVHKYEGSFQGWNHEPEKLMPWRNNDKPAESFEACGTNSGSGPSPTLLFASKIDEKNITLSPPITGSSQKLEPGLNTATPFKNMDHPPGITNNTFPIPISSPPNFFPPRKSIGLGEMPHVTHRLPSDAGNGIYQPSVECQTVTATNEKLKEKELAIEGGAISISSVYSKGLLDTLTHALQSSGVDLSQASISVQIELRKQENIIRPTLPMSMCGIKGSFACKCKGCNHWRRIDGLWNSNSKSARNLDGYQ.

>Aradu.WD82P.1 genotype-assembly-annot=V14167.a1.M1

MIIRLRNNNSSFSVPTQIVPHAYDSAFASLQFQYQWPLAFEGFPEDRAASASKSHSQAEKRRREKINAQLATLRKLIPKSDKMDKAALLGSVVDHVKDLKRKAMDVSKSITVPSKTDEVTIIECDPDQDESYAKVKILKHNIVISVCCDDRPELFNELIQVLKRIEETGTESYRRMRRRARPVVLAAAVDGCGWRMRRRAR.

>Aradu.Z40HV.1 genotype-assembly-annot=V14167.a1.M1

MEHAALDSIQFNEQIQGLMAPAPESTNSFTALLELPPPQAVKLLHSPDKAPTGSEKPPCHASILKPYPSIMSIIDGTRKLTFPSNTALIERAARFSVFAGEGPLAPPVAEVKNEPPETDSNPSSTQCDPAVENKGAKRKEREKKVKARSKKSKSVAAAESSGDGEKLPYVHVRVRRGQATDSHSLAERARREKINARMKLLQELVPGCNKISGTALVLDEIINHVQSLQRQVEFLSMKLAAVNPRIHFNLDSIMPNEGASLMDSSIPNMVSPLMWPEIPNNGNREQYQQPWQFDAFQQPVWGREEENTNFMTPENSMLSYDSSANSGRYGLSFYFTGIF.

>Aradu.KWH4D.1 genotype-assembly-annot=V14167.a1.M1

MALARDQIPHDSSMGSKVQSCVFNENNEYHKSVLEEEDGSQSTNGFSNDSAITHSPPLCGNAYAYKATNYQLEEEQQSLIDFKGSCYNTLTQVASESLLNFEQNRMVPGNSYMKDDTNVWDNNLHHQWSQISPRSTSELRPVQDSSCFQSSSSSYSTIVNSAKEKQLHGESSSYGWFYSQQTIPAHSIQDPAVQEPISKKRISMQAEKMKAAKKQCTDESKMPKSNKLGASKDPQSVAAKNRRERISERLKILQELVPNGSKVDLVTMLEKAISYVKFLQLQVKVLAADEFWPVQGGKAPDISQVKEAIDAILSSQRSEKQVQQPQSR.

>Aradu.U3SNU.1 genotype-assembly-annot=V14167.a1.M1

MMMFDDMGFCGDLDVLCGPLGESDMTARQTEPDAVVEDDYSDEEIDVDELERRMWRDKMRLKRLKEQSKAKEGIDAAKQRQSQEQARRKKMSRAQDGILKYMLKMMEVCKAQGFVYGIIPEKGKPVTGASDNLREWWKDKVRFDRNGPAAIAKYQADNAIPGKNDGCNAIGPTPHTLQELQDTTLGSLLSALMQHCDPPQRRFPLEKGVPPPWWPTGNEEWWPQIGLPKDQGPPPYKKPHDLKKAWKVGVLTAVIKHMSPDIAKIRKLVRQSKCLQDKMTAKESATWLAIINQEEALARELYPDYCPPLSSGGGSGSLVINDCTEYDVDGAENEPNFDVEDRKPENLHPSNLGMRGRLQVQKPSLPIKGEVVTNLDFIRKRKSDFNLMVDQKIYTCEHPQCPYSEGRLGFQDRSSRDNHQLNCPYRNNSAADFAGPNFHVNEVKPVIFPQSFVQPNTTAQPASLVPPTFDLTGLGVPEDGQKMISDLMSIYDANVVGNKNTSSNNFVAENQNQNIPQQCINQQQESFFPNQGMVMEGNFFARDDNNQFDRFKAMNTPFETTTTAAATAVAAATNPPNNNNSNFNFMFGSPCDLGSFDFKEDLQGGMGMDSLHKQPDVSIWYQ.

>Aradu.C74HT.1 genotype-assembly-annot=V14167.a1.M1

MESSSPSWLHDLEMEDDYNNFFPKEYRMNNSIDDYDEDLFFAHDMGNSIMFESPSNNNNYSSVDETSFDDERPSKFLKTSTNTDNNSYYSSLSPNFSSLSSSSITSFQPQILCFDNSNSSLNNNDNTTQLYDLDYTTLNTETKLKEVVNGSSKNQNLVTKSSSKGSKRSPSNAQDHIIAERKRREKISQSFIALAALLITNIRILTIQIDKVTVLENAIKYVKDLKIRLTTLEQENNEKIKEIVEEPSVIVLNKRLPHDNESDDESVIVDNTSNHDSSSLLHHVEARVSGQHVLIRIQCQKHKGILVKLLSEIQGHNLLVLNSSALPFGDSVLDITIITRV.

>Aradu.573UI.1 genotype-assembly-annot=V14167.a1.M1

MGDRENFEVDRDRDRVNYSTSMASDWRFGVGNFANSMVDSYVTNFWDHPNSQNLGFCDINGGSSNMNVIGKDGFGYGRGSHDHRTLEMGWNHASSVLKGDNNAFLPNGPAMLPQSLSQFPSDSGFIERAARLSCFGAGNSVDMVNSFGIPQSMNLYARIGGTMQGTGDALLGHGLRSAALGGQSHKSDPNVVEDAKDDKEMTLQDDKRSENLAVSHDEGKQALGGSANESDRAESSGRDDSPMLECTSGEPSNKGLSSKKRKRIGLDADKDKVDGSPEQPGAAVKENSGSRQQKGEQQPTPSTKGSGKNAKQGSQASDSPKEEYIHVRARRGQATNSHSLAERVRREKISERMKFLQDLVPGCSKVTGKAVMLDEIINYVQSLQRQVEFLSMKLATVNPRMDFNIEGLLAKEILQQRAGTSSAHGFPPELSMAFPPLHPSRPGLIHSTLPSMANSSDILRRTLQPNLAHLSGGFKDPNQLPEVWDDELHNVVQMTFPTSAPPSCQDLDGSAPSGQTKTEP.

>Aradu.LW8Y2.1 genotype-assembly-annot=V14167.a1.M1

MEDGRVPKVAQDQKWELHFGHCCRDLRREAASGCLYIIGIVKRALEMSDKEKFELDRKEDPMNYSTGMASDRRFGSSNLPNSSVGLAGRGDLNGSSSCSSASLVDSFGPNFWDTTTNSQNLGFCGINVHNSGSSLNPIELRKDGFGFGRGGHDHGTLEMGWNQGNGFIPNESAMFSQNLSRFPSDSGFIERATRFSCFGGGDFGDMVNAYGIPQSMGPYAGTGGEAIHRARDGGQPQESNPNEAEAEAVKDATPSVEYLATKASPLKNDKRSENHVMPQNEGQQGPSRPPNETDRAKSSDDGGVQDDSPMLDGVSDEPCLKGLDSKKRKRIGQDADNDKAVELPTEGAGDISESQQKGEQEPTPTNKASGKNAKQGPQVSDPPKEEYIHVRARRGQATNSHSLAERVRREKISERMKFLQDLVPGCSKVTGKAVMLDEIINYVQSLQRQVEFLSMKLATVNPRLDFDIEGLIPKDILHQRPGPSSALGFPLEMSMAFPPLHPSQPGLIQPTLPSIANSSDILRRTVHPQLASPASGSFKEPNQVPDVWEDELHNVVQMSFATTTAPMSTQDVDGTVAATQMKVEL.

>Aradu.4NW8B.1 genotype-assembly-annot=V14167.a1.M1

MEPAQLISEEWGSLSGLYTAEEAEFMTQLLAGNYSVSEKHYSSVAMPPSSAFWPCHESTKVSFKGINGNSYLPPSHIENANYLCFSQGSSSSTENGNIYSYDTTTNFDSMSTDNCLEGAKFSPQSNDNSRAQISGIIDDDAKIECERMVFEPAEDLENPTKRLRSSSEVPRNMRNVKSRKENPLPSSYTSNSEEDKGPAAPKNRRSSSGAATDPQSLYARKRRERINERLRILQTLVPNGTKVDISTMLEEAVHYVKFLQLQIKLLSSDDLWIKKMEPGEEIYAEWSSLSGSYTAEEADFMNQFLSNCSHTQQLHANLNAGIPSALWYGHADINSSSFYAADASNNMFPIIDSDNLNDPLTNLVDPSLSTYTDKELGVDVIADKNVPSQPVSEPAQENITSKLEKPRKRSRSSNEILKNKKNVKTTKKPKSASISNIKEEDRCPGQGENLSCSCSEDDLDTCHELNEGESSSLSLKYSKGLQLNGKSRSSRGSATDPQSIYARRRRERINERLRILQNLVPNGTKVDISTMLEEAVQYVKFLQLQIKLLSSDDLWMYAPIAYNGMNIGLDFNITTPKQNNNSRYIA.

>Aradu.77XDI.1 genotype-assembly-annot=V14167.a1.M1

MESDLQAPSSNGLTRYRSAPSSYFTDIIDREFYEHIFNRPSSPETERVFSRFMNSLRDDAAPEDDSLHLDSSSVKEEHDITPSQPLLFQHQQQQQQNNVNSFNYQSTSRPPLPNQNQNLASSGGVEGVYSNNNNNNNRLPQMKNQSNLVRHSSSPAGLFSQIHIENGYVVDMRGMGTLGAVNKSVEEVKFSSSRTRRLKNPQNYSSSASGRMSSIAEIGNRDSREDNPDAEAFGETHGEDFITEFAPWDDSVAVNDIVAGLKRFRDDDVKPFSSGLNAAETQNESRGQQAAPLAHQMSLPNTSAEMAAIEKFLQFSDSVPCKIRAKRGCATHPRSIAERVRRTKISERMRKLQDLVPNMDKQTNTADMLDLAVDYIKDLQKQVETLSDCQAKCTCSSKPQQ.

>Aradu.6E0QJ.1 genotype-assembly-annot=V14167.a1.M1

MESDLHRHPPMFLDHHHHHHHHHHHQQMNTTTTNNNNNSSGLTRFRSAPSSYFSSIIDKEFYESIFNKPSSPETEKILARFMNSLANDEPEDDSLLGVAAATSPTTNNNKNLSPSPQPPQQVVQQISQVKEEEISINTINTNNTLQPQPLPSSMNNESLVQQPQQQMNSMSNNNYGSLSGNNPPTQSFYQSSGRPPLPNQMKTGRGNASNLIRHGSSPAGLFSNLNLEGYAALRGMGTMGAAASNTSEDANFSPVARLKNPPTFSSSGLMTPIAEIRSTSNTLNNPESAEAFAESQSSDFMSGLPVGSWDDSSSSMMSDNIAGRKRFRDEDVKPFAGVNAADTQVKTEAGQAPGTPLAHQLSMPNTSSEIAAIEKFLQCSDSVVCKIRAKRGCATHPRSIAERVRRTKISERMRKLQDLVPNMDKQTNTADMLDLAVDYIKELQNQVE.

>Aradu.UKN3W.1 genotype-assembly-annot=V14167.a1.M1

TDLQLLCLVSGFVNADAKAVDKASVIRSKHSVTEQRRRSKINERYVQRASVISSVLSASVFDVGALNHDAFGRQAVNTVGIVICYEATSLHKHHHNSMVTDANHASSCSVLQARNEGFQILRDLIPHSDQKRDTASFLLEVIEYVQYLQEKVQKYEGSYQGWGHEPSKLMPWRNSHWRVQSFVGQPQAIKNGSGPVSPFPGKFDESNISISPTMLSGNQNMIDTDQGRDIVSKASEGQTDLASKGIALPLGMHPNMSIPVRSDGVLSHPLQGSVSEAQSTECPAASEPLSQQDELTIEGGTISISSVYSQGAVKNSNGYEYELLNNLTQALQSAGLDLSQASISVQINLGKRANNGPSCGTSSPKNHDMVPSSNQAFAQFRDAGSEDSDQAQKRLKTFK.

>Aradu.YG73I.1 genotype-assembly-annot=V14167.a1.M1

QLFQLHTSSLDTSSVDQSSKVVAATTKSYNSSADPSPPFLSHNHSPESSVVENGDQVTQNTPPPINMADNKRKTTSNSSSFNNNKELSAGREGRSSKKQRKSNNGGSGVKKGEEKEKEEAPTATGYIHVRARRGQATDSHSLAERVRREKISERMKMLQRLVPGCDKVTGKALVLDEIINYVQSLQNQVEFLSMKLASVSPFFFDFAMDLDNDNNNGHLVRPLDQRSSNVFSEYTGGQFWEVEEDQRQKQLLHPYGLGSNLGRGKLSLVSTMSEPES.

>Aradu.5E40Q.1 genotype-assembly-annot=V14167.a1.M1

MMAAFSSYQYHPNFLPLDSTFFQNLNTTTTTSVPIPQSILHHHQEEGFSDFNNNTISCVDQISSCNKVSFISNSDNEPSNVSVTTRNNLSPQSSMVVDKLEIGEQVTQKDGTTRKRMRKSNNNNGGGVVRREKISERMKMLQRLVPGCDKVTGKALMLDEIINYVQSLQNQVEFLSLKLASENGSQFWDTEEQRQKFLHPYEFINNNNLCSFH.

>Aradu.687AB.1 genotype-assembly-annot=V14167.a1.M1

MEMEGFAASSERSSGVGGGYASSSSLVLDRVKGELVEAPVKLERKGVFPERTIEALKNHSEAERRRRARINAHLDTLRTVIPAANKMDKASLLAEVIRHLKELKTNAAQACQGLMIPKDNDELRVEEQEGGLNGFPYSIRASLCCEYKPGLLSDIRQALDALHLMIISAEIATLGGRMKNVFVIISCEEQSFEDAEYRQFLAGSVHQALRSVLDRFSVSQDILESRKRRRISIFSSSSLEDFL.

>Aradu.8L7DK.1 genotype-assembly-annot=V14167.a1.M1

MEATSMTHHLLKGFCDGTQWKYAVFWKLNHRFPMTLTWEDGYYGYQKTNEAAESMLDDINFKFPAEVYSSSGEIIEDPGDYSAGLLMAEMSHHKYSLGEGVVGKVAFTGNHCWVSEDILTHGLDANLMPECPDEWLLQFASGIKTIVLIPVLPQGVLQFGSFQAVAEDLEFVTNIKEKFHSIHLLAANTTALNYGVDCQDWSFSDPTNTFMDILDESSNITNSTIKTDVLARIAPSVNASTRLNPAMLSQVETKDNLEEEIWPTSPWVNDVGVFQEISNGLGLYSGKTEQQFGSNETGYEDIKDLNGFLAFPPESELHKVLGSAAPHGKTRNSMSKHTSVVDTYSNSTFISNNKEHGDIESLEHPEDVDPKYLLDAVYGDLFSACNDTSSISNSFRSPVSKKMPFTGLIHPRNSCEESSMVMKDVKSDLKAAVRVMGRDAITSPSFDGNSSLLTDEPQEEKVYSHLQSTNGPKISSTYKKRARPGTQKSRPRDRQLIMDRMKELRELVPDGGKCSIDNLLERTIKHMLYLRKITSQAEKLKRFAHREVPKCSKQKINYNQSGRSCAYDLESEQAWPIVIEDLECSGHMLIEMVCNEHGFFLEIAQVIRKLELTILKGVLENRSSAAWACFIVEAPRGFHRMDVLCPLLHLLQRRKNPVPYKS.

>Aradu.0572C.1 genotype-assembly-annot=V14167.a1.M1

MLPFGGRFYGFESWLDHPHQHHSSPSDGFFVPEAPFKATDLLFDGAGRSSKLDNNRKSTEACKSHREAERRRRQRINAHLSTLRSLLPNTAKADKASLLAEVVKHVKRLKKEADDVARDPSKPDAEAETWPFPGEADEATLSYCDGEPNLVKATLCCEDRPGLNRDLTHAIRSVRAKAVRAEMMTVGGRTKSVVVIQWPAASGGGDSKEGVEVGALERALKAVIENRALVGSGMGRFVLGQKRAWNSYDSPDEFLLTRDASK.

>Aradu.FEY54.1 genotype-assembly-annot=V14167.a1.M1

MLQRLLRRRSAPVTAPATICLAELSSDEIVCSLQRRSPLLQRRFSSGNNLRKRARNFIKLYEICCAFPPTIFSYGDLLHQRFSSMRSVAQVFPVASSCILRLFHLYRASLSIWVFDFLPCQKKLEQLDPAPKSDDRPAKRSRLKSLRDSDSAALEISKEELDFRNLLEKDLEKFNKFLVEKEEEYIIRLEELQDRVAKVNVSSEELMKIRKEIGALHVSLVEAVRPYTILRTSPYCKRIFSRSLLKNDDTNICFRGFWSILSCVAALVPKGELDGEIGDAHMAMQARLMSQALQKLSHSLSVSQCMLIFINQQLSQYRTCFGGFRVTTSVGKKREGKGTKHLATEKQRREQLNGKYKILRSLIPSPTKMDRASVVGDVNEYIRELLKMKAKDDATESCNIKSFSNPDRCIRTSWLQRKLKDSEVDVQIADDDVTIKLFQRKKVNCLLSVAKFLDELQLELHHVAGGHIIEGSSVYARAIANSVIDVMDTQYAAAVPHTGSY.

>Aradu.8U0A6.1 genotype-assembly-annot=V14167.a1.M1

MALNTYVNGDALQNSIISEIFTTNFHELTAPKELTPDYYYHQHNQEGLFPNNFFFDPYFDLNNGFFHPEILSYHQLGLSCTSSHDLFISPNKTEYSNVNTIFSSPKRQKYFHKDEDEERQLPPPFKEYSSPNLFDGFTMNSSSLLPSEGAALPEELLLPAAVATPSSDFMVPNVVLNSFCVGINNDSKKKKNSERTISAQSIAARERRRKITDKTQELGKLVPGGSKMNTAEMLHAASKYVKYLQTQIGMLQLMNTLQKEDEVAPPSEDLRALITSPCVQENLYSEELCFVPKDFVTALTNQRDIRSKPTIFKDLKELIETNNVQKKA.

>Aradu.V6ZNL.1 genotype-assembly-annot=V14167.a1.M1

MDPGAMMNEGSFGNGGGNTVPFSLAEIWPFPQAVNAAGGGALGLRRPQFGLGQFGDFIAGPNREPGARRESEEESPKGVSTSNVAPNAVLQFVYVVSSGKPADQNNQTPPPDPPRQDYIHVRARRGQATDSHSLAERARREKISERMKILQDLVPGCNKVIGKALVLDEIINYIQSLQRQVEFLSMKLEAVNSRLNTGIEAFPPKDVSLQRFLYWDVEFC.

>Aradu.YAX06.1 genotype-assembly-annot=V14167.a1.M1

MFNSSRKGMTRSSSLLHVNKDIGELVWENGEVKVQGRGAAVEERAAKLDRFYCSSILDQSKKQIQQGCSNFKLISNTYSSSLSEQCKKPRIIDFSGSAQAKTKSHTLQHLGTCNNNNNNNNNNNNQRTSLGGGMVNFPNFLVPSLFLNKSSSSAAAATSSKGHDHSSVVIDSSNNNKAATQELKETPFHQVQPLDQHSHGQKLYHGASSSSASPPPPNNNIEQHLVVASSPVSSIGASNDPDIGIMRKQHEEYSNITDHDHDHDDTTTYISDDDDEETEDVIIAKETPAAGTRAKRSRDPEVHNLSERVNKRIHTLKELIPNCNKIDKASTLDDAIDYLKTLKLQLQIMSMGRGLCMPLMMLHNHHNQLMCYRPSSGTGIPHQNMFDGFFNQMRPMIIPPSPPFINPTPPLAPLSIANSSSRDVSHVQAINNGDQVSLHQHTTSSSYPFYFPTIINQEEKNNYGME.

>Aradu.X5F2F.1 genotype-assembly-annot=V14167.a1.M1

MEFLGAFPNNIELDCFKMFSNEKEHDFTYTTTTTSQLFLDQSSSLLGEDDELNFGLVQSTLSSNFISDENEHYLFHSLDANPNTKMLHYKSQEESSYNSGGFSGGDTTFFNANMDLTTNYYYSSNYHDDNVLANDVSISMELLENYDHYQCHQMEHVDVVPNNKQLELKRKIVEVPEFDVSAENNTSNGSKNQKKKHFVAKDEQDCMKNERCRKRKVVRNGNEGEERNNNVGLDGQSSSSNINICEDDNASEENNGGVTSVSHSNGKTRTIRGTAADPQSLYARKRRERINARLRILQGLVPNGTKVDISTMLEEAVNYVKFLQLQIKLLSSDDLWMYAPLAYNGLDIGLKLNNLKNFSSSP.

>Aradu.1M0AQ.1 genotype-assembly-annot=V14167.a1.M1

MDGVFNLPEATRSDFLRSLMHTFGCTYICLWHCHSSSKCSNLLFLDGIYNNVSSTVAEETLFNLYQRLTFDAANDEWVPGAAFRRHMAYLELQQLDLLRLASAVIQTQFYLEARIETAIFMGCSKGEIELGFEFEFHSLYVQIDMKAAVKSLFPEDFSREQIHHPASSSSCSCPSDEFSSLGGVLLPAPEGEQEAIIRAILHVISSSSSPATTTSQPPPYNNSAFRAYPSTTYNLVTSSSRRFSLMKRSIEFSRTLHLMRIRDRFHFQRQHQPPHPLIIGTQNANNNKHLHHIILERRRRENENKCFRELRALLPPGTKKHKSSVLIAAKEALKSLIAEIEKLNIRNQQLKTLLGSSSSSYGNVHISSSPSSSNERLNVAVSHVAXXXXXXXXXXXXXXXXLSEEKARLIIINRIL.

>Aradu.FJ441.1 genotype-assembly-annot=V14167.a1.M1

MSFILDLRRLALQAFQRQQAPNPVSVTALPQQPPGIRPRVRARRGQATDPHSIAERLRRERIAERMKALQELVPSINKTDRAAMLDEIVDYVKFLRLQVKVLSMSRLGGAGAVAQLVADVPLSAVEQGEEIEGGGNNEQAWEKWSNDGTEQQVAKLMEEDVGAAMQFLQSKALCIMPISLASAIFRMPPSEPSSLIKPESNSHS.

>Aradu.UB339.1 genotype-assembly-annot=V14167.a1.M1

MTFTTIRFESLLQAPMPPTPSFNTLDYSLDHNHHHHHLMQFRQGESSGEHSNGIADYIPQQQQQLPPAPPPSCFYNANSSSFDKLSFADVMQFADFGPKLALNQGKPCEEPGIDPVYFLKFPVLNDKMDEHNIMMMNHESDGGGGREEVETEAENNNDERFNNGEEEDAAMGGADQEDGRMKGEEETARISEDNNSVQIRFLGHGEDLVLNQKRNNNSSALMQENNKNMKRKRPRTVKTSEEVESQRMTHIAVERNRRKQMNEHLRVLRSLMPGSYVQRGDQASIIGGAIEFVRELEQLLQCLESQKRRRLLGEAQTRQVGDSNLGTQQQQPLQPPFYPPLASPSEQMKLVELETGLREETAESKSCLADVEVKLLGFDAMIKILSRRRPGQLIKTIAALEDLQLIILHTNITTIEQTVLYSFNVKVASESRFTADDIAGSVQQIFSFIHANTSI.

>Aradu.D69CU.1 genotype-assembly-annot=V14167.a1.M1

MDGTAARKSQKADREKIRRDRLNEQFVELGSILDPDRPKNDKATILGDTIQLLKDLTSQVGKLKDEYAALNEESRELAQEKNDLREEKASLKTDIENLNNQYQQRLRNMFPWTAMDHSVMMAPPSYPYPMPMAVPPGSIPLQPYPFFANQNPSVIPNPCSTFVPYLAPNTLVEQQSAQYVSPPSHPGTRSHLSSKHDTRNKPPRDRESKAEKSEASNDVTTNLELKTPGSSADQDLSSGKRKCSKPLRSEGSSLGRCSSSHSVQDSSSSSVDGSRKANE.

>Aradu.V7DVH.1 genotype-assembly-annot=V14167.a1.M1

MPSIQELPFNNHESFIVPPQQQQQQASPYASFFNSRRVHPSSSSSMQFAAAYHHHEGATSSFDLQAELSKMTAQEIMEAKALAASKSHSEAERRRRERINNHLSKLRSLLPSTTKTDKASLLAEVIQHVKELKRQTSMIAETSSVPTESDELTVDDASDEDGKLVIKASLCCEDRSDLLPELIKTLKSMKLRTLKADITTLGGRVKNVLFITAEQDYYSSSTGNEDHHHQHDHNNSYQYCISSIQEALKAVMEKSNGGVGNEPGSSSASVKRQRTNIISSIS.

>Aradu.JI81D.1 genotype-assembly-annot=V14167.a1.M1

MVSSPSTAAPNFNMIQNDNSFVIRELIGKLGTIGGSSAEISPHSQTVALYNNNNTTPSSSCYSTPLNSPPKLNMPKPPPPSSSMALNSATVAEFSADPGFAERAAKFSCFGSRSFNGRTTTTTTTTTTSPLVMINNHNNNNNGAELTHQRSNPIHRVSSSPSLKSLGSQMEKICSSPLEMAANSSQEESTISEQNPNVEGAALKIASSSDMSSRKRKGSSRAKAKDSTKGGEGSEESNAKRSKGNEGEGNENNGRVKVEEESKGEEKQQQNNKSNNSKPPEPPKDYIHVRARRGQATDSHSLAERVRREKISERMKLLQDLVPGCNKVTGKALMLDEIINYVQSLQRQFLSMKLASVNTRMDLSIENIVPKDIFQSNHSLAHPIFPIDSSATPFYGHQSQQNPVIHNNIPNGSLPHNSVDPLDSALCQTLGMQLPQLNAFNEIGSQVINNNNNTNNAY.

>Aradu.25NPV.1 genotype-assembly-annot=V14167.a1.M1

MLHCLNSNASGSEMTVLERQRATIKWQHNNNHHQGYSSSSSPSPSSSSSISLVQVQSSSPTCLAAGAWPKLDNNFLMGFTSSPSSGFPLPPPPPPTTSAASFDLNSAISRTSSCPLPSLAEGKETTTPNNKRKTDKFHNTKVVASDNDNKDKRVKVAAPNDSATVGKKGTQACGDNSNKDNSKVSEVQNQKPDYIHVRARRGQATDSHSLAERVRREKISERMKFLQDLVPGCNKITGKAGMLDEIINYVQSLQRQVEFLSMKLAAVNPRLDFNMDDLFTREVFPACAASFPAIEMSPNMANTASYLHFNSSQQIDAGINPQDISLRRTISAPVSAPETFLDSSCFTQTLPSSTWEGDFQNLYSVAFDQARAASFPSQLFTGSQQIVSCQNHFSPLSLISLKQKVKAEESGY.

>Aradu.WNQ8E.1 genotype-assembly-annot=V14167.a1.M1

MEQATGVLRGSTHDDVVLESIQFNEEIRGIMAPAPAPENASSFTALLELPPTQAVELLHSPDCPAADREPPCHVSTNQKPYLLCSYGGNLTFPSNAALIERAAKFSVFAGENSPPEDAARLAPVAVSGVVKNEPQETDSNPSSTQGCVSDPAVDTKNQKTAKRKEREKKARREKINARMKLLQELVPGCNKISGTALVLDEIINHVQSLQRQVEFLSMKLAAVNPRIDFSLDSLLATDGSSVMDSNLTSMASPVMWPENLANGNRQHFQQHWQFDAFHQPLWGREEANHNFMTPEHSLLSYDSSANSASLQSNQLKMEL.

>Aradu.1124E.1 genotype-assembly-annot=V14167.a1.M1

MGNLYDNRLTVSVDELYFNAENSSINKRGRMGRRNYDADDTVVYKSKNLETERRRRQKLSDRLLMLRSLMNKATIIEDAITYIKNLKDKVDSLTRELQEIEATSETTLVEPKTNETNNGEAMKEWGIKEEVRVTNIDGKKLWLKMIIEKKKGRFTQLMDAIHSFGIELIDTNVTTMKGALLITASIQGMDGEALVVQQTKELMLDIINSTHSY.

>Aradu.Z6I4Q.1 genotype-assembly-annot=V14167.a1.M1

MPPAPSSSSAISSFVSPKKRKSESHKVVVAQQSHNKKDKRVKVSAEEESSKMSEQITNKDEVASAENSIKGSTSEPHNNKPDYIHVRARRGQATDSHSLAERVRREKISERMKYLQDLVPGCNKITGKAGMLDEIINYVQSLQRQFLSMKLAAVNPRLELNVDDLFAKEVFPACAPNFESIGMGSSEMTNNPAAYLDFNSVQQLVSCSNGLINNIGMNMTPSPDMGLRKTIAAPLSVSLPETFLDSSSCFNQVLPPSIWEGEFQIQNLYNVAFDQPRTSSFPPSHPFTGTYKL.

>Aradu.0K58L.1 genotype-assembly-annot=V14167.a1.M1

MDHHQQQQHQQGSEPSTTTKVERKIIERNRRNKMKDLYSKLNSLLPSYSPTEVLPLPDQIDEAVKYIKSLEKKVKMAKEKKERLLKGRNNKKRSRGFDEKNITKSPMIDVHEMGNSLLQVVLTCGFENKFLFNEIIRILHEEKVEIVSVNSSSQPGDSMIHVLHAEIPSAFQQFGAARVSERLKRFVNGSISDVDIDPGFWDDFEIDTDIWELPDLSSVVSKGFPNPL.

>Aradu.TN75P.1 genotype-assembly-annot=V14167.a1.M1

MMKKEEDQGQCSPQAIHNTIQSYQEQLFLQQQMQHHNPNGTDLYIGSGGSGRGLVFPPEASPIMQPPWSMPPVHPFNQAGPVHPVSHHNDNNNNNHDPFLVPPPPSSPYASFFNRRVPSLQFAYEGPSSEHLRIISDSLVGPMVHPGSVGPFGLQAELGKMTAQEIMEAKALAASKSHSEAERRRRERINNHLAKLRSLLPNTTKTDKASLLAEVIQHVKELKRQTSLIAETNPVPTESDELTVDTTDEDGRFVIKASLCCEDRSDLLPDLIKTLKELKLRTLKAEITTLGGRMKNVLFITADEEDYSSSNAGSGGGEQSLQLGHYCISSIQEALRAVMEKGGGDESASGNVKRQRTNINILEQRSL.

>Aradu.LXV06.1 genotype-assembly-annot=V14167.a1.M1

MVYSFYNSNDFSSPGYDVSRLISPSLHDYGASYGVLEPSHALILESEKIEQVNNGSEDARVGKNYENSDAKALAALKNHSEAERRRRERINGHLATLRGLVSSTDKMDKATILAKVVSQVKELQRNAMEASKGLLIPTDFDEVEVEPYEDHRSMSYKASICCDYRPEILFDLRHTLDALELQLVRAETSTLEGRMKNVFVYRCCKGKGDNNNTEACQALARNVQKALSRVIDKASNALEYSLRASLPCKRRRICLLEN

>Aradu.752ZV.1 genotype-assembly-annot=V14167.a1.M1

MNPWNPCDDLYFQISSSSADYSSLPIIQTSPSAVEDHHHHQILVHGYEYDPAMVDASASVPPCPNQKGKGKGKQPKELTPNNNALGAEDGDENKKWMHREIEKQRRQEMSRLCTTFRTLLPFEYIKGKRSTSDHMHEGVKYIKHLQNRVEKLQAKRDELVKLINLRPKSGSSSSSSTQHDHHHHYHQTFVIVEPFSGGVLIKCSYSFRNYAFPLSGILDIVLRQGLNVVDCTSITTDDHRFIHTIRSEDPQVMTTETDYTELQRKLKEAISSSSN.

>Aradu.M594W.1 genotype-assembly-annot=V14167.a1.M1

MGEPCHNYWYSDMGIQDDDIFNQRYKINSSLIVDEDHIIREIMDHHHHQPAFSSESDNSHSPTNNNQIKGGGSGNTSSFVFNNNNQHAPLLLDMKASTSTSSPRSYILSFHDSTVIATTAAAAATPPPPLPSLETYNNNEKRPYQHIEVALENQAKKVRSSSETLDHIMTERKRRRELTERFIALSATIPGLKKIDKSTILSEAISHVKQLKQRVKELEEQKKKISVESVSFIIRKSHLMNGTNNKDDEKGAINNKAATSEALLPTVEARVLKNDVLIRIHCMKQSGIMLKILRHLKSFDLSAISNSVLPFGNSTLDITIIAQIGDKFNVTMNDLVKNLRLSILESPNDDEEPHNSN.

>Aradu.LLR91.1 genotype-assembly-annot=V14167.a1.M1

MSDSTVLEWLRPFVEAKAWDFVVVWKYGDDPTSFIEWLGCCCSGSCGESIEDAKKLKDEEMDEKQNDIDSSSICKDAHFQHPIRTKASTKMAYSWRGNIFGLDHDTLLEIGTQVLIPVVGGLVELFTTKLVPKDNNILEFIRAHCYVSLKQEAICAQHHTDVNFSENLSSEEQYTQSSPQLASTLTDGVHLLAANWCKSDPYIEEPSSGSNPSSEYTSFDSKFVCLTHHEYLGESVKLSPTCKTERPKYNETSGKQQGTLSSYCGNGKRNKTKSVRMDRASILADAIDYIKELHGQVEDLKDEVRNLEVEDCEKNTPQSIMPTSKEQGENRTLLAELNQSSSNSTKQIEMKMQTEVNHISRTEFLLKVCWEQKPGGFSRLMEAINSFGFQVETANMTTIDGKAQIILTVEAAKEGIHPTKLKDFLTEQTG.

>Aradu.C4XJQ.1 genotype-assembly-annot=V14167.a1.M1

MIMKPFLDIDPSMDLINQFIGLNTTTTTTTHMNMCDNLFGSHHHHHHHQDDFPGNLEESFHQNHHHHVNNNNASSVVVPVISSSLLNYYPSENEEIHEGKKRKATTSGNSTPAASHHSESKIIKNNEVVHVRARRGQATDSHSIAERVRRGKINEKLRCLQNIVPGCYKTMGMAVMLDEIINYVQSLQHQVEFLSLKLTAASTFYDFNSETDALETMQRARASEAKELGKYGKEGHGGVSCYQPNWPLV.

>Aradu.47FME.1 genotype-assembly-annot=V14167.a1.M1

MAEFTADLNSFRPSFPFLDINTMELTNGFKGVMNVNSHILNNNSSLHMQDFFMPFNTSHSFTTSPESEFSGLVHYVNHSNLPSSLTISSADNEIQQGKKRKAVDTPDTMSANSTPAVSESGSNTKNNYGRGKRGKKNETEEKKSKEVVHVRARRGQATDTHSLAERVRRGKINEKLKFLQNIVPGCYKTMGMAIMLEEIINYVQSLQHQVEFLSMKLNAASAFYDFNSQTDAFETVLRARASEAKELVKNEREEHGIQPTWPYCF.

>Aradu.Q2I1J.1 genotype-assembly-annot=V14167.a1.M1

MEEEEEENDDTIIMNNYSLNEEQEFLMKIFNGRPDFSSPESSEHYYYSSPNNNNNNSYNNNDPNNCDTSPNNNNSSVSFEDTRVQKSKSSNSSSSTYLLSFEPNSFKGRSRGDDGFELFEASMNKNDEGGTTKKRKGKTVDHIIAERKRRQDLTRSIIQLSATIPGLKKMDKAHVIRESLSYIKILQDRVKELENQIKDRRVDSAIFIGRSQDSLSTDKSTISCEITSDNNNGGGGFNESSLEIEAKVMEKEVLIRIQCEKQKNNIIMLKIHAFLDKLHLSIASNSVIPFGTSTLVIITIVAEMDNGGKFSMTMDELVKSLREDLMETNNNAW.

>Aradu.0DZ84.1 genotype-assembly-annot=V14167.a1.M1

MMPLHELYRKAKEKLDCSKEINSTSATDHSTAPESDFYELVWENGQISMQGQSSSRGRKSPTCKSLTSHCPKGLQHRDVVGYGNGTNNVNMMRMGKFGDSESGLNEIRMPAPSAEDEDVIHWLNYGMDESLPHDYGSDFIHELSGVTMHEIPPLNNLSLLDKRSNSNQLLRDSHKNYARHAFGSEQGILNKDFSVMAKGEIEIPRPKPSTSQFCQPSSYQCQGSFASVRSKASEITENNGSNPTHQVPCG ELAQIFPSASSGFSGLKLDKQDQVMCSSSSTIMNFSHFARPAAIVKANLQ NIGLSSSRSDGIENKNNDASATASNPPELTKAGFSGEHPKQSAVHELKIVEPSKADLKQLEPSKADLKQLEPKSLEVNATVLRQSDPARKEDVSKIYQSSNLLLCESTNKGEEAVVKNMEPAVASSSICSGNGAERISGNPNQSLKRKRQETEDSECHSEDVEEESVGVKKAGPTRGVKRSRSAEVHNLSERVSEGHMPIVGEETGSMRRCVHCKISYQTAIRWTKLQCWTRQSSISKHFNSKFKLCQWELVCICLRMGMPDMNGGPSRFPMIQVPQMHRTHPPAAPMPGPSALHGMGRSNPPVFGLPSQGLPIPTMPRAPMFSYPGEPVANSPALQPNACGTAGLTETENPASASNQKDPMPIMQNTNGCNSTSQTTKQCEAAAAGRIEPSASQANDGR AVDATRKDNLVTDKFD.

>Araip.807VL.1 genotype-assembly-annot=K30076.a1.M1

MADDEDFQGAAGICGENWWRSIDSTRSVFPPLTSSSSPCSVAPPTDTRTWPSSDDHHHCFMDLKAPACDIISNNNNNNYHMGFHDAQEKPHNNKTSTNESQSGSILIDSTFQMMGFGLSSPNWDHQSLLENNNWSRKNFGTQQQEVSSTMDVFKRMNNQEFSSSTLIQTLYDPQRSSSSSSLFTNRSMSYSSSSTVNYHHASDEVSLSPITWPKLSSSSSSSSSSLSLLPPMAKHPDNNNIGLHFSNNNNTPFWNASSDAFPFEEDNKPNSPIILLNKLKSEELCLESVKKNNNVCSSPPPFKRPRIETPPPLPTFKVRKEKLGDRVTALQQLVSPFGKTDTASVLHEAIEYIKFLHDQVSVLSTPYMKSGASIQPNQECNDWKESSEGAKGDLRSRGLCLVPISSTFPVTTEVSADFWTPTLSLGGALLR

>Araip.DHX2R.1 genotype-assembly-annot=K30076.a1.M1

MELSQLGFLEELMLAPRKDTWTSSTSNDVFSPSSWNYFDPFLDTPSSSSFPPSAFSPPPPDQRFIIDCPFTTSYPFPDDAFTMPDLDSSYTTKYEYDDAPPPPLPHNHQDVDDDNDNNNNNNNPSVLEDEDAGYLGCKVEEQAVVDVQVHVPVVFNMGMCGIGEKKNKSKKLEGQPSKNLMAERRRRKRLNDRLSMLRSIVPKISKMDRTSILGDTIDYMKELLERINKLQEEEVEEGTSQQNLLGVSKELKPNEAMVRNSPKFDVERRDKDTRISICCATKPGLLLSTVNTLEALGLEIQQCVISSFNDFSMQASCSEVAEQRNCVDPEEIKQALFRNAGYGGRCL.

>Araip.2LX3X.1 genotype-assembly-annot=K30076.a1.M1

MPLYELYRLSREKLDEEINGTRATDQSQSSSLSVDSTFMVAFGVVASLPCDFDFSRPEKDFFELIWENGNILTTQGQSSRAKKSPPRRSLPSHCLPSHSPKGRDRDAGYVNNSRVGKSGDLDTGLNEISMSVPSTEVDLGHDDDVIPWLDYTMDGSLQNEYGSNFLHELSGVTDQDLPSNHFSLVDKSSGNQVFRDSHKNSAEQSNFSSVSSTGVDETTRPKASTVESYLPSSFISVRPRVSGVTANDTSNAMLHPPVTEIPSSSSDFSSLKMQKQDQVIPSNGSSVMNFSHFARPAAIVRANLQNIGLKSVSSSTRSENVESMNKGAVVPSSNLPESTLADSCSECPKVLMGNNEKAVEQSRDDLKLLESKSLEQNIAGSKQLDPTCKEKAIKIDQTSNRALGETATNTQIAVERSTELVVASSSVGSGNCADRGSDDPIQNLKRKNRDTEDSEWHSDDVEEESVGVKKAAAGRGASGSKRSRAAEVHNLSERRRRDRINEKMRALQELIPNCNKVDKASMLDEAIEYLKTLQLQVQMMSMGAGLYMHPMMLPHGMQHMHAPHLAPFSPMAYGMQMGLGMGYGMAMPDMNGVSSRFPMVQVPQMQGTHVPVAQMSGATAMVRSNTQGFGVPGQGFPLPLPRAPLFPFSGGPVMNSSSALGLRPCGATGLSQTADLASTSGLKDPSPNTDSLVKQSTGGGGCDSTSQMPTQCEAAPTVGFEQSPMVHNSSHTSEANDSGTLNPDKEDNNLVTGYDD.

>Araip.2U2B9.1 genotype-assembly-annot=K30076.a1.M1

MSQCVPSWEVEDTNPPPPRPSLRSNSNSTIPDVPMLDYEVAELTWENGQLSMHGLGFPRVPTKSSAVTTTANKYTWEKPRASGTLESIVNQATTTPQNAKSTAVHGFGGVYENLLVPWVDPHRVPATVISPGTSNTVTMDALVPCSNRVEEQRTPQVMDSISGGLGACMAGRTTRMGAGDAKDGGALEKRAAMVRRVPAPTVSAHEMSSRDQSVSGSATFGRESRHVTLDTCEREFGVGFTTSTSMGSPENTSSAKQCTKTNTIDDHDSVCHSRSMEEKKKETGKSSVSTKRSRAAAIHNQSERKRRDKINQRMKTLQKLVPNSSKTDKASMLDEVIEYLKQLQAQVQMVNRINMSSMMLPMTMQQQLQMSMMAPMGMGMGMGMGMGMAGMGMGMDMNTLSRTNIPPGIPPVLHPSPFMPMPSWDAAATATAAAATGAADRLQGPTPSAMPDHLSSFFGCPSQVSKCSKIILYLLCKIISLEV.

>Araip.L4GEP.1 genotype-assembly-annot=K30076.a1.M1

MMNNNSTIPGWNFESDACVINQKSPIGLDQDLVELLWKNGQVVLSSQTQRKPVQKSDASSTLRNGVHCGNSSSNLSQQEDETASWIQYQVEDHPMGQELCSSNLLSQLTPCEVEAYKPIKQLEEPKFAKFATPNANLSPQQQNNNIKPINPMPAPRFQVAPDSSNQKTNDFVGGSSSSQKVPKFSHHFSAPAQQKFREKVNGNNNVLQSEGRECSVMTVGSSHCGSNNIIPQEQDTTFSEAVKDCVQRSVPNWSNNKGKSSEMIEATVTSSSGGSGSSLGKTTCSLSTGNHLSLKRKITDADESEEQSEATEPKSGVGNKTSQRSGSSRRNRAAEVHNLSERRRRDRINEKMRTLQQLIPNSNKTDKASMLEEAIEYLKSLQLQLQVMWMGAGMTPVMFPGIQHYMSQMSAMGMAAASQPSLSSMQNPMQIPRMPLDQSNMPSTVSVPHHQTPSNQALMCQNPVLGAFNYQNHQMQNPCLADQYARYMAYHHLMQTASQPINVFGHGSQAVQQSQTMMPPSNNNGTMNVAPNFEDAITAKMGTSTFQQLNNN.

>Araip.K6RXL.1 genotype-assembly-annot=K30076.a1.M1

MGPFGPFWTSFSWALLGQYTSILNSEIKAVERDIIPFQLHPQCIKGCVQRHQISPCFSPFPVQLYQYTLIVTSSSSIHSDFRSKPNVQVFVDKSEFRNDDKHVTAVAAAAFSIHSIEQAAAANLISRPQSMRRKNHSTLSERPTYGGDTSVKRSSFGEDGRRKEGSVPLRGSSDDISSKRTVPQTQGHQKQIGIPIQHNNKANAEAWEKAKEKKIHAKMHMEKKKNELEKRRIVTTQHYNNKIASIDKVAQGALTQLEDKRRKELSRATEKANKIRKTGKGSLYPILFERTSRHHKANNYDEIAELTWENGQISMHGLSGLDPTSQKKPIWVNRAHDTLESIVQQATCNNKKSKLTIKDNHAYVDVPTTTSSIVASSGEHHQMVPTLSRKRSHSSYSDQQQHRRDVNYVSINNTKKMLLEDHNTNNRKCGVATASVTFCKDNNDITTMMTCPSLDSGPRSFKNDKILEEDSACQSGSEVRNNENDRDGGKGETGQSKSSSVVRRNRTAAVHNQSERRRRDRINQKMKALQRLVPNANKTDKASMLDEVIKYLKQLQAQIEMMMSVSMPQMIMQQQLQMSMLARSMANNAPNPIRPLFPQLIQPTTTIGAANTASAPMFLAPSLMMPSSTNASFPLPSASYGTPTFAQIRPST.

>Araip.44BHE.1 genotype-assembly-annot=K30076.a1.M1

MENYYYSGWPQSQCNNSAPNSNNNPSFSVPTQIVPHAYDSAFASLQFQYPWPLAFEGFPEDRAASASKSHSQAEKRRRDRINAQLATLRKLIPKSDKMDKAALLGSVVDHVKDLKRKAMDVSKSITVPSETDEVTIIECDPDQDESYAKVKILKHNIVISVCCDDRPELFSELIQVLKGLSLTAVKADIASVGGRIKSILVLCSKENEEGGDGVCLNTLKQSLKSAVNKIASSSMTSNCPTRSKRQRFFLPSHFIQ.

>Araip.IBY8N.1 genotype-assembly-annot=K30076.a1.M1

MGDHMYNYDKNLSSSSSSQDEISLFLRQILLRSSQPHQDSPPPPQQQQQLPSSSSSSCFPSTSQLQDGKISALGSTRAGFLSASSMKGHGGNSAPNVSSSSVGLSANDTDDYDCESEEGVEALAEDVPAKPVPSRSSSKRSRAAEVHNLSEKRRRSRINEKMKALQNLIPNSNKTDKASMLDEAIEYLKQLQLQVQMLSMRNGLSLHPMSFPEGLQPLQFSRMSMEFSEENRAIPVNMTESLPLHQGNPLPYASLPNKPTMPNQPSVTYPSYLNNSEAPFGLEPPILAHIKHLQPGRFSEAHREDTLQQQQSNASHSDTDPLGGSQAVKELETGTMPTTSLSFDLQTCEPKDNNPLQICIAGRDQSSSVIIRNS.

>Araip.MHR6K.1 genotype-assembly-annot=K30076.a1.M1

MPSCSRVQSMLQAAVQSLHWTYTIFWQLSPQQRVLRWSDGFYNGEIKIRRTVEAIEANKSSSNNEEASLQRSNQLRDLYDLLLSDRHGDHVFAVDPPATPRPSTALSPEDLSESEWYYLMCVSFSFAPGVGLPGKAYARRQHVWLQGANETDTKTFSRAILAKSAHVKVKEDINVIKHVKSFFIGDHHRPQPKHALSENSASNPITEEAQLLLDSETATLPQSKLKEPVVAEDGSRAGSPDDGSNRLNSAGLELLPPESAASLELLHGDEYDNNSEAETSHLCTEQEGHTHYSKTVSTILEGSSGHRPDAVCGLLSFQSAFATWSSRAYNCHQVTVVENPQRLLKNILFRVPYLHRKQNRHNSAETLEQLERTIMPSPQDDLISSHVAAERRRREKLNEKFITLRSLVPYVTKMDKASILGDTIEYVKQLHEKVKKLEEEDQRRRSGKKRKERAVEVSVIEGEILLEVECMNREGLLLDLMRTLKEVGIEVRGVKSSFKGDDERVFVVELRGKVKENGKRNNNNKKNKKISIVEVKKVLHNIINPHHHPL.

>Araip.RAG9J.1 genotype-assembly-annot=K30076.a1.M1

MCLPESERDMALEAVLYTNNIQQPLPSSYAINDTNKDLDFFNFNNEEDQHQFIDTGLHYLLSPPPPPPEETETTMDTTSTCSERPKRRRGKTRKNKEEIENQRMTHIAVERNRRKQMNDYLSLLRNLMPDSYVQRGDQASIIGGAINYVKVLEQKLQFLAAHKEITTTTTDEDEGSDLTNNKTGTMPFSEFFGFPQVTGNADIEVTMVESHANLKIRSKKKPKQLLKMVSNLQAMRLTILHLNVTTTNNDIVLYSLSVKVEDDCKLGSVDEIATAVCQMMENIQHESVE.

>Araip.B5XPZ.1 genotype-assembly-annot=K30076.a1.M1

MTKGVGNYSGSDELSLSTMNRFNNQISFSSSRSPSSSATATNNNNRNGGLFFPNYGSWNETSYKRDDQNRIHKLIFYANQNEEFGNNKVVHTLSHQLSFPKAESEMFAMENMIHFPLSDSVPCKIRAKRGCATHPRSIAERVRRTRISERMKLLEELVPNMDKQASTADMLDMAVGYIKDLQKQFKNLSDKRAKCKCIRMQKSDSSKKF.

>Araip.YR6Y7.1 genotype-assembly-annot=K30076.a1.M1

MSMILALSSPTSMFPNNMEWPLELEEEELSHSHEYFNSEEYPLLSSSMAKKFNHNASERHRRKKINALYSSLRSILPVADQTKKMSIPATISRVLKYIPELQQQVEELIKKKEELLLRISRQGDINNDDAAMNKKGHHHHNSSAFLVSSSRISDSEVSIHMISSYGIQKCQVSEILVCLENHYALQLLNASSFDTFGGRLFYNLHFQMEMAHTLDTEVLSEKILSICEKHQRI.YLCNVNII.IFSFFSHSILID.

>Araip.AR8NT.1 genotype-assembly-annot=K30076.a1.M1

MDVDIFKTSTASDSMDMMAMMMQQMEKLPPPELSEPPFYTTYPSQTDFHNNNNVVPQVEPNLINPTFIFQQQHSMAPPPVVTSNNDDIITSSFPYPPPPPPPSCSDSSNKKNSMAAMREMIFRVAVMQPVHIDPESIKPPKRRNVKISKDPQSVAARHRRERISERIRILQRLVPGGTKMDTASMLDEAIHYVKFLKKQVQTLEQAGGGRTCNNNGFTGFSSSSSSINASNYPAMVKGCHQPYPPMLMGSSASKQLLSSMMMMSLINSVVFCSVVSVL.

>Araip.A522U.1 genotype-assembly-annot=K30076.a1.M1

MESSSPSWLHDLEMEDDYNNFFPKEYSMNNSIDDYDEDLFFAHDIGNSIMFESPSNNNNYSSVDETSFDDERPSKFLKTSTNNYTTLNTKTKLKEVVNGSSKNQNLVTKSSSNGSKRSPSNAQDHIIAERKRREKISQSFIALAALIDKVTVLENAIKYVKDLKTRLTTLEQENNEKIKEIAEEPSVIVLNKRSPHDNESDDESVIVDDASNHDSSSLLHQVEARVSGQQMGEGYNLTVKELVKSIRVETLKFILS.

>Araip.03FDB.1 genotype-assembly-annot=K30076.a1.M1

MDLSFCFSCFLFVCYSISLLLVLVKGIISSILKHFAFGSSKKDFYYHLLPSGDKNMNMMMMQNLTERLRPLVGLNGWDYCVYWKLSEDQRFVEWLGCCCGGTDQNNNVGEDIHIFPCRDTMFSHPRTNHCYLLSQLPTSISMIDSGYETIGTQVLIPVPGGLVELFVTKQVPEDHQVIDFVTAQCIVLVEQEAANNSTSFNMQSNVVGDDNNNNNNNNNNNNENQVMMNNNNNNNQFVLTTAPETSPHDEIPITLCSSPLNFMQQFRTMNKNNDNNGVAAAFSEEYQGSIFLHENQNNNNQPMKAAAMEEEEEEEQQVGTKENNNNKNEGGVVGVGRSDSMSDCSDQNEEEEDGKYRRRNGKGNQSKNLVAERKRRKKLNDRLYNLRSLVPRISKLDRASILGDAIEFVKDLQKQVKDLQDELEDNNNNSDTTAVDHHDSSNQFLDHFGASYVIPNHKHMQQEAVDATTLTMDKQHSQQMEVLARHTKVKINIVQPQVEVAVIDGKEYFVKVFCEHRAGGFVKLMEALNTLGMDVVHATVTSHKGLVSNVFKVEKKDSDMVEAEDVRDSLLELTRNPSCRVWSNNNTKNNNPTSENSNGVGVGRDHHHHHHHHMPAYHPHPFHT

>Araip.V1VB7.1 genotype-assembly-annot=K30076.a1.M1

MDHNNSDLFQLLGAGAGGGYYPAAAMLMMDHHHQHQVSSDDNMSAIETQASHHQQQDRALAALRNHKEAEKRRRERINSHLARLRTLLPCNSKTDKASLLAKVVQRVKELKQQTSEIITEFETLPSETDEISVLSTADGGHGGLIFKASLCCEDRSDLIPELIEILKSLHLKTLRAEMATLGGRTRNVLVVAADKEHNSIESIHFLQNSLRSLLERSGSADRSKRRRAMDRRFTPASS.

>Araip.A8YH6.1 genotype-assembly-annot=K30076.a1.M1

MAEEFQATICGDNYWWNSNRSLFSLMGGSMTCSVAAANDSANNYGSFWQGSDFMDFKGATTKSSFKEEDTDKCSNNESSISAAVAGGTVGSIFINSTSQMMSYGLSSSPSSPWNNPSLFGCNERSENNFQSVVLQEETGLDPSSNNSQQIQQQQEFSSSMGGGFTVESSLSASHDYPLMQAGLFGSTDSSSSLFTTNQPTLPELSPIWSTVSSFTKPSSFMEPKQLNGLQFSNNTPFCNASTEETLYNIRSDIFSSSSSQPQLYQTSPTTFDNSKPNNALTSTPLNKLKREESPEKASSSVCSKNVSGRVFKKARIETPSPLPTFKVRKEKLGDRITALQQLVSPFGKTDTASVLHEAIEYIKFLHDQVNVLTAPYMKNGSSIQDQQGCDYEKDSKCLTQDLSSRGLCLVPVASTFPMATETTADFWTPTFGDSLIGNVAGSHQ.

>Araip.438GZ.1 genotype-assembly-annot=K30076.a1.M1

MLSRKFLTKNTTRRGTIAPAENDRLQTLVFKFGVAKSIHHAMFCDNRRFVDFRCATPASSRFANNNNNNDALEGDVDFSIVTEKQRREQLNGKYKILRSVIPSPTKMDRASVVGDAIEYNKGAAQNGLNQNSTLQLSRGFSVGKSPGSKPGVAVVVERVRIHGLSRFRNLSKFAHSMKVKVLPADPNIRIPNIEICFHRAILCVLFRNASLAIGMCSQGQWEKVTKGSWVRSMSPFDHKLLDIRTASSTLASFLSKSLAFYYSSAMAVGIILVILMILYQTEHCGWGVEAAETIYKDEFIIEYIGEGVGSESERMVLGVA

>Araip.6S5NP.1 genotype-assembly-annot=K30076.a1.M1

MGSENQTPMTETSKNRSSGKTNQGKVPKRIHKAEREKMKREHLNELFLDLANVLDLNEQNNGKASILNETARLLKDLLCQIESLKKENSSLLSESHYVTMEKNELKEETASLETQIEKLQVEIQARVTQSKPDLNAPPHVEIEPPEQANFTGQSLQFPPMEPNLQQGPAVFVVPYRPGFQAAFSAPTVAEVTPKPSSAISKPHARYPTPADSWPSLLLGQQPTSS.

>Araip.QB13B.1 genotype-assembly-annot=K30076.a1.M1

MNLWTDDNSSVMEAFMTSSDLSSLWPPPPPPPQSASSAAVFNQDTLQQRLQALIEGARESWTYAIFWQSSYDYPSSASTAVLGWGDGYYKGDEDKGKAKSTKTTTPAEQDHRKKVLRELNSLISGSAAAPSDDVEEEVTDTEWFFLVSMTQSFVSGTGLPGQAFYHSSPVWLTGPDRLAGSSCERARQGQVFGLQTLVCIPSSNGVVELGSTEMIFQNPDLMNKVRILFNFNSNSIDVGSSWPLTGSTTTTAADQGENDPSSLWLNDSEIRDSVTTVTTVTTTTPASVISVNPASSALAETPSSVHLPNNNNNTHAGGASQSQNRSFFSRELNFSEFGFDGSNAVKTGNGQHHSLKPESGEILSFGESKRSSYGGGVGGGGGGGNANFFSGQSQFVAAAEDNNGKKRKSPNSRGSNNDDGMLSFTSAVILPPSNMKSSGGGGDSDHSDLEASVVKENESSRVVEPEKRPRKRGRKPANGREEPLNHVEAERQRREKLNQRFYALRAVVPNVSKMDKASLLGDAISYITELKSKLQTLESDKDGLEKQLDSLKKDLDKVKKEASSAPPPPLPDKELRMSSNNLVGGGKLIDLDIDVKIIGWDAMIRIQCSKKNHPAARLMAALMELDLDVHHASVSVVNDLMIQQATVKMGSRFYTQEQLRSALTSKVGGDVR.

>Araip.UC4CT.1 genotype-assembly-annot=K30076.a1.M1

MTDYRSTPTMNLWNDENSSVMEAFMSSSDLSSLWLPPPQSAASTSTSAAPPPPQQQQPPPPPPFNQETLQHRLQALIEGARESWTYAIFWQCSYDYSNGLTLLGWGDGYYKGEEDKGKAIKATSFEQQQHRKKVLRELNSLISGPSASGDEAVDEEVTDTEWFFLISMTQHFVSGTGLPGQAFFNSNPVWVAGPERLLGSGCDRARQGQSFGIQTIVCIPSANGVVELGSTELIYQNPDLMSKVKVLFNFSNSSGDVAGSWPLSSADQGENDPSSLWLNDPSSSAGGIEIRDSVNTAATTTVANNKTVPVVSNPGSSSVVTEAPKNPQQNQGFVLKELNFSSSLKPESGEILSFGENKKGSYGVNSNNGGNNLFSVQSQSQFVSDENKKRRSPTSRSSIEDGILSFTSGVILPASNAKSGGGGGGDSDHSDLEASVVKEAVEPEKRPRKRGRKPANGREEPLNHVEAERQRREKLNQRFYALRAVVPNVSKMDKASLLGDAISYINELKSKLQGLESEKGELSKQLEDSKKELELASKNSGAAQAAPPPLPAPPDKEQRSRAGVNGKLIDLEIEVKIIGWDAMIRIQCSKKNHPAARLMAALKDLDLEVHHASVSVVNDLMIQQATVNMGSRFYTQEQLMLALSSKVGGGGDAK.

>Araip.LC4KN.1 genotype-assembly-annot=K30076.a1.M1

MISTEESWTTWLCDLEEEDYSFINGIIADPNNYSNSQMSNNDNERPSKLLKSTTPTPTRRRRTGSAGTGRSPQHAHDHIIAERMRREKISQQFIALSALIPGLKKMDKATVLGDAIKYVKQLQEQVKVLETESKRKSAESVVYVEKSEVCGEEDVSDTWSNSGGDGNSSYEVSKAVSRSVLPEVEARVSEKNVLIRIHCEKHKGVLMHILKLIDKLHLSVLNTTSLPFGTSIVDITVTAEMDDKFSLSAKELARNIRVGLLQSM.

>Araip.D3S94.1 genotype-assembly-annot=K30076.a1.M1

MLQQNLQFLLQSQPSWWVYAIFWSTTKDDNGNLYLAWGEGHFQGNTTKHTKTQQQHNQNDAEWFYVMSLTRTFPIANSSSSSLPGKAFALGSVLWLNSKEELQFYNCERAKEAHAHGIETLICIPTSNGVVEMGSYNTIPQNWNLINHVKSVFEDSMVNNNNNNNNHCSNSRKQADVTVPFNKESKDSYAESEHSDSDCPFLKTENTENKNKLEAPKSKRGRKPVLNRETPVNHVEAERQRREKLNHRFYALRAVVPNVSRMDKASLLSDAVAYINELKAKIKDLESEKDNYQRKKKVKLESGDTMDNQSTVTNSSTVVDQEKCSNGVVAEVDVKIIGDDAMVRVQSENVNHPGARLMGALRDMEFQVHHASMTCVNELMLQDVVGKVPSGILRSEEGIRSAILMRLDHIN.

>Araip.LGM59.1 genotype-assembly-annot=K30076.a1.M1

MEDCEDYKNYWETNMFLQTQELDSWQLDEAFSGGYYDSSSPDGGASSAASKNIVSERNRRKKLNERLFALRAVVPNITKMDKASIIKDAIDYIEHLHEQERRIQDEIMELESEKLKNPIIAAAGDNDFEQDLPVLLRSKKKRPDNLLFDTSASSSSRNNTNYFPIELIDLRVTYKGEKIFVVSLTCSKRTDTMVKLCEVFESLKLKIITANITSFSGTLSETLFVEADEEEKDHLLIRIQTAIAALNDPQSPMSI.

>Araip.28KZQ.1 genotype-assembly-annot=K30076.a1.M1

MNQLGSRSQASPSSTNKKKIERRVIEKNRRNHMKMLYSKLNSLLPRPKEPLPLPDQVDKAINYIKSLEEKVKMAKEKRERSLGMEIRRKRTRGDCSGVDRQKPPQLEVHEIGSCVEIEMTCGLDTQFIFYEIIRILNEENIDVKSVNSSLIENSMYHILRAEISPSLLQFGVTKVSERLKRFVNEPTSEIELQSDYQLLDFEIDTDELAELLDFLEEK.

>Araip.97C7E.1 genotype-assembly-annot=K30076.a1.M1

EIPFCLVWFWDMEMEGFAASSERSSGVGGGYASSSSLVLDRVKGELVEAPVKLERKGVFPERTIEALKNHSEAERRRRARINAHLDTLRTVIPAANKMDKASLLAEVIRHLKELKTKAAQACQGLMIPKDNDELRVEEQEGGLNGFPYSIRASLCCEYKPGLLSDIRQALDALHLMIISAEIATLGGRMKNVFVIISCEEQSFEDAEYRQFLAGSVHQALRSVLDRFSVSQDILESRKRRRISIFSSSSLEDFL.

>Araip.UYW0K.1 genotype-assembly-annot=K30076.a1.M1

MEATSMTHQLLKGFCDGTQWKYAVFWKLNHHFPMTLTWEDGYYGYQKTNEAAESMLDDINFKFPAEVYSSSGESIDDPGDYSAGLLVAEMSHHKYSLGEGVVGKVAFTGNHCWVSEDILTHGLDANLMPECPDEWLLQFASGIKTIVLIPVLPQGVLQFGSFQAVAEDLEFVTNIKEKFHSIHLLAANTTALNYGVDCQDWSFSDPTNTFMDILDESSNITNSTIKTDVLARIVPSVNASTMLNPAMLSQVETKDNLEEEIWPTSPWVNDVGVFQEISNGLGLYSGKTEQQFGSNATGYEDIKDLNGFLAFPSESELHKVLGSAAPHRKTRNSMSKHTSVVDTYSNSTFISNNKEHGDIESLEHPEDVDPKYLLDAVYGDLFSACNDTSSISNSFRSPISKKMPFTGLIHPRNSCEESSLVMKDVKGDLKAAVRVKGRDAITSPSFDGNSSLLTDEPQEEKVYSHLQSTNGPKISSTYKKRARPGTQKSRPRDRQLIMDRMKELRELVPDGGKCSIDNLLERTIKHMLYLRKITSQAEKLKRFAHKEVPKCSKQKINYNQSGRSCAYDLESEQAWPIVIEDLECSGHMLIEMVCNEHGFFLEIAQVIRKLELTILKGVLENRSSAAWACFIVEAPRGFHRMDVLCPLLHLLQRRKNPVPYKS.

>Araip.AK8SS.1 genotype-assembly-annot=K30076.a1.M1

MASSWSPKHEKMQKSLSTQLAVAVRSVQWSYGIFWAPSTTQEGVLEWREGYYNGDIKTMKTVQTTMEIKNDKIGVQRSEQLKELYKFLLVGESDPQTKRPSAALSPEDLSDSEWYYLVCMSFVFYPNQSLPGRALEIGETIWLCNAQYADSKVFSRSLLAKSASIQQKEILSDYYIVAVTKISMFILCFKPVFQTVVCFPYLGGVIEIGTTELVLEDPNLIQHVKACLLEFSKPICSDKSSSALNKPDDDKLQPCSNGEHVLVDTKVLEYSCSPAEEIKLDQDPIKELQQDSNMDSPDGCSNGCEHNCRVGESMVEGINNGGPSSQVHFMDDDDALSNGAPDSLSSCDCISEASENRGKASKDVQLIRGIQSFNHLNRSSLDAEATDEHLSYTRILSALLGRSSAFKQNPYASNSNCKSSFVKWKKGGICEQNRLRLEQSLLKKTLFTVPLMHRSFSSIESMKENETKEWTNRLENADDKFRENVLSDKIRETGNFKILKSSIPHPISEVEKISILGDTIKYLKELETRVEELESYMDMSDSEARTKRKCPDMLEQISDNYGARKVYNKGMRKPWMMNKRKACDIDEKDREEIDRFVVSEEDNNKPLDVKVNMKEQEVLIEMKCPYREYILHDIMDAINNLHLDAHSVESSTTEGVLTLTLKSKFRGTATAPLGMIKEALWKVSGNL.

>Araip.Z1VRT.1 genotype-assembly-annot=K30076.a1.M1

MLPFGGRFYGFESWLDQPHQHDSSPSDGFFVPEAPFKATDLLFDGAGRSSKLDNNRKSTEACKSHREAERRRRQRINAHLSTLRSLLPNTAKADKASLLAEVVKHVKRLKKEADDVARDPSKPDAEAEPWPFPGESDEATLSYCDGEPNLVKATLCCEDRPGLNRDLTHAIRSVRAKAVRAEMMTVGGRTKSAVVIQWPAASGGGDSKEGVEVGALERALKAVIENRALVGSGMGRFVLGQKRAWNSYDSPDEVDCTFLLNRDASK.

>Araip.4C1AU.1 genotype-assembly-annot=K30076.a1.M1

MLVEEELQSLEMQRECKMEASQSSSPPVFNSERKNNNRAKKVEGQPSKNLMAERRRRKRLNDRLSMLRSIVPKISKMDRTSILGDTIDYMKELLDKINTLKQEIQVVDDSNGILSNDIHPNDILVRNSPKFDVERRNNNGDTRVEICCGGKPGMLLSTVNTLEALGLDIQHCVISCFNDFTMQASCSEELEQKRMVSSEDIKQALFRTAGYGGRCF

>Araip.Z8IAR.1 genotype-assembly-annot=K30076.a1.M1

MALEAVVFPQDPLSYTCNKDNYLYSIAASGGPWSNHEEPYGNNVTDSMDQWDSYNSNSSPEPCITTIDHDQASVPGANNSPLAPVEAATSSNTVAAGAASTGRRKRRRTKSVKNKEEIENQRMTHIAVERNRRKQMNEYLAVLRSLMPPSYVQRGDQASIIGGAINFVKELEQLVQCMNGQKKTKQEHHNNTVPFAEFFMFPQYSTHATRYCNNDNTVYPPCIEAGTKKPSWSSTAAVDIEVSLVDSHANMKILSKKQHGLLVKMVIGLQNLGFTILHLNVTTANDMVLTSVSVKV.

>Araip.NA6B3.1 genotype-assembly-annot=K30076.a1.M1

MAEFTADLHSFRPSFPFLDINTMELTNGFKGVMNVNSHILNNNSSLHMQDFLMPFNTSHSFISSPESEFSGLVHYVNHSDLPSSLTISSADNEIQQGKKRKAVDTPDTMSANSTPAVSESGSNTKNNYGRGKRGKKNETEEKKSKEVVHVRARRGQATDTHSLAERVRRGKINEKLKFLQNIVPGCYKTMGMAIMLEEIINYVQSLQHQVEFLSMKLNAASAFYDFNSQTDAFETVLRARASEAKELAKNEREGHGIQPTWPYCF.

>Araip.P4GTD.1 genotype-assembly-annot=K30076.a1.M1

MDQLGSRSEASPSSTNDKKIERRLIEKKRRKHMRMLYSKLNALLPIDHNSTSPKEVLPLPDQVDKAINYIKNLKKKVKMAKEKRESLLGMGRKRTRDAGSRRDRPKPPQLEVHEMGSYLQIVMTCGLDSQFIFSEIIRILNEENIDVKSVKSSLVENSMHHVIYAEISQSLLQLGATKVSERLKRFVNEPTSEIELQSDHQLLDFEIDTDELAELLDFLEEK.

>Araip.UVF95.1 genotype-assembly-annot=K30076.a1.M1

MDRGSQPSSSTTNKIERRLIEKNRRNHMKMLYSKLNSLLPNYNPKEALPLPDQVDEAINYIKSLEEKVKTAKEKKEDLLERMGRKRTRGSGGGGDGSGCSNTKPPQLEIHETGSCLEIVMTCGLDNQFIFYEIIRILNEENIDVKSANSSLLGDSNILIPQLLIQFGATKVSERLKRFVNGSASDIELLQPEFWDLEIDTDIWSF.

>Araip.R44YW.1 genotype-assembly-annot=K30076.a1.M1

MIMKPFLDIDPSMDLINQFIGLNTTTTTTTTHMNMCDNLFGSHHHHHHHQDDFQGNLEESFHQNHHHVNNNASSVVVPVISSSLLNYYPSENEEVHEGKKRKATTSGNSTPAASHHSESKIIKNNEVVHVRARRGQATDSHSIAERVRRGKINEKLRCLQNIVPGCYKTMGMAVMLDEIINYVQSLQHQVEFLSLKLTAASTFYDFNSETDALETMQASEAKELGKYGKEGHGGVSCYQPNWPLV.

>Araip.L83F8.1 genotype-assembly-annot=K30076.a1.M1

MADDQFRASANWWDSSRNVVRFESRESQSGPWHGVGDDEMKLPIRSSTMENSMSSSSSAGASSGRSSVVFHERLQQQQNLDSPNLSNDPNLHMMMGLGLSSHSSMDWNQASFMRAEKTSEMLQQETGMGLSNRGFSLDQTQFSPQYSSCSGDSNITSQQLLSSNFQMDSSSSAPLYCNTSMLQGLLGPESNNQAHQGPISNFPYSTCLNSNNNNNQLHFTNNAPFWNASEPAPVSIKDARSIFFPSLHQPFSAPSFDQQSKNISEVRDSGGSMLKKSENEQSSKRPRNETPPSPLPAFKVRKEKMGDRITALQQLVSPFGKTDTASVLSEATEYIKFLHEQVTVLSTPYMKSGAQTQHHQNSGKSKEADGPKQDLRSRGLCLVPISSTFPVTHEPTVDFWTPTFGGT

>Araip.GQB2P.1 genotype-assembly-annot=K30076.a1.M1

MMKKEEDQGQCSPQAIQNTIQSYQEQLFLQQQMQHHNPNGTDLYIGGGGSGRGLVFPPEASPIMQPPWSMSPVHPFSQAGPVHPVSHHNDNNNNHHDPFLVPPPPPSPYASFFNRRVPSLQFAYEGPSSEHVRIISDSLVGPMVHPGSVGPFGLQSELGKMTAQEIMEAKALAASKSHSEAERRRRERINNHLAKLRSLLPNTTKTDKASLLAEVIQHVKELKRQTSLIAETNPVPTESDELTVDTTDEDGRFVIKASLCCEDRSDLLPDLIKTLKELKLRTLKAEITTLGGRMKNVLFITADEEDYSSSNAGSGGGEQSLQLGHYCISSIQEALRAVMEKGGGDESASGNVKRQRTNINILEQRSL.

>Araip.7P91S.1 genotype-assembly-annot=K30076.a1.M1

MGEPCHNYWYSDMGIQDDDIFNQRYKINTSLIVDKDHIIREIMDHHDYHHQPAFSSESDNSHSPTNNNRSGGSGNTRGFVFNNNNNNGSMSMFSQQHQHAPLLLDMKASTSTSSPRSYILSFHDSTVIATTAAAAATPPPPPSLETYNNNGKRPYQHIEVPLENQAKKVRSSSETLDHIMTERKRRRELTERFIALSATIPGLKKIDKSTILSEAISHVKQLKQRVKELEEQRKKISVESVSFIIRKSHLINGTTNNKVDDEGAINNKAATSEIALLPTVEARVFKNDVLIRIHCMKQSGIMLKILGHLKSFDLSAISNSVLPFGNSTLDITIIAQVYIYIYAQY.

>Araip.0T7XC.1 genotype-assembly-annot=K30076.a1.M1

MISMEPYSIDPMFSGMSLQSLLNLNPSLLSFLTDHPPQPQLPNHLTLHPTLEPYFNFIPKRPRLTYSSCSSSSSSPPSSLRAKPLPPKSNFARQRRQKLSEKTRCLQKLMPWDKKMDQATLFEQAYSYVKFLQAQVSVLQSMPYHAPPSPSFNNGGGCVFGFGDLERLNRNQLLQVLVNSPVAQTVMSSKGLCVFSMEQLGLLTKLSERRLLLQNMNANMNMVSDSKPFFN.

>Araip.W4GJ9.1 genotype-assembly-annot=K30076.a1.M1

MVYSFYNSNHSSSPGFDVSRLISPSLHDYGASYGGLEPSHALILESEKIEQVNNGSEDARVGKNYENSDAKALAALKNHSEAERRRRERINGHLATLRGLVSSTDKMDKATILSKVVSQVKELQRNAMEASKGLLIPTDFDEVEVEPYEDHRSMSYKASICCDYRPEILFDLRHTLDALELQLVRAEISTLEGRMKNVFVYRCCKGKGDNNNTEACQALARNVQKALSLVIDKASNALEYSLRASLPCKRRRICLLEN.

>Araip.Z8QJX.1 genotype-assembly-annot=K30076.a1.M1

MSKNDEDATTKKRKGKTADHIIAERKRRQDLTRSIIQLSATIPGLKKMDKAHVIRESLSYIKVLQDRVKELENQIKDRRVDSAIFIGRSQDSSSTDKSTISYEITSDNNNGGGIYNELSLEIEAKVMEKEVLIRIQCEKQKNNIIMLKIHAFLDKLHLSIASNSVLPFGTSTLVIITIVAENKKDSLSLLQLMDNGGKFSMTMDELVKSLREDLMETNNNAW.

>Araip.I62ZT.1 genotype-assembly-annot=K30076.a1.M1

MAEDIRFFELNTGAKIPSVGLGTFQAENPGALAKAVTTAIKIYESLEVNHVKFIEFYDVRAAKASLGALNRICIPGKQIKLEHGHPRIAMYWLGAGAMQLNFATEKHFATEKQRREQLNGKYKILRSLIPSPTKALLQALGDRKGINRFGDFSAPLDEALIHVSLEQKVVTTFSAPNYCYRCGNMASILEVDDCKGHTFILVGARDIFLPGRGWRNHASVCLDRRDTRLDFYNWNRIKVKYCDGSLFTGDIEAVDPVSIVTLKILF.

>Araip.6VX9H.1 genotype-assembly-annot=K30076.a1.M1

RSEVFSNLINLFSSMFKLLLPHQIPKTLQELLPLEKIMSEKFCVSDEDKGVLESVLGAEAVAYFVSAVSNNFFSSVVASASALAGTDAGLRRRLCQVLEGSKWNYAVFWQVAGLKSGGSALIWGDGQCCDPKGGGAGEGGSEGDWSGVSKGDEEELRKKVLQKLDAYFACSVSKEANYARLDRVSDLHMFYLASMYYIFGFDSPCGPGGSFKSGKSIWVSDSASCSNQLESRSFLGRSAGLQTVVFVPLKAGVVELGSVETVPEDQGVLDLVRTAFGESSSGQAKAFPKIFGRELSVGGGDAKSQSITISFSPKLEDDSGFTSDSYEVQALAANHGYGNPSNGSMGENSEAKLFPQMIGGNYNAQTRVSSLDLGNEDSSSTHLDERKPRKRGRKPANGREEPLNHVEAERQRREKLNQRFYALRAVVPNISKMDKASLLGDAITYITDLQMKIKVLETEKNMNNNNEPMFPFSDIDFQAREDDTAVVRVSCPLDLHPVSRVIKTFQEHQIMAPESGVSTSEGKMIHTFSIRAQGSEAAAIQLKEKLEASLSKN.

>Araip.HWR4Z.1 genotype-assembly-annot=K30076.a1.M1

MVMKILGKRAFDYLASNSFITNESMLMATGSFENLQNKLSDLVERPNLNHFSWNYAIFWQFSQSKFKKDCVVLGWGDGCCREPIEGEEEREALRLGFDDDEVVQRMRKRVLQKLHTIFNGSEEENENYAFGLDRVTDTEMFFLASMYFSFPKGYGGPGKCFELAGIKTVVLVPTELGVVELGSVRIVDENLDLLKAVYSVFSSSFAHSSSFNDVGVPVPKIFGKELNVGNLNSGGKNFREKVVVRKIEEKKSWNGYPNGNNGIRFPNNARNGVNGSSWAVNQGLSQGCLGGVFPPIPRSCLSNMAKQADCGGKKDSLLEKFQRPPQGQVPMQIDFGVGSQSFGRSIVGECEISNSDSLHKEEKPSATQEKRPKKRGRKPANGREEPLNHVEAERQRREKLNQRFYALRAVVPNISKMDKASLLGDAIAHINELQAKLKALEYEKSTLKSTSKANLSGIEANLRDRTCSFKVDIEADQDGVIVKVSCPIELHPTSKLIQALKDSEMSILESTFNATNDHVFHTFVIKSHASEQLTKEKVIAAISGESNSRKLIFA.

>Araip.FH6AP.1 genotype-assembly-annot=K30076.a1.M1

MQPCSREMQSLNSLFNNQQQIQMDPATSQEDDFIKQMLSTIPPPPPPSWNLDHTPPNPKPYWLDNDNVAFPNYDDPSTLASKFRNHQITANSSSKAAAAALLLQQQLLMTRGLSGADSAILHHMFDSSQNDVVDASSSSFKSPNPTSADGSVQALYDGFTGSLQGAALASNQTHHFQHPQVQTSSNPNQMQGQGQGTSQGGNPNPNPNPGQPKQRVRARRGQATDPHSIAERLRRERIAERMKALQELVPNANKTDKASMLDEIIDYVKFLQLQVKVLSMSRLGGAAAVAPLVADINSEGGGDCVQTNGNQTAGASSSNDSLTMTEQQVAKLMEEDMGSAMQYLQGKGLCLMPISLATAISTATATCHPRNPIMNPTDGPSSPGMSVLTVQSANGDAVKDATSVSKP.

>Araip.JE9KX.1 genotype-assembly-annot=K30076.a1.M1

MPCKSQTVVCIPLLDGVVEFGTIDKHSTSNPASSSDCLIYTVADPPLHLTHQEDIKFIQHVKSFFIEHHHASVHPPPPPPKPALSEHSTSNPASSSDCLIYTVADPPLPPPNLTHEDNMEEDQDNEEEEEEEEDEDDEEEEPILDSEDEKRRNNNNVSGGITEPSELMQLEMSEDIRVGSPNDGSNNLDSNFHLLAVTEADNHSGQVDSYRVDPTQRWDPIQSPLDQLQVQLPVFSPIEDQILTQEDDHYSQTVSSILQNQSTRWAESPSIACYVTCSNQSAFAKWTSLVADEHLHAATADGSSQWLLKYILFTVPYLHSNKNNEENNSSPNNTNPTSSAGPTDRLRGGGKGSGTPQDELSANHVLAERRRREKLNERFIILRSLVPFVTKMDKASILGDTIEYVKQLRRKIQDLEARIRQMEAEQQRSRTPSTTIEVHHCGSSNKEQQQHTVVLGHEKRKVRIVEGTKTATKVAAAAATEASVQVSIIESDALLELECSHREGLLLDVMLVLREMKIEVIGVQSSLNNGVFVAELRAKVKDNNCGKKVSIVEVKRALNQVIPHTGD.

>Araip.78HJ7.1 genotype-assembly-annot=K30076.a1.M1

MDSNSLQGYQLEQQQSSSSGLLRYRSAPSSLLANLTPTFTESQALLSRLANSNSSSSKNNISKNDTVSHSFQEFPDTNTNTNTKTNNKGCDSKGLRRMNSGGRGYGGGGGLPPHYPRNTSSSSSSSCSSSMDGSLGLVMGSMGMDQKGFGVSSSLNLLRQNSSPADLFSNVSFQNGFATMKGVGNYGHVNSTSEELSPSMNRLKNQISFSSRTASSLGMLSQISEIGNEEIGANSPEDGRQGGSNGDALHYSPGFCYGSWSDAPQHSENLTGLKRGRSSNGKLFPDVQNGELGNQVHMLSHHLSLPKTSSEMIGMENLLQFPDSVPCKIRAKRGCATHPRSIAERVRRTRISERMRKLQELVPNMDKQTNTADMLDLAVDYIKDLQKQFKNLSEKRANCKCISMRRADTNQIV.

>Araip.9H3WY.1 genotype-assembly-annot=K30076.a1.M1

MPFLQMLQSVEYSPNYSSQQQCFFPFKDPTNFQTLLRLQHLKNHNNNNNNNNNVQELDQKSCVTHDVMVEMQQQQQQQQSHSPVKSESYELHQQHHQASASASCCVENNNNNTTPTKAITAATEKCGGRGNSNTQEVCQKSQQVGSVATTTRERKKRKRTRPAKNKEDVENQRMTHIAVERNRRRQMNDHLSGDQASIIGGAIDFVKELEQLLQSLEAQKRMRRKNNNNNSSNKNEDAIGFGSSSNSSSTTNGGYDGIMRSSTTSLSLLSTEEGNFGGGGGNGDELKAENKSESAEIEVTLIQTHVNLKIQCKRRHGQLVKAIVALEDLRLSILHLNITSSSDDSVLYSLNLKIEEGCRLRSANEIAEAVHHIFNFING.

>Araip.PVV4Q.1 genotype-assembly-annot=K30076.a1.M1

MMEIASSNYLAEFGMEEYSSSFQEYPMMMNSFEEMLDKFEMDMQSMSSASPECYSNSETKPPPHQLQTPLNTITTTTTTAAMPSRSASPSPPKLISFEAPSLPNSSNIKNPNLMMDDHIPFSAFFNYDNPPHKVLPATARNPVQAQEHVIAERKRREKLSQRFVALSAMVPGLKKMDKASILGDAIKYVKQLQERVQFLEEEKARKKSMVESGVAVKRCFVFVDDEDNNENEISAAAALLDGNCNTLPEIKARVSGKDVLIRIHCHKQECKNSSRGAREAAILSVLEKHNLTVHTTTSLPFGNDTLDITILAQMNKEYSIRTKDLVGSLRVALTQFS.

>Araip.0B5Q5.1 genotype-assembly-annot=K30076.a1.M1

MGSSENPNWVLDYAYLDDIHSLSDPSNFSWPPPPTSLSADLHQSLPNSHGPDQSASRKRLRSSGSKACREKMRRDRLNDRFLELGSILDPGRPPKTDKAAILSDAVQLVSQLRGEAQKLKESTENLQEKINELKAEKNELRDEKQRLKAEKDSLEKQLKALNTPPGFLHHPPTLPAVFPHAPGQVLGSKLVPFMGYPGVSMWQFLPPAAVDTSQDHVLRPPVA.

>Araip.13D8C.1 genotype-assembly-annot=K30076.a1.M1

MEELNASATNWLSDLDIDDYELFSECNLKKFLDEDEENQFDEMVMSAVLGEEEEEEEERPTKQLRTCSSSSITNYVSSNSSSSSSPTSQILSFENSYNPSSQFYGFEFDATPNDKVSPQPQVGNNNDKNKVEQTRKPQSQGSKRHAAHSHDHIMAERKRREKLSQSLIALAALIPGLKKMDKASVLGDAIKYVKELQGRLKVLEEENKREVESVVIVKNPRFITSSSDDDSSSCDDDTLEADGEAAVAHVEARVAAAEKEVLLRIHCKKQKGIYVKLLSEIQSLHLYVVHSSVLPFGDSVLDITIVAQMGTEYKLSIRDLVRNLRVATLKTMSSS.

>Araip.V8XWP.1 genotype-assembly-annot=K30076.a1.M1

MYRLASKLASSITSSTSWSVIHCVVISSRNYTSKDINFGVGARATILQSVTKVANAVKVTMGPKKSYRNPRITNDGVTVAKSIKFQDKAKNIGADLVKQVAKATNTAVGDGTTCATVLSQAILTEGCKSIAAGVNVMDLRNGINKAVDAVITDLKSRAVMISTPEEITQIILEIMFVEQLELLLWLKKKIKMHSNNGRKVKIGVGVGVSVGVLCGLLLAFCLIWKRRKMATLKETNAALKDQSKKEQEEDPEVPLYDLSVIASSTNNFSHKNKLGEGTLENGQRIAVKRLSASSGQEIKEFKNEIALIAKLQHRNLLNFATEKQRREQLNGKYKILRSLFPSPTKMDRAFVVGDAIEYSNGIYRIGLTHYSEGSTCLIFIPFIKWFKILINTIIDNLEQRFYKNLRNENLASENVVLCIYK

>Araip.L8LRC.1 genotype-assembly-annot=K30076.a1.M1

MTVLERQQARTKKCIQQDLQDNGSSLFDVFSDDYSSLIHMPPAPSSSSAISSFVSPKKRKSESHKVVVAQQSHTKKEKRVKVSAEEDSSKMSEQITNKDEVASAENSIKGSTSEPHNNKPDYIHVRARRGQATDSHSLAERVRREKISERMKYLQDLVPGCNKITGKAGMLDEIINYVQSLQRQFLSMKLAAVNPRLELNVDDLFAKEVFPACAPNFEGIGMGSSEMTNNPAAYLDFNSVQQLVSCSNGLINNIGMNMTPSPDMGLRKTIAAPLSVSLPETFLDSSSCFNQVLPPSIWEGEFQIQNLYNVAFDQPRTSSFPPSHPFTGTYEL.

>Araip.HA94C.1 genotype-assembly-annot=K30076.a1.M1

MGNLYDNRLTVSVDELYFNAENSSINKRGRMGRRNYDADDTVVYKSKNLETERRRRQKLSDRLLMLRSLMNKATIIEDAITYIKNLKDKVDSLTRELQEIEATSETTLVEPKTNETNNGEAMKEWGIKEEVRVTNIDGKKLWLKLIIEKKKGRFTQLMDAIHSFGIELIDTNVTTMKGALLITASIQGMDGEALVVQQTKELMLDIINSTHTY.

>Araip.6K1VA.1 genotype-assembly-annot=K30076.a1.M1

MLHCLNSNASGSEMTVLERQRATIKWQHNNNHHQGYSSSSSPSPSSSSSISLVQVQSSSPACLAAGAWPKLDNNFLMGFTSSPSSGFPLPPPPPPTTSAASFDLNSAISRTSSCPLPSLAEGKETTTPNNKRKTDKFHNTKVVASDNDNKDKRVKVAAPNDSATVGKKGTQDCGDNSNKDNSKVSEVQNQKPDYIHVRARRGQATDSHSLAERVRREKISERMKFLQDLVPGCNKITGKAGMLDEIINYVQSLQRQVEFLSMKLAAVNPRLDFNMDDLFTREVFPACAASFPAIEMSPNMANPASYLHFNSSQQIDAGINPQDISLRRTISAPVSAPETFLDSSCFTQTLPSSTWEGDFQNLYSVAFDQARAASFPSQLFTGLLEPSNLKMEMEM.

>Araip.L2MXT.1 genotype-assembly-annot=K30076.a1.M1

MGGHENAMGFHHGNESILTNNVNVSEMDMSSSMSLAKSSSSDVVVPNRSSMVTTHNSDFGNSSSYPFVQYMSDSTNLEGIMVHKIPSFGSGNFSEIVGSFCQEGSSDIPNMGFRPSYNHGNDAGTERAPMNEQSQVEDSITEDGAPGTAPSGNRRKRMLDHDSSFSPNKNAGGDELKDSPRTISDGAKEHEKKAKVEQNVSADLRGKQPAKQSKENSPSGEAPKENFIHVRARRGQATNSHSLAERVRREKISERMRLLQELVPGCNKITGKAVMLDEIINYVQSLQQQVEFLSMKLATVNPELNFDVERILSKEILQSRLGHGIVGYGVGMSSPHPFSNGSFQGNMAGMPSTSTQFPPLPQGVLDHEFQSLYGMGYDPNTALDNLGPNVSCLSGRLKTEL.

>Araip.E7Y1X.1 genotype-assembly-annot=K30076.a1.M1

MSSDVENAMRLQQEEKKGILLGLGTSGSEMASSNSEPSNVANPFLASSAWDPLTPLTQLQTTNTTTLVGDPSTSIPIPMVSHHNNEFSNSLLYTLVLENQQAPHIVQYMSRDMVPNPKVQVPISYGSGSFSEMVGSFLQSPNSEHSQGEGEGEDSGSAPSGNRRRKRGHEHNKNAEDCSGKSSDGRKDQDDQKKAKVEENKDKSAQSGEAPKENFIHVRARRGQATNSHSLAERVRREKISERMRLLQELVPGCNKITGKAVMLDEIINYVQSLQQQVEFLSMKLATVNPELNLDLERILSKDILQSRIGGYSGGISCSQAFPSSSFQGTLVPMPTTSNQLPPLPQSVLDHEFQNLYGMSYDSSTALGNMGHNGGSKSEL.

>Araip.L8YHH.1 genotype-assembly-annot=K30076.a1.M1

MADNKRKTTSNSSSFNNNKELSAGREGRSSKKQRKSNNGGSGVKKGEEKEKEEAPTATATGYIHVRARRGQATDSHSLAERVRREKISERMKMLQRLVPGCDKVTGKALVLDEIINYVQSLQNQVEFLSMKLASVSPFFFDFAMDLDNDNNNGHLVRPLDQYTGGQFWEVEEDQRQKQLLHPYGLGSNLVGQHNE

>Araip.X784U.1 genotype-assembly-annot=K30076.a1.M1

MEIHQQNNVANCSPENNNHQNQDSFYNNNNTDHNNLQFDSSALSSMVSSPSTAAPNFNMIQNDNSFVIRELIGKLGTIGGSSAEISPHSQTVALYNNNNTTPSSSCYSTPLNSPPKLNMPKPPPPSSSMALNSATVAEFSADPGFAERAAKFSCFGSRSFNGRTTTTTTTTTTSPLVMINNHNNNNGAELTHQRSNPIHRVSSSPSLKSLGSQMEKICSSPLEMAANSSQEESTISEQNPNVEGAALKIASSSDMSSRKRKGSSRAKAKDSTKPPEPPKDYIHVRARRGQATDSHSLAERVRREKISERMKLLQDLVPGCNKVTGKALMLDEIINYVQSLQRQFLSMKLASVNTRMDLSIENIVPKDYPLTFCEDDLHTIVQMGFGQTSNRTTPIHSPSFDGSNQVSQMKVEL

>Araip.PF3JC.1 genotype-assembly-annot=K30076.a1.M1

MPTMASFLDPHQASITATTDFGGFHHHNLEELAQPTLFQKRAALRKNNSSSQQQQQGGVLGEGDNNNNSMKRKVMMEMEDNGSFDGSSAFNYDSGDDLIETNNKKNNQKGGGAKNKKGMPAKNLMAERRRRKKLNDRLYMLRSVVPKISKMDRASILGDAIEYLRELLQRINELHNELESSPSTTTTTTNSGSSSLITAVPTVVASSSFYPLTPTPPSLPTRIKEEICPTSLPSPNAQPPRVEVRLREGRAVNIHMFCGRKAGLLLSTMRALDTLGLDIQQAVISCFNGFALDIFRAEQCKEGQDVHPEQIKAVLLDTAGYPAII.

>Araip.0HI1A.1 genotype-assembly-annot=K30076.a1.M1

MDPPPTILGLQISLCNHIQEDNNNNINIGVPLIQDHHHHLWTTTTTTTSSGDDNTAQPSCLSLNTTNHITINHNMLNSHHTPPPPSSTVEFSTHDDDKLLLKTFFSAQDFYSNTDTTTTTTFCGVPSSSRRNFSHIYPTINISNNINNHSSPSPSSSSNNMTSHSFDLPAFMTTTTHGGGAAAHQQVDLGLATFSDNHLSFHLDHHHSQHHRPTHASSPPCSNSTTSTTANNNSHPSQYFSNGTVDTKRPCTSIMDTKASQSLTASKKSRSESRPSCPPFKVRKEKLGDRIAALQQLVAPFGKTDTASVLMEAIGYIKFLQSQVETLSVPYMKPTQNQTNSRMMQGVSTIGGGNGEPKQDLRSRGLCLVPLSCMSYIAGDASNDAWQQSNFGGAT.

>Araip.8JJ8B.1 genotype-assembly-annot=K30076.a1.M1

MDDNNLSDIFQDKDFGGDDLFAILESLDDFTPTNTVQFPTLLESSSLPSPPTKRRKLLVPPPPPSPNAAEDGQQRMSHITVERNRRKQMNEHLSVLRSLMPCFYVKRGDQASIIGGVVDYINELQQVLQALEAKKQRKVYTDHVLSPRLVSSPRTSLSLPLSPRKPPLSPRLSLPISPRTPQPGSPYRPPAPCRLLQPAAAAASSYISPAASISNSLEPSPTTSSASSINDDINELVANSKSAMAEVEVKFSGPHLLLKTVSQRIPGQALKIISALEDLALEILQVNISTADETMLNSFTIKIGIECQLSADELAQQIQQTFC.

>Araip.81R1Z.1 genotype-assembly-annot=K30076.a1.M1

MAEDQFQASGNWWENPAIRNNWQPHQQQQDESDHMMKPTRVSMDSSGGGAAGGGSSSVVFHDPQKLQPPDSAAAATSSTDPNLHFMALGLSSPPIDWNQQPSFLSMLQEEGGGGGGMGLSAQQVHQWRSAESEFKNNRGFCLEQNQFSPQYSSGDSTVTSQQGFHHQHMDHSAVLYGSPSSILQGLLGPDHHQQQANHNFSYPTSNYGLSSSSSNDFLSNSSSSPITTTNTSLHFTNNAPFWNAAASDPKDVRSTTFFPNSLQPPFFTNPSFDVQSKNMSEMRDSGAVVKKSGSEPTPKRPRNETTPSPLPAFKVRKEKMGDRITALQQLVSPFGKTDTASVLSEAIEYIKFLHEQVLSTPYMKSGAPIQIQQGSGKSKEGEGPKQDLRSRGLCLVPVSSTFPMTHETTVDFWTPTFGGGSR.

>Araip.B6R33.1 genotype-assembly-annot=K30076.a1.M1

MALEAVVFPQDPLFSYASSTTTTKDYFYSSFLGSHHHDLIGIINNNIIQQNQATNNSDSSSPPSNNDHWDHSHYATSSSSPDCCAATVDQPSVPTTTGRRKRRRTKTTKNKEDIENQRMTHIAVERNRRKQMNDYLAVLRSLMPPSYVQRGDQASIIGGAINFVKELEQLLQSMEGQKKPNHHDTTAAGGAAGMNSSGQSSPFGEFFAFPQYTTRGHHQGNNNATTMAAEQQQKQWAPAVADIEVTMVDSHANLKILSKKRHGQLMKMVVGLQSLSLTILHLNVTTLHDFVIALTFVANFEDGCQLNTVDEIAAAVNQLLCAVQQEASSFNEIN.

>Araip.8T85I.1 genotype-assembly-annot=K30076.a1.M1

MALSTYLNGDALQNSLMSDNFTTNFHELTAPEELTLDHYYHQHNQEGSFPNNFFFDPYFDLNNEFFHTEILSSHQLLGPDCTSSHNSFISPNDLFQTEYSNVNTLLSCPKRQKYFHEDEELQLSPPLKEYASPSLFDGFILNSSSLLPSEEAALAEELLLPAAVAAPSSDFMVPEVVRNGFCVGINNESEKKDNERTISAQSIAARERRRRITEKTQELGKLVPGGPKMNTAEMLHAASRYVKYLQTQVGMLQLMNTLQKENEVAPPSEDLCALVTSPFVQEKLYSEELCFVPKDFVTTLTNQHDVRSKPTILKDLKELIETNNLQKKA.

>Araip.74SLF.1 genotype-assembly-annot=K30076.a1.M1

MDSNNTNSGLLRTELEKLISSNNKNKRVVNNSSEHLKREEGNNNCNYSFGSQNHVIYQNNQHYHQIHQGLAMASSCNNGFDGTLFGATNSMDSENNNDNGTQNKMGSNLVRQKSSPAGFFSNYSIDNNGTTTSSRSSSGLQCTLNFSSRSSSCSKSRMPKIVESGNEGMESNCCYMPRFTGDFWDASEFNSSKRGTNNGEIMFSTSNAMEAQVGLTHHLSLPSSSTKIGGMEKLFQIQGSAPCKIRAKRGFATHPRSIAERQTSTADMLDLAVDYIKGLQKQVKILTDTRAKCNCSSNNQKEQ.

>Araip.778BR.1 genotype-assembly-annot=K30076.a1.M1

MEIYYDDHHDFLEELMALRRETLMETNYDNTTFHSSFPPNSSSSSSYQQDLILPHQIYDDDNFFNQIYDSLLFDEQSPPPLMMDHYSSSSYYSNNTSSNLDSYYPPLSFMEEEELQNLEIETATRCKVEPVVTVTCCDSESPAAIFNADSVMGNKEIRKNSRSTKKVVGQPSKNLMAERRRRKRLNDRLSMLRAIVPKISKMDRTSILGDTIDYMKELLEKINNLQQEAVKVEDSNMAGGIFKDVIKPNEILIRNSPKFAVERGVDNTRVEICCAGKPGMLLSTVNTLEALGLDIQQ.

>Araip.5HP4H.1 genotype-assembly-annot=K30076.a1.M1

MLRVVAVVVEESGNHSGGGNGNLSLSGGVNGGDQKGKKKGMPAKNLMAERRRRKKLNDRLYMLRSVVPKISKMDRASILGDAIDYLRELLQRINDLHNELESTPPGSSLQSSTTSLQPLTPTPQTLPCRVKEELYPGVLPSPKNQPAKVEVRVREGRAVNIHMFCTRRPGLLLSTMRALDSFMILSPNFVWAFQMLIESEWAGMKLKRNGLVVATLRH.

>Araip.K0K3F.1 genotype-assembly-annot=K30076.a1.M1

MEKSNTPMDTSAASWLSELEIDEDYNFFPDLDFDLVDEEDFLSHELIASEDLQGKSALQDQSLSAECNSKELSNCCTDEMMSFEEMLRNINDETFSPKLSSSSSQILSFDNSDSLSSSPPNNTTQFYELKNSLNQRQDSVETPNNNNNKKSDSLNTQNVEAKSTSTLGKRSPSHAHDHIIAERKRREKISQSFIALAALVPGLKKMDKASVLGDSIKYVKELKERLAVLEEESKKTKALPTVVLNKAEQLHAHGAAVFSSLCEEETIDGLPQVEAREWGQQVLLRIHCWKEEGILVRILSEIQSLQLMVLNSSVLSFGDSILDITIIVQAGEGYNLTLNELVKNLRMATLKLMS

>Araip.007DK.1 genotype-assembly-annot=K30076.a1.M1

DNEAGNNGGGVVQEVGERLSLPKLMMMMMMSKSNSSKSIVNGGNIAKKARRSSSECLDHIMAERKRRQEITQKFIALSATIPHLKKIDKASILSEAIAYIKQLKEQGKKLEEESRKKNRTVIESVSLVNKRHGAYELVEARALEKQVLIRIHCHGHKGVPQVFNHLTNLDLSVVSTSVLPFGTCALDITIVAQMGEKYSASMKDLVQSLRLAIPLC.

>Araip.1E1W9.1 genotype-assembly-annot=K30076.a1.M1

MEENSLAETCWFNILANDLLRFVYFKGNWQLDEAFSGDYYNFTSPDSGALSAVSKDIVFDKNSMKQINERLFALRIVIPNITKMDKASIIKDGTEHLHEKERRIQDEIMEVKSKRLNNPIITASAAAGDYDFEQDLPIVIRSKKKRPDNLFFDTSTSSSSINNTNYFPIELIDLYAQLGSSWQECKNEN

>Araip.42MZK.1 genotype-assembly-annot=K30076.a1.M1

MKGSRDAHEPSHMLSFLPLRLSISDLQLLCLVSGFVNADAKAVDKASVIRSKHSVTEQRRRSKINERYVQRASVISSVLSASVFDVGALNHDASGCQAVNTVGIVICYEDIGDLFFRFQILRDLIPHSDQKRDTASFLLEVIEYVQYLQEKVQKYEGSYQGWGHEPSKLMPWRNSHWRVQSFVGQPQAIKNGSGPVSPFPGKFDESNISISPTMLSGNQNMIDTDQGRDIVSKASEGQTDLASKGIALPLGMHPNMSVPVRSDGVLSHPLQGSVSEAQSTECPAASEPLSQQDELTIEGGTISISSVYSQGAVKNSNSYEYELLNNLTQALQSAGLDLSQASISVQINLGKRANNGPSCGTSSPKNHDMVPSSNQAFAQFRDAGSEDSDQAQKRLKTFK.

>Araip.37BUF.1 genotype-assembly-annot=K30076.a1.M1

MVPEKVKKQLALAVRSIQWSYAIFWSSSPTQPGVLSWGEGYYNGDIKTRKTSQVAEHNSDQIISLQRSEQLRELYSSLADSKSCSQTKRPSAALSPEDLTDTEWFYLVCMSFVFNIGQGLPGRSLAYGQPIWLCNAHSADNGVFCRSLLAKSASIQTVVCFPFLDGVIELGTTDPVSEDYSLIQVIRNSFLDILETNLPNNPGANLNTRNKEEGGVACGEFDHNAYGLKLTPEVIGYELINITSPTTSSNALQANQQTDGRTMFPTEFSNCFHNSRNSSDCLSENHCAQDLQQCNDNPKTTLVNLGSDDKHYQRILSALPIRPDDDRLIMRVHLRNFRGESSFAIWKQLGSMDCQRSRRGGTPQNLLKKVLFEVPLMHMDGLLESQEKIGSKDRMRLLEVDDVGMNHALSERKRRAKLNERFLTLRSMVPTISKDDKVSILDDAMEYLRKLEEKVRKLEAEKDVTDLDGIVSTRTSQDMVERASDNNSNKNSKSVSNKRRACEAAGDEEMNNSEIDVGIYVSESEVVIEMKCPWREGLLLETVEALSSLHLDCHSLQSSKAHGTLYLTIKSKFRGANNVAPVKRIRRTLQKAAMKS.

>Araip.LV58R.1 genotype-assembly-annot=K30076.a1.M1

MDAHQETLIFNNDYEVHDFTEDPNFDQLINLIRGDNNNEDDAAVFFNYGSDIIINDCFLDDNQNQNDLLLPCPTTAHNPYDQIHQSHNNNSSNEVMMNNVCDTNLISSFSCFDEGVVNRENEGEYSSATTTATDEANLSGKPRVKADRSKTLISERRRRSRMKEKLYALRSLVPNITKMDKASIIGDALSYVHELQAQAKKLKAEVAGLEASLFVSENYKASFDDNYIKTVQVTHNSHPINKKIVQIEMVQVEERGYYVKIVCNKGGGVAASLYRALESLVGFSVRNSNFETVCDTILLTFTLNVKGFEPEVNLPNLKLWVTGALLNQGFEFMGNFHY.

>Araip.SB6JF.1 genotype-assembly-annot=K30076.a1.M1

MDESVFFHNQYPMMDSSTITSSPTPWPQINLDDIDFNQIQESFSSASSPKSYTSNKRFNSSFSPIERPTKQQKNAYSHGISTNTTTTTSNEFMVPKASSSSSSQIISFEQHSNASSVASYNHHHQQLYNNNPSSDQGNHVLKPKTESVCSENLDFASVVSQADKANKGSAGAATTRNPTQAQDHVIAERKRREKLSQRFIALSAIVPGLKKMDKASVLGDAIKYLKQLQEKVKILEEQVAEKTVESAVFVKRSILFAEDNGSSSEENPEIEARISGKDVLIRIHCDKHSGIVPKIINEIEKHDLSVQSSSFLPFGNNSLDITIVAQVNINFNFSLINLL.

>Araip.K2DDF.1 genotype-assembly-annot=K30076.a1.M1

MARSAKGHQEELDDDDEEHFTADTSSPHKVKLDESNSGKRVNPHRSKHSETEQRRRSKINERQALFQVLRDLIPQNDQKRDKASFLLEVIEYIQFLQEKLQIYEQTCEGWNQEPTKLIPWRGHHGPAENKADTSQAMQNGSAHEKNDVSPLLPKNVQNPIESDLSTTTIQKGHTIGSAVEAVPMGMQMRLDAFDPVVSSSMPNQRLHQPISNADMSFQIQPQVLFSKPSSGNYMVSDNVLMEHEELTNESESQSISNAYSQGVLDTLTQALQSSGVDLSQTSVSVQIDVGRRSNPGFIPSASSSKVYGNQFVTNRVNYCSEDSDQSLKRLRRDAT.

>Araip.P7G4I.1 genotype-assembly-annot=K30076.a1.M1

MMEGVFFLPEAARTGYLRSIVQSVGCAYICLWSFDPTSSPNNRLFFLDGFYNNVRNNQQASSSLGSVAQQLFNQFRTLRFDANDDRIPGLAFRNNRPYLEVQQPELLRLAWTQIQKQFFQEARIKTAVFMGCNKGEIELGFLNMSQAEIQTALSSLFQEDFSSGRQIMDHQNNPPSSSSSSLRSLSTTAGSPEYSSLLFNIPPAGTLGTGGVTTGGATAGAIVPNTMSPLSSNTQSALLTNYVFPSHHQEIENETLMRVFLNAISPQQHQNLPYNITVVHPEGSAFKRYRTEPEPGPERGPESLRSRQSLMKRSLAFFRSINAMRIRERIQATRPSSTQLHHMIAERRRREKLNDNFQALRALLPPGTKKDKASILTTAKETLSSLMAEIEKLRIRNHELESRLPESSKESSATDQEISKTMLLVPPNERFHVQISDVPQSSSSSSSEERRVDLHVALRGQISQIDAVIRLLEFLKLAQNVSLVTMTTNTNNVGQGNNNYINQLTFRLRILEVCYLSFIKMHVLIGSEWDVSAFQEAVKRVVGDLAQFQVDH.

>Araip.07856.1 genotype-assembly-annot=K30076.a1.M1

MQLFLSTTNIVVDSFDEEVTIIGQNQQMENNIGNYLNTLLVEEYDANSNNNHSTYTTISTSTHHDQNSFDHYYSNMNNNNNTNNIQFPCFPTQTTDLLNLLHFPTSPKNSSISFENHNNNIITCLPNPSSSSSNIIVGQESSNFSNVFNDPLLHLNLQAPPLLHQSSSSMRELFHHHGYYNMVPTTSRSDFVFGLGENNNNNNNNNDIIVELENGGYGRDVLENGVLEEFTNHHQEVANKKRGGKRSNNNNIKQFSSTNTERQRRVDLGGKFDALKELIPSSTKNDRASVVGDAIDYIKELLRTVNELKSLVEKKRYEKQRVKKKLKIEDEEEEEKEDDENNSSSYSESLTRSSWIQRKSKESEVDVRIIDNEVTIKIVQRKRVNDNNCILVYASKVLDELKLDLQHVGGGHIGDFCSFLFNSKICEGSSVYASAIANKLIEVMDRSLLTI.

>Araip.X1DZZ.1 genotype-assembly-annot=K30076.a1.M1

MLTENFKEQLALSVRSIQWSYAIFWSTSSTQPGVLSWGEGYYNGDIKTRKTSQGVELNSDQIGLQRSEQLRELYKSLKTVEASPQTKRPSAALSPEDLTDTEWYYLVCMSFVFNIGQGLPGKALAKGEPIWLCDAPSSDCRDFSRCLLAKTVVCFPFMDGVIELGTTDHVSEDLSLIQQIRTSFLDILDVGVHNVHGAFNVKQSQEVGGALISITSPNNSSNAFQANQPPDETLMVERINNGTSQVQSWQIMDDELSNAVHNSSDCVSQTLHSPENVASLPKGENLTDSAKDLQKCNNSKMTLVDPRSDDWHYQMVLSTLLKSSDQLIMGMHFQNFHQESSFSSWKKAGSVSYQRPRTGGSSQALLKKVLFEVPRMHLDGILEYQEENDFKEAVRTEADENGMNHVLSERRRRAKLNERFLTLRSMVPSITKDDKVSILDDAIEYLRKLEKRIKELEAHKDLTDREARTKRSPQDMVERTSDNYFNKTDNGTKSMAKKRKSRDIDDTRPEINSESLLKGSSTNDVAVNINEXLWKQLAVSTWNHLFGFNCTSSPMLTEPTIVSAKRIKQTLQNVAFKC.

>Araip.UL9LC.1 genotype-assembly-annot=K30076.a1.M1

MVEDSIDNNNNMMKMESESSSKNKNIIGENNNRNSNNNLNLQKGNNNKKKTGMPAKNLMAERRRRKKLNDRLYMLRSVVPNISKMDRASILGDAIEYMKELLQKINDLHNELQSMPPTAPASSLHHPLTPTNNNTATLPPAAASLPSRMMMKETASCPTSSLPTPNDQPARLEKTVFTSFDLIVDLKFLLIQDQEMILNSDNCVHYVKVEVGLREGRGVNIHMFCDQKPGLLLSTLTTLDNLGLDIQQAVISFFNGFAMDIFRAEQCNEGQDLHPDQIKAALIEASEASGFHNII

>Araip.8I39N.1 genotype-assembly-annot=K30076.a1.M1

MENIGDEYHRYWETSMFLQTQELDNWGLDEALSGYYDSSSPDGAASSAASKNIVSERNRRKKLNERLFALRSVVPNISKMDKASIIKDAIEYIQKLQEQERIIQSEIVELESGMMVEKKNPNSYEFEHQELPMLLRSKKKRTDELYDSLNSRNSPIHILELRVTYMGEKTIVISLTCNKRTDTMVKLCEIFESLKLKIITANITSLSSARLLKTADEEEKDLLQIKIQTAIAALNDPLSPMSI.

>Araip.6Q4X9.1 genotype-assembly-annot=K30076.a1.M1

MASRESTNWLFDYALIDDIPVHDATFAAPSSGFNWPSNAINGSSNVGVEIDGSLGDSDGIKESGSKKRGRSESCAPSSSKACREKLRRDRLNDKFVELGSILEPGRPPKTDKAAILIDAVRMVTQLRGEAQKLKDSNMGLQEKIKELKAEKNELRDEKQRLKAEKEKLEQQLKSMSAQPSFMAPPTAIPAAFAPQGQVPGNKLVPIISYPGMAMWQFMPPAAVDTSQDHVLRPPVA.

>Araip.EV6EL.1 genotype-assembly-annot=K30076.a1.M1

MLIVAVAALTIALLRRSSHHRLLYPSSRGIYLREATAFQQSTELGLNLDQGKGADRVTAPEVGFLNPPEEASGSGKHFHDDVLDARAKNVFHGQPMPTTIPAAPHPPTMRPRVRARRGQATDPHNIAERLRKERIAERIRALQELVPSVNKVCNCSNAEQAFSFFDSACSALLPCAIRFCPNFHLLLGCLLAKMEAEKRSLWSWTASIEKRSLFPNAKSVALGAEKRSL.

>Araip.865PM.1 genotype-assembly-annot=K30076.a1.M1

MAERKRRQQLTQMFIALSATIPGLKKTDKASILGEAINYVKQLQERVRELEKRNNDNKRGPTEPVIFLNKTQLLCRNNEDSTSEEEEEEEEEEVEDWRSKEEKQVLPDVEARMLEKEKEVLIEIHCEKENGIEVKILEQLENLHLSVTGSSVLPFGNSTLGITIIAKMGDAYTMTLHDLLTNLRQLLLNIDQQHY.

>Araip.UT46I.1 genotype-assembly-annot=K30076.a1.M1

STTILPTSLPIPCPPETTHLQYYLGYFTFYYCRQKPMSCGDRPNMHEQPGCFDPNTMAEGVSTPKLKDTFPQTLSDPSSSPSPLIVVGNTTNSNNNLEENIRLSMEELSYHHQQEDVSNYVNGVTATTIDIPHPQHLGLNMGNSYNNNINMDSHLVQHEIDILPYQQPTWDPNVQEMQDMGYTNHSEHQPHDQQFQQTEAQNCSQSYNPSSILDPPYPSQDLLNLLHLPRCSTSSLLANPAICIANKTQNFQNPMGFLGDLPIGSDNTSASSVLYDPLLHLNLPPQPPALRELFQSLPRGYSLPTNSRSGSLFGGGDEIEGDGSQLDMGVLDFNRVTASVGKGREGKGTKHFATEKQRREQLNGKYKILRSLIPSPTKMDRASVVGDAIEYIRELLRTVNELKLLVEKKRYGRERCKRQKAEDDAAESCNIKPFSDPDGCIRTSWLQRKSKDSEVDVRIVDDDVTIKLFQRKKINCLLSVAKVLDELQLELHHVAGGHVGEYCSFLFNSKIIEGSSVYASAIANRVIDVMDTQYAAAVPHTSSY.

>Araip.Q7H3V.1 genotype-assembly-annot=K30076.a1.M1

MSDSTVLEWLRPFVEAKAWDFVVVWKYGDDPTSGWVVAVVGAVVKALRMQRSSKMKKWMKNRTTWILLPFARMHIFSIQLEPRLVAISQQPKWLTHGEEIGTQVLIPVVGGLVELFTTKLVPKDNNILEFIRAHCYVSLKQEAICAQHHTDVNFSENLSSEEQYTQSSPQLASTLTDGVHLLAANWCKSDPYIEEPSSGSNPSSEYTSFDSKFVCLTHHEYLGESVKLSPTCKTERPKYNETSGKQQGTLSSYCGNGKRNKTKSVRVPPKEGYHAKNLATERRRRNKIKNGLFTLSGESGAGKTETTRMLMCYLAYLGGRVATKGRTVEQQVIESNPILEAFVNAKTVRNNNSSPFGKFVEIQFDKNGRISGAAIRTYLLERSRVCQVNDPERNYHYFYLLCAALQKEAIFRVVAAILHLGNIAFTKGKEVDSSFPKDDKTKFHLKTTAELLMCDLAGLEDALVKCVMITRDGLAKTIYSRLFDWLVDKINNSIGQDATSKCLIGVLDIYGFGSFKTNREAARNFEFLLWQWQKKKSVMDRASILADAIDYIKELHGQVEDLKDEVRNLEVEDCEKNTLQSIMPSKEQGENRTLLTELNQRSSNSTKQIEMKMQTEVNHISRTEYLLKVCWEQKPGGFSRLMEAINSFGFQVETANMTTIDGKAQIILTVEAAKEGIHPTKLKDFLTEQTG

>Araip.J3ZJD.1 genotype-assembly-annot=K30076.a1.M1

MDISSLRGLTDLEIMEDPTYLHQWHLSSIDEPTLLPIAAAFGETLQQHSFSHPSFNPKTSMETSLSNIDRPTKHLKNNSWCPTKTPSEAQFASCSTLLSFVDPNYINQLEVVKSKDEMVCPKMNDTTPKDMNFQGTLGNQYVFEASQATKHVGPRSRLSQPQDHIIAERKRREKLSQRFIALSALVPGLQKTDKASVLGDAIKYLKQLQEKVKALEEEQNKRKAVESVVFVKKSQLSNDAEDASLEYEGIFHEALPEIEARFCDKNVLIRIHCEKNRGVVEKSINQIEKLHLKVTNSSAMTFGSWALDITIIAQMEKGFCMTVKELVRNLRSAFASII.

>Araip.8M056.1 genotype-assembly-annot=K30076.a1.M1

MALLEDHHLSSSNDFSNFVVYDTISATPFSSHGSSSTSFLVEENKVEEYGERTTTTTRKRQSGEAGQGKKKRRRKPRVCKNKEEAENQRMIHITVERNRRKLMNEHLAVLRSLMPESYVQRGDQASIVGGAIEFVKELEQLLQCLEAQKLKLVHQGMAAQQSNKNNNEDASSSSNELMMMMMMVMGSAPFSHFFVHPQFTWSQAPNKYASHHNKTNNNNNNQAAMADIEVTLIETHANLRILSRKSPKQLSKLLSGFQNLSLTILHLNVTSMDPLVLYSISAKVEEECQLGSVDDIASAVHNLLKMIEEEDSLCSLYGEVTKSTPSPLQDSLENSNTEYSLS.

>Araip.4U4XR.1 genotype-assembly-annot=K30076.a1.M1

MELSQLGFLEELLLLAPRRVEETCWNNGFNDLLPTTPPPSASVSASWSFDSLDDNPSLNPSFSSFSTPLDHRFDSSYAASFPFLDAFTIPPELHHSPPPLLPQQEHNNNNNNHPLVEEEEEELGFLGNDDNNQSLEERNSGRCKVEEQQALEVPAVVFNMGMCGEKKVPKSKKLEGQPSKNLMAERRRRKRLNDRLSMLRSIVPKISKMDRTSILGDTIDYMKELLERIAKLQEETMEEEGKNQIHLLGISKELKPNEFDVERRDHDTRISICCATKPGLLLSTVNTLEALGLEIHQCVISGFNDFSMQASCSEVAEERNCISTEDVKQALFKNAGYGGRCV.

>Araip.JLE70.1 genotype-assembly-annot=K30076.a1.M1

MESDLHRHPPMFLDHHHHHHHHHHHQQMNTTTTNNNSNSSGLTRFRSAPSSYFSSIIDKEFYESIFNKPSSPETEKILARFMNSLANDEPEDDSLLGVAAATTTTNNNNNNKNLSPPQQVVQQISQVKEEEISINTINTNNTLQPQPLPSSMNNESLVQQPQQQMNSMSNNNYGSLSGNNPPTQSFYQSSGRPPLPNQMKTGRGNASNLIRHGSSPAGFFSNLNIEGYAALRGMGTMGAAASNTSEDANFSPVARLKNPPTFSSSGLMTPIAEIRSTSNTINNPESAEAFAESQSSDFMSGLTVGSWDDSSSSMMSDNIAGRKRFRDEDVKPFAGANVADTQVKTEAGQAPGAPLAHQLSMPNTSSEIAAIEKFLQCSDSVVCKIRAKRGCATHPRSIAERVRRTKISERMRKLQDLVPNMDKQTNTADMLDLAVDYIKELQNQVETLSDSQAKCTCAHKKQQ.

>Araip.510K0.1 genotype-assembly-annot=K30076.a1.M1

MGMQRVRNHYYSGAQILRSSSITPYDYIFTKPRSKAADKKQAAVKHSQAEKRRRMRINTQYEALKNLFQNKTKTDKASLLTTTIEIVKNLRKNSIPQEASSSSKKEMNKVTNQKKMQEACSPRNDDVFPSWEDKWSLKHEEGLMKATLSCEDKPGLMSTIAKAMGSVEAKVVKAEMVNVGGRTRVVLWVKDSNGDRKGEEILKRTLSTLMQRPVPKKRRFTQ.

>Araip.JIB5P.1 genotype-assembly-annot=K30076.a1.M1

MKISMLVGEDQHKKSTKQQRGGGAMKVLICQHERILGRVNYLMEISSELGIMEDPNSFLWHLSSIDTCATTLAVFGDSLQKNNPLFCNSNLMNSKISMMETTTSPTATIIERPAKQLRSNNTSNWSSHINKTPESHFVGSCSNNILSFVDNTNHHHHQLGLVMKPKVEIMNSSSPNNIDTQGTTLLGNNNNNHHNHENYLFKESSCHEAKNFGQRPKLSSHQPHDHIIAERKRREKLSQRFIALSALVPGLKKMDKASVLGDAIKYLKQMQEKVSALEEEQKKKKTVESVVMVKKSQLCNDDEDSCSSETEPLPEIEARFCERNVLVRVHCEKKKGVIENTIIEIEKLHLKVINSSVLTFGTFALDITIIAQMDMEFSMTVKELVKNLRLAFSTFM.

>Araip.AKW6F.1 genotype-assembly-annot=K30076.a1.M1

MDPGAMMNEGSFGNGGGNTVPFSLAEIWPFPQAVNAAGGGALGLRRPQFGLGQFGDFIAGPNREPGPVASEQKAASGGGGGGSRKRRESEEESPKGVSTSNVAPNAVEEEERQASSGKPADQNNQTPPPDPPRQDYIHVRARRGQATDSHSLAERARREKISERMKILQDLVPGCNKVIGKALVLDEIINYIQSLQRQVEFLSMKLEAVNSRLNTGIEAFPPKDVSLQRFLYWDVEFC.

>Araip.AVK7Q.1 genotype-assembly-annot=K30076.a1.M1

MSSSGAMQKKHEENSFLDTKTMAEGSDPSPSPTNMVAAADEGNTENSNEENLNLSLDEFYHQNHNPPPQTTEGVSTFENGTSSISMDIENTQNLSLNIGNSYSNKGTTTPLVQEIVIDHDDAFHYEKSNWDSTIHELLDLGFSNHNENQQAHDHQQFQLCEAQNCSQSYHSSDLLQLHLNPNQIPCITNPIQNNTPNFQHSMGFLGDLPVGSDYDTSLHLNPAFGVGELFPSLPHGYTRLTDSRSGFLFGGGDEMEGNGACYQDGDGSQVDIGVMEFNRARACSVGGRGRRGQGTMPIDKQRREQLNDKYQILRSLIPSPTKVDRASVVGDAIDYIRELIRTVNELKSLVEKKRIGRERFKRHKDENDASESCNIKPYGDGSIRTSWLQRKSKDSEVDVRIIDDEVIIKMVQRKKINCMLFVPKVLDELQLDLHHVAGGHVGEYCSFLFNSKIIEGSSVYASAIANRVIEVMDTQYAAAAVPHNSSY.

>Araip.I1L37.1 genotype-assembly-annot=K30076.a1.M1

MDHIMAERKRRQELTERFIALSATIPGLTKTDKASILRAAIDYVKQLQERVQELEKQNKKRSRESVILVKKSKNINNEKILLEESTIISTSETRRSEDGGTGLPDIEARFMGKDVLIEIHCEKENGIEMKILNQLENLHLFVTGSSVLPFGNSALGITIIAKMGDSCEMTVNDVVRNLREVFLKAHQNSHGI.

>Araip.0158U.1 genotype-assembly-annot=K30076.a1.M1

MALAKDQILHDSSMGSKVQSCVFNENNEYHKSVLEEEDGSQSTNGFSNDSAITHSPPLCANAYAYKATNYQLEEEQQSLIDFKGSCYNTLTQVVSESLLNFEQNRMVPGNSYMKDDTNVWDNNLHHQWSQISPRSTSELRPVQDSSCFQSSSSSYSTIVNSAKEKQLHGESSSYGWLYSQQTIPAHSIQDPAAQEPISKKRISMQAEKMKAAKKQCTDESKMPKSNKSGASKDPQSVAAKNRRERISERLKILQELVPNGSKVDLVTMLEKAISYVKFLQLQVKVLAADEFWPVQGGKAPDISQVKEAIDAILSSQRSEEQVQQPQSRSSWIFYEYYSVIIK.

>Araip.MA0JY.1 genotype-assembly-annot=K30076.a1.M1

MEFLGAFSNNIELDCFKMFSTEKEHDFTYTTTTSQLFLDQRSSLLVGEDDELNFGLVQSTISSNLISDENEHYLFHSLDANPNTKMLHYKSQEESSYNSSNLSGGDTNFFNANMDLTTNYYYSSNYHDDHVLANDVSISMELCMEEKNLACFAPSMEDNDDHGLLENYDHYQCHQMENVDVVQLKRKIVEVPELDVSADENNTSNGYKNQKKKIFVAKDEQDCMKNEKSRKRKVVRNGNEGEERNNNVGLDGQSSSSNINICEDDNASEENNGGVTSVSHSNGKTRTIRGTAADPQSLYARKRRERINARLRILQSLVPNGTKVDISTMLEEAVNYVKFLQLQIKLLSSDDLWMYAPLAYNGLDIGLKLNNLKNFSPSP.

>Araip.7B3CC.1 genotype-assembly-annot=K30076.a1.M1

MDGTAARKSQKADREKIRRDRLNEQFVELGSILDPDRPKNDKATILGDTIQLLKDLTSQVGKLKDEYAALNEESRELAQEKNDLREEKASLKTDIENLNNQYQQRLRNMFPWTAMDHSVMMAPPSYPYPMPMAVPPGSIPLQPYPFFANQNPSVIPNPCSTFVPYLAPNTLVEQQSAQYVSPPSHPGTRSHLSSKHDTRNKPPRDRESKAEKSEASNDVTTNLELKTPGSSADQDLSSGKRKCSKPLRSEGSSLGRCSSSHSVQDSSSSSVDGSRKANE.

>Araip.EQ8VB.1 genotype-assembly-annot=K30076.a1.M1

MFHLFFLPIPRFSTTKHTQIVSEFRFLVFNGSSSRFDQVVAGSRTAKRCFISFNSIMDGLNWDDATSQIIARNNTPILWSKQQQSQEIILTYPNSEHLINYQQQDAATVVKSSAIMSLSENGPYNMDNYYQISGKYMTDFNMAQQHQQHDHHHHHCLISSATNSNTNDDDNDGISKILLSDDDCKNYLWNLSSSTTDAASSAESESNNNNNNDASMLPCSHQNSSDHQQYINQGGQFNPIIITNKDHSPIIKIENPPRSKKPRWEKTPFSSTISFQNPNEASATMSSTSTTEEEADPEAIAQMKEMIYRAAAFRPVNLLDSDEVIEKPKRKNVRVSSDPQTVAARQRREKISQKIRVLQKIVPGGTKMDTASMLDEAANYLKFLRSQVKALENLGNNTTKVEAMDYNNYNSFVFSFNPSFHSQIHF.

>Araip.MX2SJ.1 genotype-assembly-annot=K30076.a1.M1

MENYDHCSLFLDPSQTLPHVSDCNSLHRQFGGEFHSWPAPVEVVAGDRAASASKSHSQAEKRRRDRINAQLTSLRKLIPKSDKMDKAALLGSVIEQVKDLKRKAIEVSKAVTVPTESDEITIEYDPSARDESLKIKNKVVIKASVCCDDRPELFSELIQVLKGLRLTAVRADIASVGGRIKGTFLLCSKDSEKGVCLGTLKHSLKSVVTKIASSSIATNWPTRSKRQKFFLPSHFI.

>Araip.KNC8H.1 genotype-assembly-annot=K30076.a1.M1

MKLNLHLFFFVFFLKQHEHLIIIKGPFYISFDFCSTGLPGEEERLGFNHFHQSKKLENWDDHNILLNNNNNQHNTRVGPNNNNNNNNMIVDGIIKQEVSQSGNLYGQEEFHASNGSTWSTHMVPINSSSPRSSVTTNNNNNNNLLDFSYNKVDHSKNNQLPYATPQVRKEKLGDRITALHQLVSPFGKTDTASVLLEAIGYIRFLQSQIELLNDTGLKRKAASSQDSKDNKAKDLRSRGLCLVPVSFTQHVGNENGADFWAPAYGSGF.

>Araip.K6CZL.1 genotype-assembly-annot=K30076.a1.M1

KFLTITLSLSPPTHIDTLSSLSLTMAMSVENQDPNDIGFFWDNQPWDVSNFDNLGQRETKENLHIEMPPLPPAPTNHLTQGEIEKKNEELTMPVTSNKKRRRADEDGKRPMKNDGNDHDLHIWTERERRKKMRDMFANLHAMLPQLPPKADKSSIVDEAVRHIKTLQQTVENLEKKKRQRIQSLSVSVSPIACESAVTNPQWNPYDSSSLSRDNNALTITATQHVSAAVANNSPFFKTWASQNVVLNMCGEEAQFSICAEKKPSLFTTIAFVLQKHKVDVISASILCNHNVNRYMVLTHASRALLQFADANTVEETFKRAAEEIMMWLG.

>Araip.12TI6.1 genotype-assembly-annot=K30076.a1.M1

MPPTPSFNTLDYSLDHNHHHHHLMQFRQGESSGEHSNGIADYIPQQQQQLPPAPPPSCFYNANSSSFDKLSFADVMQFADFGPKLALNQGKPCEEPGIDPVYFLKFPVLNDKMDEHNIMMMNHESGGCSDGGGGREEVETEAENNNDERFNNGEEEDAAMGGADQEDGRMKGEEETARISEDNNSVQIRFLGHGEDLVLNQKRNNNSTALMQENNKNMKRKRPRTVKTSEEVESQRMTHIAVERNRRKQMNEHLRVLRSLMPGSYVQRGDQASIIGGAIEFVRELEQLLQCLESQKRRRLLGEAQTRQVGDSNLGTQQQQPLQPPFYPPLASPSEQMKLVELETGLREETAESKSCFADVEVKLLGFDAMIKILSRRRPGQLIKTIAALEDLQLIILHTNITTIEQTVLYSFNVKVASESRFTADDIAGSVQQIFIEQEYKII.

>Araip.G8G7Y.1 genotype-assembly-annot=K30076.a1.M1

MENWLSHLEISGGGSGSSSVSFEEILSFNGGDHSSNNKDNNIIIIGDEANKQEKKNSKRGSLGNNNNNKRTTKKARNSIEALDHIMSERKRRQQMAEKFIALAAAIPGLKKIDKASILQEAINYVKELQERIALLEKESNNKEQLIVKKSQFCCSSLPCDKNNNDNNIGGNEMVLPEVEARLLEKEVLIRIHCEKRNGIMLELLAFLENNNLSLACSSFLPFGNYFCFFNKNTFFKRHERGIQLNIERSGEDVEGVSFEVYEMQ.

>Araip.8W8RT.1 genotype-assembly-annot=K30076.a1.M1

MGDRENFEVDRDRDRVNYSTSMASDWRFGVGNFANSMVDSYVTNFWDHPNSQNLGFCDINGGSSNMNVIGKDGFGYGRGSHDHRTLEMGWSHASSVLKGDNNAFLPNGPAMLPQSLSQFPSDSGFIERAARLSCFGAGNSVDMVNSFGIPQSMNLYARIGGTMQGTGDALLGHGLRSAALGGQSHKSDPNVVEDAKDDKEMTLQDDKRSENLPVSHDEGKQALGGSPNESDRAESSGRDDSPMLECTSGEPSNKGLSSKKRKRIGLDADKDKVDGSPEQPGAAVKENSGSRQQKGEQQPTPSTKGSGKNAKQGSQASDSPKEEYIHVRARRGQATNSHSLAERVRREKISERMKFLQDLVPGCSKVTGKAVMLDEIINYVQSLQRQVEFLSMKLATVNPRMDFNIEGLLAKEILQQRAGTSSAHGFPPELSMAFPPLHPSRPGLVHSTLPSMANSSDILRRTLQPNLAHLNGGFKDPNQLPEVWDDELHNVVQMTFPTSAPPSCQDLDGSAPSGQTKTEP.

>Araip.M6R3N.1 genotype-assembly-annot=K30076.a1.M1

MSDKEKFELDRKEDPMNYSTGMASDRRFGGSNLLNSSVGLAGRGDLNGSSSCSSASLVDSFGPNFWDTPTNSQNLGFCGINVHNSGSSLNPIEIRKDGFGFGRGGHDHGTLEMGWNQGNGFIPNESAMFPQNLSRFPSDSGFIERATRFSCFGGGDFGDMVNAYGIPQSMGPYAGTGGEAIHQARDGGQPQESNPNEAEAVKDATPSVEYLATKASPLKNDKRSENHVMPQNEGQQGPSRPPNETDRAKSSDDGGVQDDSPMLDGVSDEPCLKGLDSKKRKRIGQDADNDKAVELPTEGAGDISESQQKGEQEPTPTNKASGKNAKQGTQVSDPPKEEYIHVRARRGQATNSHSLAERVRREKISERMKFLQDLVPGCSKVTGKAVMLDEIINYVQSLQRQVEFLSMKLATVNPRLDFDIEGLIPKDILHQRPGPSALGFPLEMSMAFAPLHPSQPGLIQPTLPSMANSSDILRRTVHPQLASPASGSFKEPNQVPDVWEDELHNVVQMSFATTTAPMSTQDADGTVAATQMKVEL.

>Araip.MY2WL.1 genotype-assembly-annot=K30076.a1.M1

MESDLQAPSSNGLTRYRSAPSSYFTDIIDREFYEHIFNRPSSPETERVFSRFMNSLRDDAAPEDDSLHLDSSSVKEEHDITPSQPLLFQHHQQQQQQNNVNSFNYQSTSRPPLPNQNQNLASSGGVEGVYSNNNNNNNRLPQTKNQSNLVRHSSSPAGLFSQIHIENGYVVDMRGMGTLGAVNKSVEEVKFSSSRTRRLKNPQNYSSSASGRMSSIAEIGNRDSREDNPDAEAFGETQGEDFITELAPWDDSVAVNDIVAGLKRFRDDDVKPFSSGLNAAETQNESRGQQAAPLAHQMSLPNTSAEMAAIEKFLQFSDSVPCKIRAKRGCATHPRSIAERVRRTKISERMRKLQDLVPNMDKQTNTADMLDLAVDYIKDLQKQVETLSDCQAKCTCSSKPQQ.

>Araip.CX0J5.1 genotype-assembly-annot=K30076.a1.M1

MDGVFNLPEATRSDFLRSLLHTFGCTYICLWHCHSSSNNLLFLDGIYNNVSSTVAEETLFNLYQRLTFDAASDEYVGSWCGFQEANGLFRASTIGSSKTCIRSYPNTILSGKEARIERAIFMGCSKGEIELGFSTIPQIDMKAAVKSLFPEDFSRQQIHHPASSSSSSCPSHEFSSLRGVLLPAPEGEQEAIIRAILHVISSSSSPATATSQPPPYNNSAFRAYSSTAYNLVTSSSSSRFSLMKRSIEFSRTLHLMRIRDRFHFQHQHQLPHHPLIIDTQNANNNKHLRHIILERRRRENENKCFRELRALLPPGTKKHKSSVLTAAKEALKSLIAEIEKLNIRNQHGVLSHHRQSDIMIRILEFLKNLQNLSFVSMATNTSTNTQGTTINQIIFRLRILQGSEWDESGFVEAVRRIVSNLIRN.

>Araip.86J2T.1 genotype-assembly-annot=K30076.a1.M1

MRKLALQAFQRQQAPNPVSVTALPQQPPGIRPRVRARRGQATDPHSIAERLRRERIAERMKALQELVPSINKTDRAAMLDEIVDYVKFLRLQVKVLSMSRLGGAGAVAQLVADVPLSAVEQGEEIEGGGNNEQAWEKWSNDGIEQQVAKLMEEDVGAAMQFLQSKALCIMPISLASAIFRMPPSDPSSLIKPESNSHS.

>Araip.52XKK.1 genotype-assembly-annot=K30076.a1.M1

MESKMSFTSGGTSCHVTGSAAEDDSNMQDAVATEEGSGSQWSFLPKPSHHNYVQYLTESSNYLPKTEDMIGSNYAALGFGNDGNGTNMNFFCNDPGKAYAFKTEVHSSNEVDIGKQVGYWRPAESEVQQEAMQFESTQHHHPLYPESPASWTPEAIGDNPNVSRLDPSVLAGTPSFLPKPARQKASDRQRRQRIADNLKALHDLLPNQAEGSQAYILDDIIDHVKYLQLQLKELSGSRLQAESTAIPLVFHEGYGHYIDQQKLNEPLEEMMGKLLEENPASASQLLENNGLVLLPMELVQDLHQSMQIFGNGNALV.

>Araip.K2CBN.1 genotype-assembly-annot=K30076.a1.M1

MALKAVVYPQSQDPSFGYGVNYNNCYRNWSNSYNNEDQRVETTYPAEIWNNNNTTCSSPPSVLPSSCRPKRRRAKSRKNKEQIENQRMTHIAVERNRRKQMNHYLSLLRSLMPDSYVQRGDQASIIGGAINFVKELEQKLQFLGATYQEGEDGNNKKKKNMAFCEFFTFPQYTRTATATATTSSSSSNSEKSSSSRTVAAEEELGEVAADIEVTMVESHANVKIRSKKKPKQLLKMVSALHNMRLTILHLNVEDDCKLGSVDDIAAAVYQLMNTIQQEAPIILASTNFKIQASSLISFITKFGS.

>Araip.7G5H2.1 genotype-assembly-annot=K30076.a1.M1

MMPLHELYRKAKEKLDCSKEINSTSAPDHSTAPESDFYELVWENGQISMQGQSSSRGRKIPTCKSLTSHCPKGLQHRDVVGYGNGTNNVNMMRMGKFGDSESGLNEIRMPAPSAEDEDVIHWLNYGMDESLPHDYGSDFIHELSGVTMHEIPPLNNLSLLDKRSNSNQVLRDSHKNYARHAFGSEQGILNKDFSVMARGEIEIPGPKPSTSQFCQPSSYQCQGSFASVRSKASEITENNGSNPAHQVPCGELTQIFPSASSGFSGLKLDKQDQVMCSSSSTIMNFSHFARPAAIVKANLQNIGLSSSRSDGIENKKKDASATASNPPESTKAGFSGERQKQSAVHELKVVEPSKADLKQLEPSKADLKQLEPKSLEVNATVLRQSDPARKEDVSKIYQSSNLLLCESTNKGEEAVVKNMEPAVASSSVCSGNGAERISGNPNQSLKRKRQETEDSECHSEDVEEESVDVKKAGPTRGVKRSRSAEVHNLSERRRRDRINEKMRALQDLIPNCNKVDKASMLDEAIELCQWELVPQMHRTHPPAAPMPGPSALHGMGRSNPPVFGLPSQGLPIPTMPRAPMFSYQGEPVANSPALRPNACGTAGLTETENPASASNQKDPVPIMQNTNGCNSTSQTTKQCEAAAAGRAVDATKKDNLVTDKFD.

>Araip.M4TVL.1 genotype-assembly-annot=K30076.a1.M1

MERLQLQGPLDSSLFGEHLEVNCLEQGFVDRESNFKVKEDEHEEQTLISSLEGNMPFLQMLQSVESPPFSPFKDPNFQALLRPQHLKKPWEGTPYIPTLESEINEFEQYTRSGSDAAWANKKTNSQHKDTRLSKHHHQERRMTHIAVERNRRRQMNEHLSVLKSLMHPSYIQRGDHASIIGGAIDFVKELEQLVETLEAQRRMRNKEEEDGGMRSSASSSRKEEASGGNEAKAERRSKVGGIEVSVIQSHVNLRIQCEKRAGLLINAIVALENLRLPILHLNITSSDSSVLYSFSLKIEEDSKLGSANEIAEAVDKILTSLTH.

>Araip.Q7CA9.1 genotype-assembly-annot=K30076.a1.M1

MVAGDRAVSASKSHSQAEKRRRDRINAQLTSLRKLIPKSDKCNKLILHMDKAALLGSVIEQVKDLKRKAIEVSKVSTESDKIVKGREINLKVVASKRLLYYEFDFRAHDSAFKDRYLQFQNVRVRFIPTEKKDIRDIYAAPNQRPTIYDMHEKTVDEKYYYSFEYVLTSPNYLSASFATIAIGNGRYWPLIVGANERRWKRYRD.

>Araip.EFK2L.1 genotype-assembly-annot=K30076.a1.M1

METEKLPAMDNNTLLSETFLNTREFGGEDLFSILESLEDFNSPPPPPPRKRQKVTASASEDGQERVLSSHITVERNRRKQMNHHLSGDQASIIGGVVDYINELQQVLQCLEAKKHRKVFYTDNVLSPRLIIPSPKLSPRKPPLSPRILTLPPISPRTPQPGSPWLHSIEPSPSSSGSSAMNDNMNELVANSKSVIADVEVKFCGPHVLVKTVSPRIPGQAMRIVSALQDLALEILHLTITTSDETMLYSFTIKIGIECQLSAEELAQHIQQTFC.

>Araip.W6M4V.1 genotype-assembly-annot=K30076.a1.M1

MEQATGVLRGSTHDDVVLESIQFNEEIRGIMAPAPAPENASSFTALLELPPTQAVELLHSPDCPAAAREPPCHVSTNQKPYLLCSYGGNLTFPSNAALIERAAKFSVFAGENSPPEDAARLAPVAVSGVVKNEPQETDSNPSSTQGCVSDPAVDTKNQKTAKRKEREKKARREKINARMKLLQELVPGCNKVGFPFLVVISGTALVLDEIINHVQSLQRQVEFLSMKLAAVNPRIDFSLDSLLATDGSSVMDSNLTSMASPVMWPENLANGNRQHFQQQWQFDAFHQPLWGREEANHNFMTPEHSLLSYDSSANSASLQSNQLKMEL.

>Araip.PFD9B.1 genotype-assembly-annot=K30076.a1.M1

AGHQGEGERGVAVASNKDHHHGCDQPHCYLIDLAVVVLLYSFLLFHLILPKEEGAKENQLHGESSSYGWLYSQQTIPAHSIQDPAVQEPISKTHISMQAEKMKASKKQCTNESKMPKSNKSEASKDPQSVAAKNRKEGISERLNILQELVPNGSKVDLVIMLEKAISYVKFLQLQVKVLAANEFLPVQGGKVPDISQAKEAIHAILSSQRLEKQVQQPQSR

>Araip.N5MMK.1 genotype-assembly-annot=K30076.a1.M1

MNPLMGENMFNSSRTGMTRSSSLLHVNKDIGELVWENGEVKVQGRGAAVEERAAKLERFYCSSILDQSKKQIQQGFSNFKLISNTYSSSLSQQCKKPRIIDFSGSAQATTKSHTLQHLGTCNNNNNQRTSLGGGMVNFPNFLVPSLLLNKSSSAAAAATSSKGHDHSSVVIDSSNNNKAATQELKETPFHQVQPLDRHSHAQKPYHGASSSSAAPPPPNYNSNNNIEQHLVVASSPVSSIGASNDPDIGIMRKQHEEYSNITDHDHDDTTTYISDDDDEETEDVIIAKETPAAGTRAKRSRDPEVHNLSDRVNKRIHTLKELIPNCNKIDKASTLDDAIDYLKTLKLQLQIMSMGRGLCMPLMMLHNHHNQLMCYRPSSGTGIPHQNMFDGFFNQMRPMIMPPSPLFINPTPPIAPLSIANSSSLDVSHVQAINNGDQVSLHQHTTSSSYPFYFPTIINQEEKNNYGME.

>Araip.VCZ4R.1 genotype-assembly-annot=K30076.a1.M1

MEPAQLISEEWGSLSGLYTAEEAEFMTQLLAGNYSVSEKHYSSVAMPPSSAFWPCHESTKVSFKGINGNSYLPPSHIENANYLCFSQGSSSSTENGNIYSYDTTTNFDSMSTDNCLEGAKFSPQSNDNSRAQISGIIDEDAKIECERMVFEPAEDLENPTKRLRSSSEVPRNMRNVKSRKENPLPSSYTSNSEEDKGPAAPKNRRSSSGAATDPQSLYARKRRERINERLRILQTLVPNGTKVDISTMLEEAVHYVKFLQLQIKLLSSDDLWIFPRKKMEPGEEIYAEWSSLSGSYTAEEADFMNQFLSNCSHTQQLHANWNAGIPSALWYGHADINSSSFYAADASNNMFPIIDSDNLNDPLTNLVDPSLSTYTDKELGVDVIADKNVPSEPVSEPAQENITSKLEKPRKRSRSSNEILKNKKNVKTTKKPKSASISNIKEEDRCPGQGENLSCSCSEDDLDTCRELNEGESSSLSLKYSKGLQLNGKSRSSRGSATDPQSIYARRRRERINERLRILQNLVPNGTKVDISTMLEEAVQYVKFLQLQIKLLSSDDLWMYAPIAYNGMNIGLDFNITTPK.

>Araip.RMK33.1 genotype-assembly-annot=K30076.a1.M1

MALSTYLNGDALQSSIISEIFTTKFQELTTPEELTLDHFYHQHSQEGLFSNNFFFDPYFDFNNGFFHPEILSSHQLGHSCTSSHDPFISPKPNNLFQIEYSNVNTLLSCPKRQKYFHKDEELQLSSPFKEYASPSLFDGFILNSSSLLPSEEAALAEELLLPAPVTTPSSDFMVPNVVRNGFCVGINNESQKKDSKRTISAQSIAARERRRNITEKTQELGKLVPGSSKMNTAEMLHAASKYVKYLQTQIGMLQLMNTLQKEDEVVPPSEDLCALITSPFVQEKLYSEELCFVPKDFVTTLTNQRDVRSKPTIFKDLKELIETNNVQKKA.

>Araip.2A2GH.1 genotype-assembly-annot=K30076.a1.M1

MILSLRVLLISSHTSTCCFFSLPLNMDIDSSGGTSNWLYDYGFDNISVADFMAPDSAAFTWLPQPQPHTNFKPPSSNISLEMEYSVDSIVLEGGPSKRFLELSSILEPGSMQPKTDKVAILSDAVRVVNQLRDEAEKLKEMNDEMLEKIKELKAEKNELRDEKNRLKVEREKLEQQFKMTNVQPSFLHHAPAATKAQGASQKLIPFIGYPGIAMWQFMPPAAVDTSKDHLLRPPVA.

>Araip.ERC12.1 genotype-assembly-annot=K30076.a1.M1

MKGDSECGGSSMPAERNRRLKMNRLFSQLQATIPRPLSKATKEVIITETIRYIKELERKKNNLEQIKELQIQSHASGTTFMLPCMANDDNCSVTVTVSANVAFFGIQTVARRGLITMILEVFSNHKAEILAANVAVNEGILTFAVTALLQIVADGEGEGEGAVEMIKREIMTL.

>Araip.RM65A.1 genotype-assembly-annot=K30076.a1.M1

MMELPQARSFGTEGRKPTHDFLSLYSNSTSQQDPRPPSQGSYLKTHDFLRVETKANTKEEASDETSSTVQKLPPAAPQPPPSVEHHQHLLPGGIAMKTPTAVLIPQVVSLYGMSLQLRRERQGRRIILWATSLFPEASPRADGSINLETQSAKQGHWTSTEGTPQSFSNNRHNSFNSRSSSQTTGQRNQSFIEMMKSAKDCAQDEELDNEETYFLKKESSNPERAELRVKVDGKSSDQKPNTPRSKHSATEQRRRSKINDRRVLPILHKIFQMLRELIPHSDQKRDKASFLLEVIEYIHFLQEKVHKYEGSFQGWNHEPEKLMPWRNNDKPAESFEACGTNSGSGPSPTLLFASKIDEKNITLSPPITGSSQKLEPGLNTATPFKNMDHPPGITNNTFPIPISSPPNFFPPRKSIGLGEMPHVTHRLPSDAGNGIYQPSVECQTVTATNEKLKEKELAIEGGAISISSVYSKGLLDTLTHALQSSGVDLSQASISVQIELRKQENIIRPTLPMSMCGIKGDEVPSNNQRMMRSRVATSGKSDQPVKKLKSCKT.

>Araip.DYV42.1 genotype-assembly-annot=K30076.a1.M1

MDGLSWDLSSQLAANNLAMLEGEEDYSIFVNNHMQEMHKSHHRHGYPNSEKVMMMMMSNMVQYPSSATPEAEIVSFPQRTRNNNNNFSMAVKSSSSSSTGWDVKEALTSNHQSWPSLSVVNYVPDFNMAHHHQQQQQQQLINSTTQSSSSLESLDCLLSATNSNTDTTTATTSLEEDDGISIILSDCRNFWNFTTNYGAITAAAAAASSAESETNNASNKQNNMQYHHHHQINNNELDETVSQASKRTIINDQYDLIKTSSAAAATTTTGPYFSIFHNSSSSATESGAFKLIKENPLPRSKKPRWENNNNNNNNNKRGGSSSNINFQQPNSSHSSIDEPDPEAIAQMKEMIYRAAAFRPVNLGLEVAEKPKRKNVKISSDPQTVAARQRREKISERIRVLQKIVPGGSKMDTASMLDEAANYLKFLRSQVKALESLGNKLDTMNNHNNVPPTTIAFSFNPSFPIMPSYFPNPKP.

>Araip.W1EXI.1 genotype-assembly-annot=K30076.a1.M1

MAEGCGGNSVATTSPASFNWWYLHANHATNTNAYNNHHHHQTNPNNSSSCSCEEDISVSTSFTNASNHSTLTVDSSRRLLDPSSNNNHFLPEHAEDNHLWNHVLSAVGSNGELHNNEEMGEMKFLDALSSKNMTSSMFEEPACDYLKKLDTTTSWEYSGGSTSSSFNNSFEKHLNNNNNNNGFSDDALIENNERLTKLSNLVSTWSIAPPDPEVSSSHFDHPPTNNNHHQFPQSHHHHDPNCNFKQQVFIGDSTSYPPISSYDHPTKVKEEFHHHQTNSGFQNGLINGLSSVMGGDSGNKFYHGLPNLSSCTRNISDVISFNSRLGRPVLGIHAQKPNIKYMNNLSESKKQQGLQTTPPINRSNNGRVEGTTAREVKKKRSEESSEAILKKPKQDTSTASSTKVQAPKVKLGDKITALQQIVSPFGKTDTASVLFEAIGYIKFLQEQVQLLSNPYLKSNSHKDPWGSLDRKDHHKEDAKLDLRTRGLCLVPTSCTPLIYRENSGPDYWTPAYRGCLYR.

>Araip.X394B.1 genotype-assembly-annot=K30076.a1.M1

MEAKVDFEVDNLTTATGSSSFSQLLFGDDDDHHQHALGFAAAVDHLPLFSIDKPPKMLSFGDFQPHLLLPETNATPQKSVITSSDSSSASSCNHTTTAINSMSKTNKKRNWPGLQPIVKVPNSNNKKNKTENPPSSTSSGHAKKKEKLGERIAALQQLVSPFGKTDTASVLHEAMGYIRFLHDQVQGEEEEQVKEDKRDLKSMGLCLIPLQSTLHVSSTNGADFWSPAGAANNLTPYAHHQSK.

>Araip.1B22D.1 genotype-assembly-annot=K30076.a1.M1

MKPVIIKMFISITMEIETEGSGDEMKNSSCSALDLYSKAVVVEENGNHSGGGNGNLSLSGGVNGGDQKGKKKGMPVKNLMAERWHRKKLNDRLYMLRSVVPKISKMDRASILGDAIDYLRELLQWINDLYNELESTPPGPSLQSSTTSLQPLTPTPQTLPCRVKEELYPGVLPSPKNQLAKRLFAIIYHAEIVCLRRTVLDEKLKCKEYSTAALIGTLLYQNFQDGLIDFLKTIHLLISLKVTQKWQLLRNIESLYACGGVNL.

>Araip.H4PZ4.1 genotype-assembly-annot=K30076.a1.M1

MYQPSSSSSSSSQAHNSTVSEPALTRYGSAPGSLLRTAVDAVIAGGAGGPRHPHPLMPTGSHYFSGESSESTSKVNKTIGKTVNWIIVIIVVRGGGGSGGGNYGSNSGRLKSQMSFTGQDSLSQISEVSESVVVDASTSERAYAMDHCWDNSNSNSSSIVFSAPPSKRSKNIDGDILNCLTALDSQYSLPQTALEMATMEKLMHIPEDSVPCKIRAKRGCATHPRSIAERERRTRISGKLKKLQELVPNMDKYMPHTMKPQTSYADMLDLAVQHIKGLQTQVQVRKNILDNCTCGCKPSS.

>Araip.RX20Z.1 genotype-assembly-annot=K30076.a1.M1

MAHQQRGQASSSTKMERKIIERNRREQMKNLCFKLNSLLPNFNPKQTLPPRVDQIDEAIIYIKTLESRVEIAKEKKESLLNKGMKKRPHDLAGSSAFDDETQGTSLKPPTIEIHERGSMLEIVLMISGFDNQFIFSEIIRILHEENIEVLSAISSRAGDSMIHVVHAENHLYQLGATKEISERLKRFVDAHCLQDIQGAQGP.
