# Supplementary material for: Genome-wide analysis of basic/helix-loop-helix gene family in peanut and assessment of its roles in pod development
Source: PLoS One. 2017 Jul 27;12(7):e0181843. doi: 10.1371/journal.pone.0181843 (PMC5531549; doi:10.1371/journal.pone.0181843)
Supplement: S4 Table — (DOCX) [file pone.0181843.s004.docx]

**Supplementary Table 4. Information of PIFs identified in wild AA- and BB-subgenome.**

| Genome | Gene name | Chromosome location | ORF length (bp) | No. of exons | Length (aa) | pI | Mw (Da) |
| --- | --- | --- | --- | --- | --- | --- | --- |
| Wild AA | **Aradu.QV5DJ** | **A09 21127504-21132997** | **2199** | **8** | **732** | **5.47** | **78974.7** |
| Wild BB | **Araip.2LX3X** | **B09 27025449-27031056** | **2271** | **9** | **756** | **5.33** | **81439.55** |
| Wild AA | **Aradu.YAX06** | **A06 6451194-6453590** | **1425** | **5** | **474** | **6.44** | **52428.3** |
| Wild BB | **Araip.N5MMK** | **B06 10356347-10358999** | **1410** | **5** | **469** | **6.53** | **51541.26** |
| Wild AA | **Aradu.0DZ84** | **A06 6003593-6007342** | **2223** | **7** | **740** | **6.75** | **78027.3** |
| Wild BB | **Araip.7G5H2** | **B06 11045662-11049490** | **1989** | **8** | **662** | **6.34** | **72126.53** |
| Wild AA | **Aradu.I92X3** | **A07 4395707-4403324** | **1659** | **8** | **552** | **7.60** | **60788.81** |
| Wild BB | **Araip.L4GEP** | **B07 4261196-4268478** | **1659** | **8** | **552** | **7.21** | **60765.7** |
| Wild AA | **Aradu.RC5BB** | **A08 49042357-49045764** | **1110** | **6** | **369** | **9.45** | **41095.36** |
| Wild BB | **Araip.K6RXL** | **B10 1044901-1070158** | **1305** | **12** | **434** | **9.81** | **48770.2** |
| Wild AA | **Aradu.LP0MC** | **A06 101763848-101768612** | **1365** | **4** | **454** | **7.62** | **48697.47** |
| Wild BB | **Araip.2U2B9** | **B06 125913364-125917835** | **1452** | **5** | **483** | **8.79** | **52084.5** |
