# Supplementary figures and images for: Genome-wide analysis of basic/helix-loop-helix gene family in peanut and assessment of its roles in pod development
Source: PLoS One. 2017 Jul 27;12(7):e0181843. doi: 10.1371/journal.pone.0181843 (PMC5531549; doi:10.1371/journal.pone.0181843)

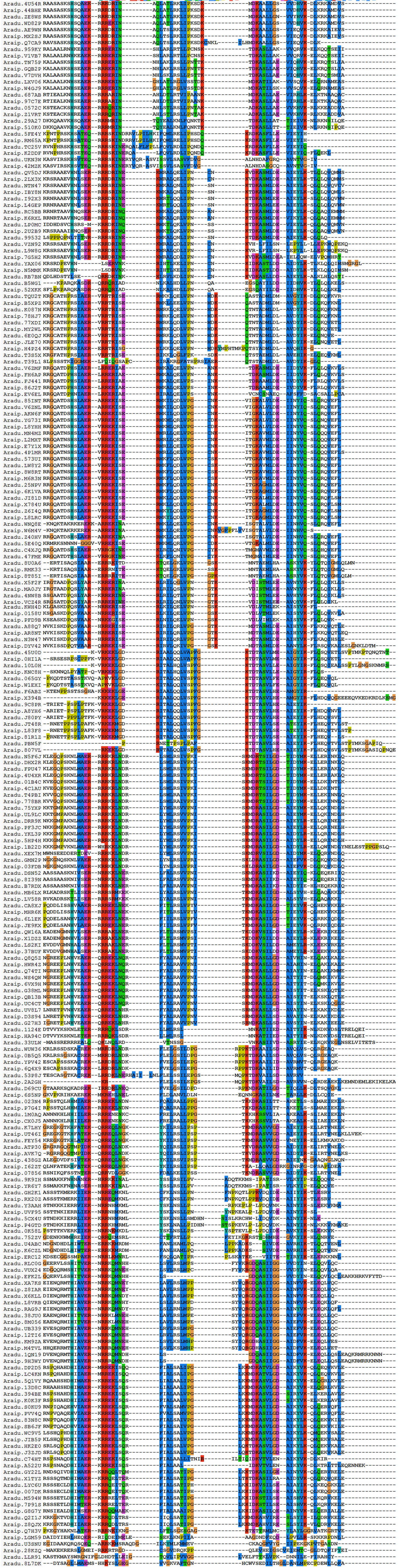

Supplement: S1 Fig — (TIFF) [file pone.0181843.s007.tiff]

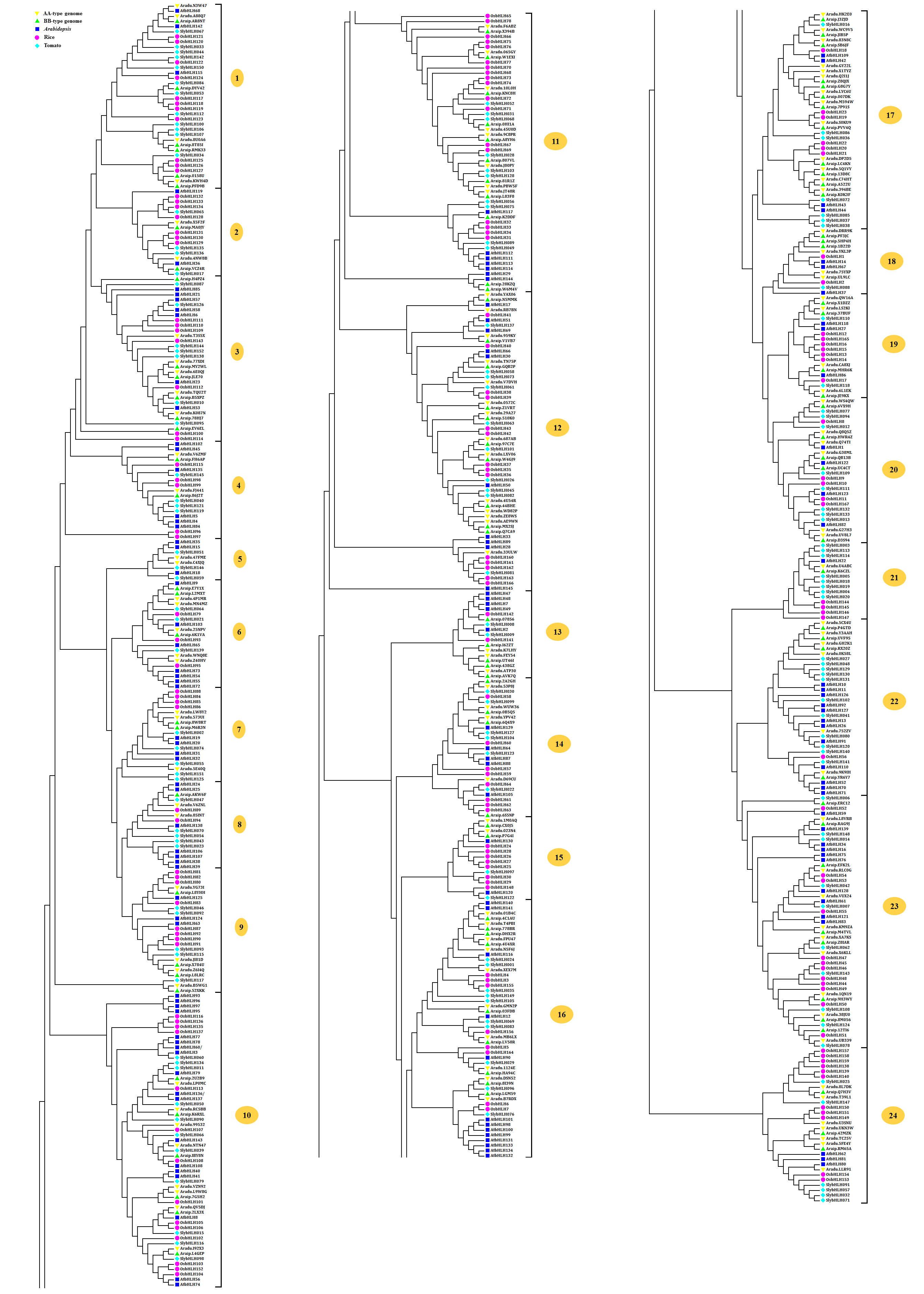

Supplement: S2 Fig — (TIFF) [file pone.0181843.s008.tiff]

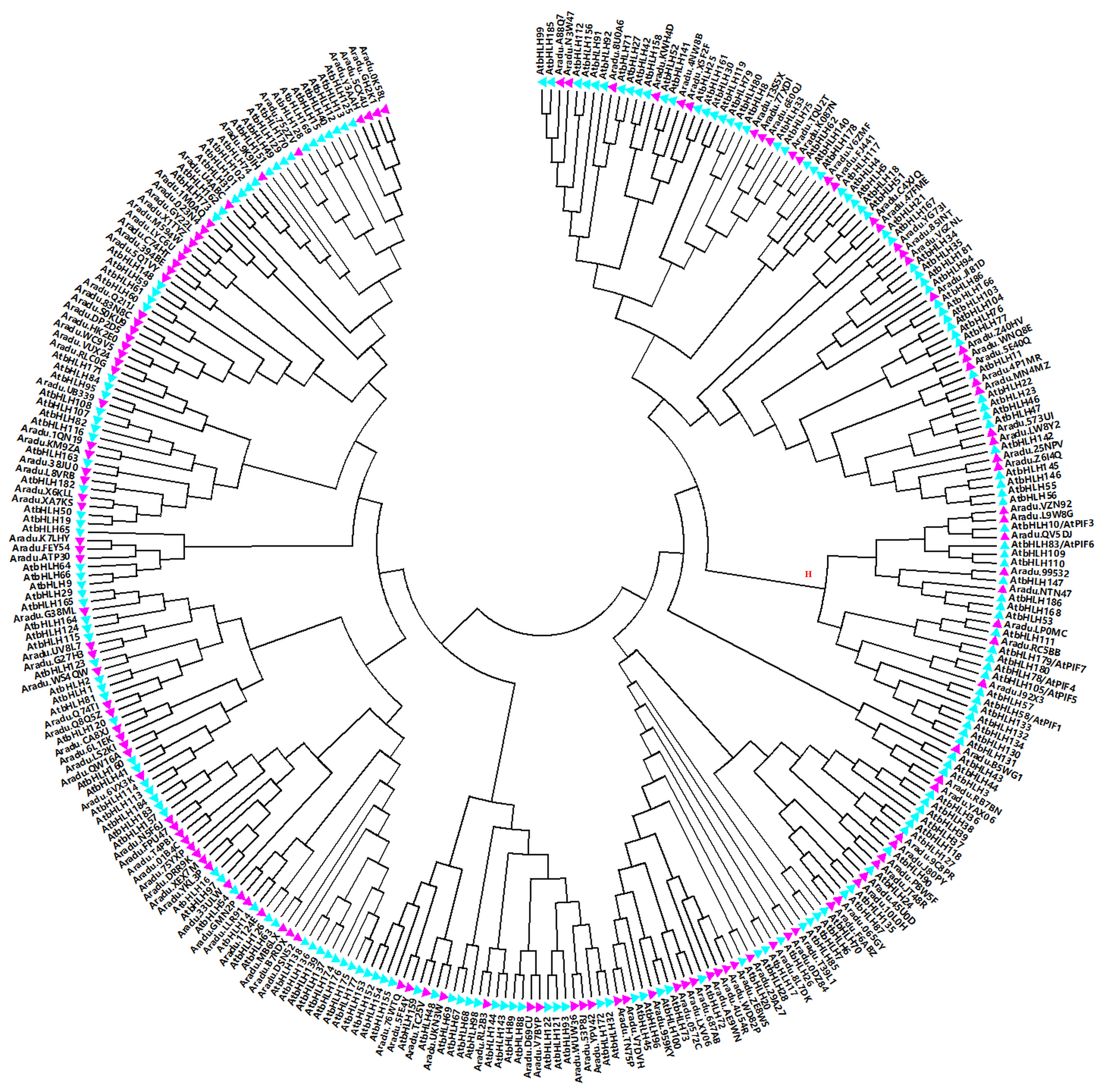

Supplement: S3 Fig — (TIFF) [file pone.0181843.s009.tiff]

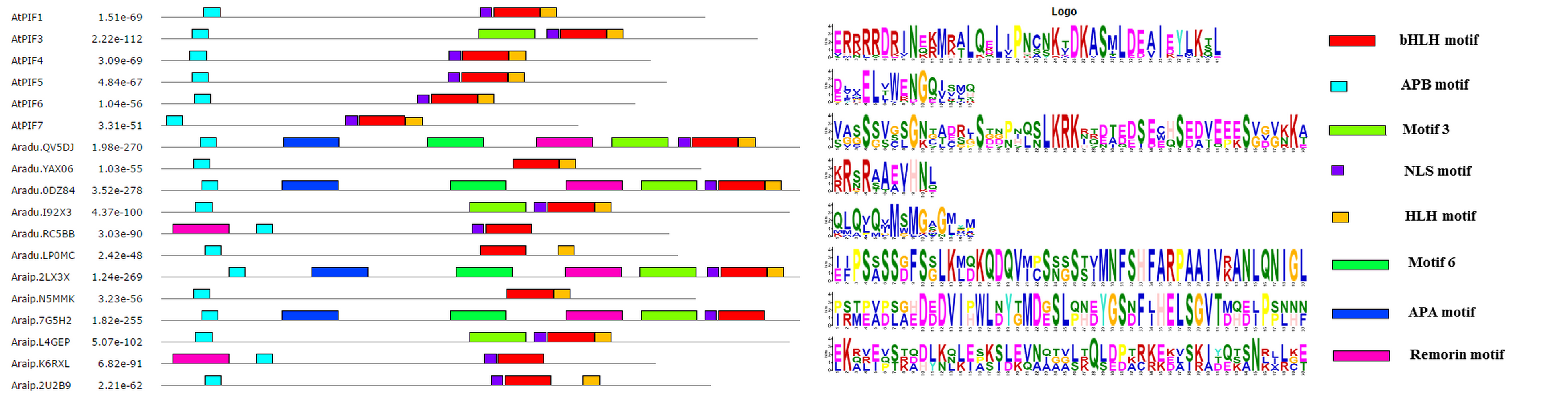

Supplement: S4 Fig — The relative positions of each conserved motif within the PIF protein are shown in color. (TIFF) [file pone.0181843.s010.tiff]

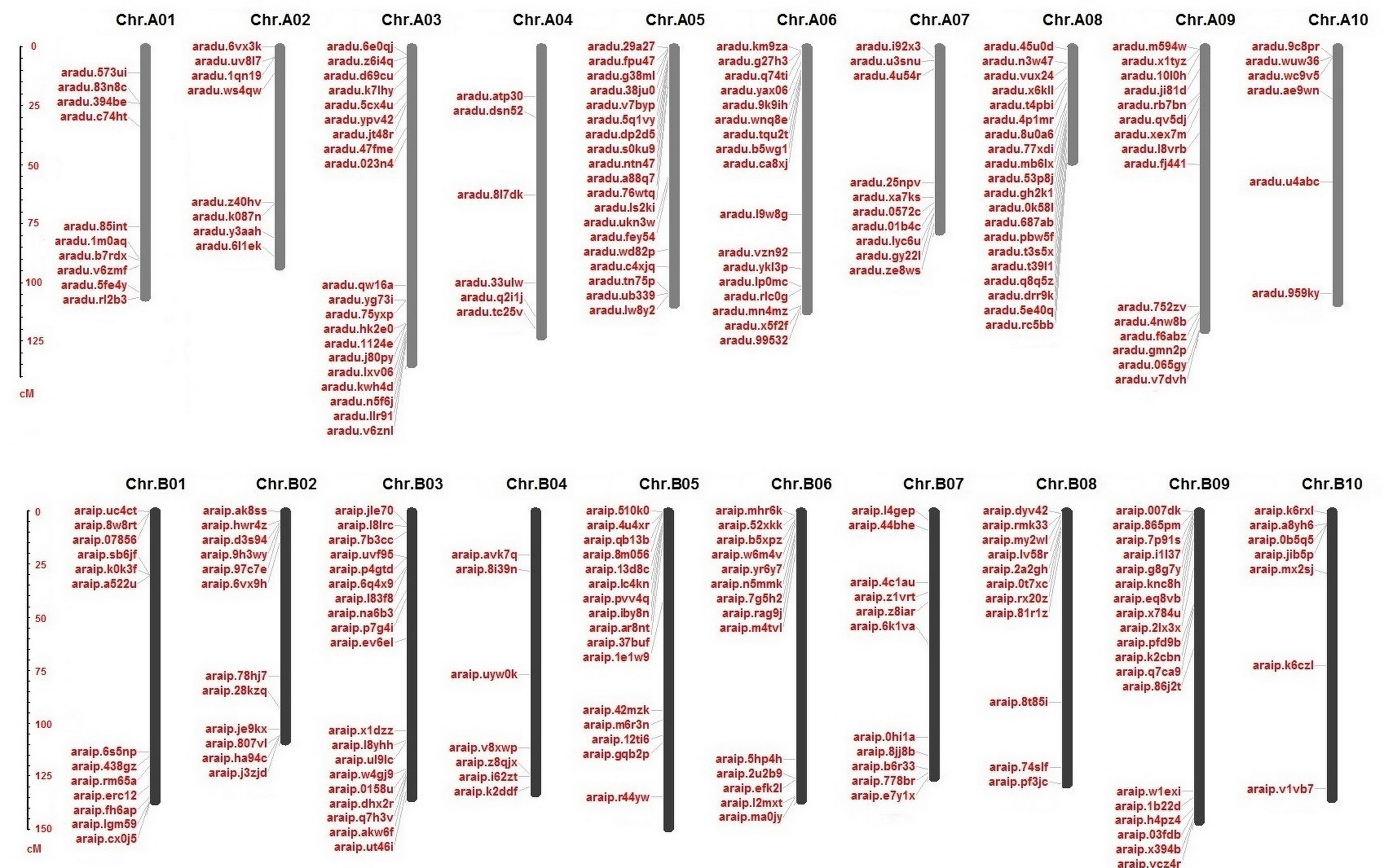

Supplement: S5 Fig — The chromosome numbers are shown at the top of each chromosome (black bars). The location of each bHLH gene is indicated by a line. (TIFF) [file pone.0181843.s011.tiff]
